# Widely Electronically Tunable 2,6‐Disubstituted Dithieno[1,4]thiazines—Electron‐Rich Fluorophores Up to Intense NIR Emission

**DOI:** 10.1002/chem.202000859

**Published:** 2020-09-03

**Authors:** Lars May, Thomas J. J. Müller

**Affiliations:** ^1^ Institut für Organische Chemie und Makromolekulare Chemie Heinrich-Heine-Universität Düsseldorf Universitätsstrasse 1 40225 Düsseldorf Germany

**Keywords:** fluorophores, multicomponent reactions, NIR fluorescence, redox systems, structure–property relationships

## Abstract

2,6‐Difunctionalized dithieno[1,4]thiazines were efficiently synthesized by (pseudo)five‐ or (pseudo)three‐component one‐pot processes based on lithiation‐electrophilic trapping sequences. As supported by structure–property relationships, the thiophene anellation mode predominantly controls the photophysical and electrochemical properties and the electronic structures (as obtained by DFT calculations). From molecular geometries and redox potentials to fluorescence quantum yields in solution, the interaction of the dithieno[1,4]thiazine‐core with the substituents causes striking differences within the series of regioisomers. Most interestingly, strong acceptors introduced in *anti*–*anti* dithieno[1,4]thiazines nearly induce a planarization of the ground‐state geometry and a highly intense NIR fluorescence (*Φ_F_*=0.52), whereas an equally substituted *syn*–*syn* dithieno[1,4]thiazine exhibits a much stronger folded molecular structure and fluoresces poorly (*Φ_F_*=0.01). In essence, electrochemical and photophysical properties of dithieno[1,4]thiazines can be tuned widely and outscore the compared phenothiazine with cathodically shifted oxidation potentials and redshifted and more intense absorption bands.

## Graphical abstract

**Promising donor systems**: Four isomeric dithieno[1,4]thiazines, thieno congeners of phenothiazines, are synthesized by inter‐intra‐molecular Buchwald–Hartwig coupling. Their electronic properties open new avenues as donor systems for molecular electronics.

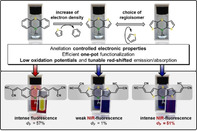

## Introduction

**Scheme 1:**
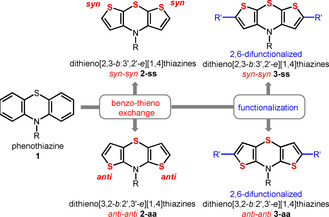
Tuning of the widely used phenothiazine moiety **1**: gaining access to improved electronic properties by construction of the dithieno[1,4]thiazines **2** by topological benzo‐thieno exchange and further 2,6‐difunctionalization of the dithieno[1,4]thiazine core.

Phenothiazines have become particularly attractive in numerous applications in organic electronics due to their outstanding electronic properties: fully reversible one‐electron oxidations at low potentials,^1^ rather unusual among organic compounds on the one hand, and furthermore, luminescence, rather unusual in combination with redox activity on the other hand. Advantageously, redox potentials and luminescence can both be fine‐tuned by substitution on the phenothiazine core.^2^ Hence the phenothiazine structure motif has been implemented diversely in organic light emitting diodes (OLEDs),^3^ redox switchable luminophores,^4^ and Grätzel‐type sensitizers for photovoltaics.^5^ Moreover, bulk‐heterojunction (BHJ) solar cells have been constructed based on phenothiazines.^6^ Thiophenes, electron‐rich heterocycles, are prominent scaffolds in molecular electronics due to a high polarizability accompanied by favored charge transport.^7^ Consequently, electron‐enriched, highly polarizable phenothiazine analogues, namely dithieno[1,4]‐thiazines **2**, were conceptualized by topological benzo‐thieno exchange (Scheme 1). Most remarkably, these small structural changes of the phenothiazine mother system **1** strongly affect the electronic structure. For instance, compared to phenothiazines, the oxidation potentials of dithieno[1,4]thiazines **2** are drastically shifted cathodically and the radical cations formed by oxidation are more stable by several orders of magnitudes, depending on the mode of thiazine‐thiophene anellation. Accordingly, the oxidizability and polarizability of the regioisomeric dithieno[1,4]thiazines **2** differ distinctively. In addition to established dithieno[2,3‐*b*:3′,2′‐*e*][1,4]thiazines (**2‐ss**, *syn–syn* isomer),^8^ in particular, dithieno[3,2‐*b*:2′,3′‐*e*][1,4]thiazines (**2‐aa**, *anti–anti* isomer) aroused our interest due to even lower oxidation potentials and better stabilized radical cations.^9^ Similarly to phenothiazines, the electronic properties of *syn–syn* dithieno[1,4]thiazines **2‐ss** can be widely fine‐tuned by substitution.^8^, ^10^ However, functionalized *anti–anti* dithieno[1,4]thiazines **2‐aa** have not been investigated yet.

Herein, we present efficient one‐pot syntheses of six novel 2,6‐disubstituted *syn–syn* and *anti–anti* dithieno[1,4]thiazines **3** as well as a comparative study on their ground and excited state electronic properties and electronic structures. For comparison, underlining the potential applicability of dithieno[1,4]thiazines as phenothiazine substitutes in molecular electronics a 3,7‐diacceptorsubstituted phenothiazine was prepared and studied.

## Results and Discussion

### Synthesis

**Scheme 2:**
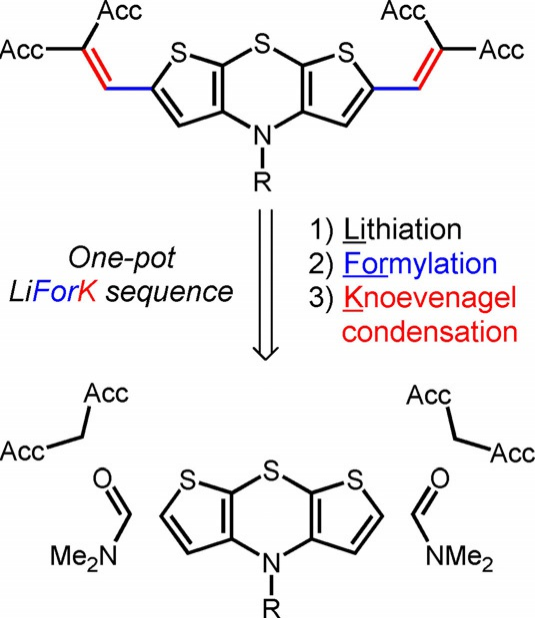
Retrosynthetic analysis of a (pseudo)five‐component one‐pot synthesis of 2,6‐diacceptor‐substituted dithieno[2,3‐*b*:3′,2′‐*e*][1,4]thiazines in the sense of a lithiation‐formylation‐Knoevenagel‐condensation (LiForK) sequence.

The synthetic one‐pot strategy is founded on the inherent α‐acidity of thiophenes warranting direct dilithiation of unfunctionalized dithieno[2,3‐*b*:3′,2′‐*e*][1,4]thiazines.^10^ Multicomponent reactions, in which several compounds are reacted in a one‐pot fashion, have become a valuable tool for accelerated and more sustainable diversity oriented syntheses of complex functional molecules.^11^ Just recently we established a fast and efficient multicomponent access to diversely acceptor‐substituted thiophenes based on lithiation‐electrophile trapping sequences,^12^ we now set out for one‐pot pseudo five‐component syntheses of 2,6‐diacceptor‐substituted dithieno[1,4]‐thiazines (Scheme 2).

**Scheme 3:**
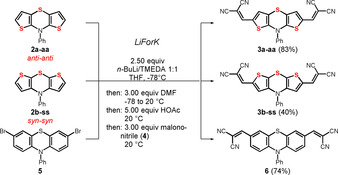
One‐pot LiForK synthesis of the 2,6‐dimalononitrile‐acceptor substituted *anti–anti* (**3 a‐aa**) and *syn–syn* (**3 b‐ss**) dithieno[1,4]thiazines and the corresponding phenothiazine **6** by a consecutive (pseudo)five‐component process.

Hence, reaction conditions of the previously reported lithiation‐formylation‐Knoevenagel‐condensation (LiForK) sequence^12^ were applied for functionalizing dithieno[1,4]thiazines in a one‐pot fashion. For this comparative study, both *anti–anti* and *syn–syn* dithieno[1,4]thiazine isomers of all targets were synthesized. The regioisomeric dithieno[1,4]thiazines **2 a‐aa** and **2 b‐ss** as starting materials were efficiently accessed employing previously reported twofold intermolecular‐intramolecular Buchwald‐Hartwig aminations with aniline.^8^, ^9^ Dilithiation of **2 a‐aa** and **2 b‐ss** with *n*BuLi/TMEDA in THF followed by trapping with DMF and buffering with acetic acid selectively gave the isomeric dialdehydes as intermediates. Upon addition of malononitrile (**4**) the dithieno[1,4]thiazinyl dialdehydes are smoothly converted in a one‐pot fashion into the 2,6‐diacceptor‐substituted dithieno[1,4]thiazines **3** by a concluding Knoevenagel condensation with malononitrile (**4**) at ambient temperature (Scheme 3). In a similar fashion 3,7‐dibromo‐10‐phenyl‐10*H*‐phenothiazine (**5**),^13^ however, by bromine‐lithium exchange, was reacted in the LiForK sequence to give the phenothiazine derivative **6**. This type of donor‐acceptor conjugate can be often found in efficient bulk‐heterojunction (BHJ) solar cells.^14^

While diacceptor *anti–anti* dithieno[1,4]thiazine **3 a‐aa** was synthesized with an excellent yield of 83 %, that is, an average yield of 95 % per bond forming, the corresponding *syn–syn* dithieno[1,4]thiazine **3 b‐ss** was obtained in a moderate yield of 40 %. Initiated by bromine‐lithium exchange the LiForK synthesis of a corresponding diacceptor phenothiazine **6** gives a good yield of 74 %. The required brominated phenothiazine **5** was synthesized as indicated in the literature.^13^

**Scheme 4:**
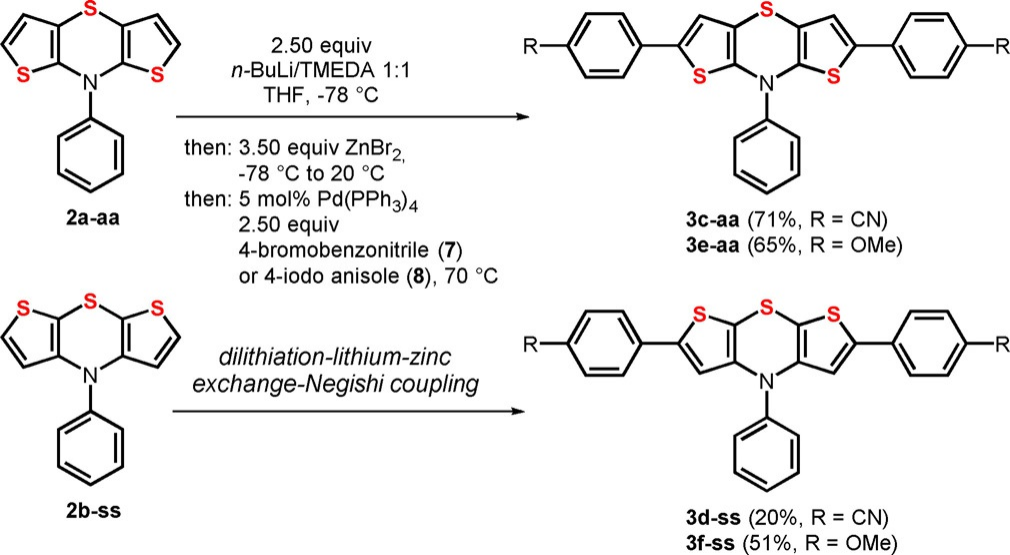
One‐pot synthesis of 2,6‐diarylated *anti–anti* and *syn–syn* dithieno[1,4]thiazines **3** by a consecutive (pseudo)three‐component dilithiation‐lithium‐zinc exchange‐Negishi coupling (exemplarily illustrated for **3 c**).

For efficient twofold α‐arylation of *N*‐phenyl dithieno[1,4]thiazines we envisioned a (pseudo)three component dilithiation‐lithium‐zinc exchange‐Negishi coupling, which was developed for *syn–syn* dithieno[1,4]thiazines like **2 b‐ss**.^10^ This sequence was employed for synthesizing four additional 2,6‐diarylated dithieno[1,4]thiazines **3** in moderate to good yields (20–71 %) of both *anti–anti* and the *syn–syn* isomers with diacceptor (**3 c‐aa** and **3 d‐ss**) and bisdonor (**3 e‐aa** and **3 f‐ss**) substitution pattern (Scheme 4).

### Electronic Properties and Structures

With respect to potential applications of functionalized dithieno[1,4]thiazines in organic electronics the electronic properties and structures of the dithieno[1,4]thiazines **3** were assessed experimentally and computationally. The electronic ground states were examined by cyclic voltammetry experiments and quantum chemical calculations on DFT level of theory. The excited states were examined by absorption and emission spectroscopy in dichloromethane solutions and by TDDFT calculations. All ground and excited state (S_1_) geometries were optimized using the Gaussian09 program package,^15^ the PBE1PBE functional^16^ and the 6‐31G** basis set^17^ and were confirmed as minima by analytical frequency analyses. The excitation energies were calculated with TDDFT^18^ methods implemented in the Gaussian09 program package using the PBE1PBE functional^16^ and the 6‐31G** or the 6–31+G** basis set^17^ as indicated. The polarizable continuum model (PCM) was always applied for the calculations with the same solvent used in the corresponding experiment.^19^

**Figure 1:**
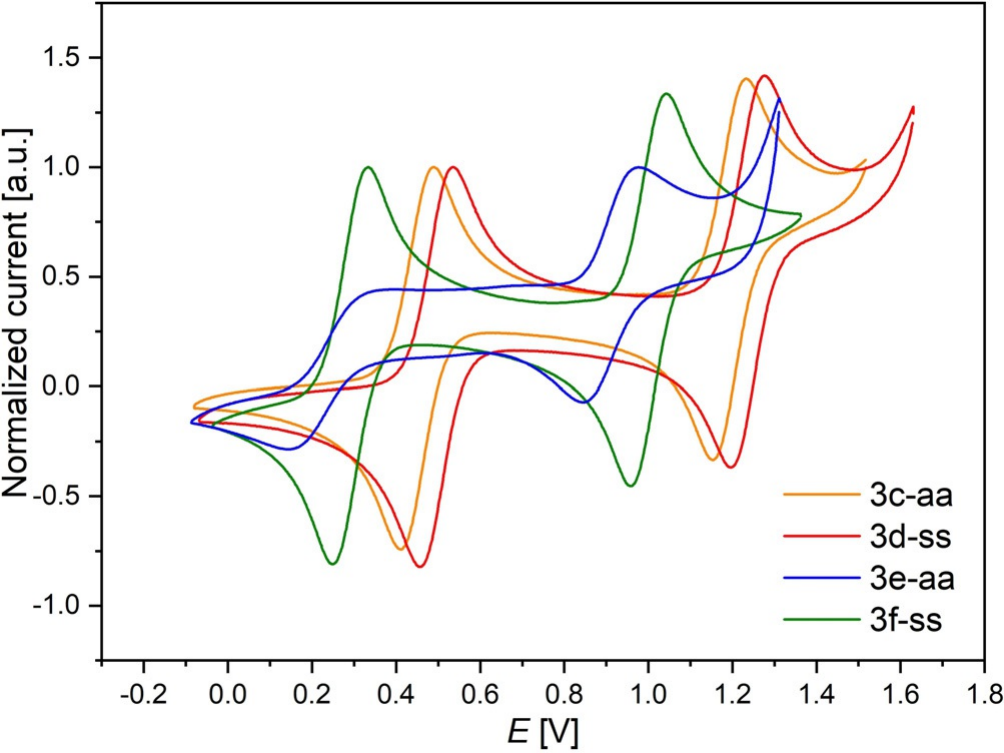
Selected cyclic voltammograms of the 2,6‐disubstituted dithieno[1,4]thiazines **3 c‐aa**–**3 f‐ss** (CH_2_Cl_2_, *T=*298 K, 0.1 m [Bu_4_N][PF_6_], *v*=100 mV s^−1^, Pt‐working, Ag/AgCl‐reference and Pt‐counter electrode, [Cp*Fe]/[Cp*Fc]^+^ as an internal standard; Cp*Fe=decamethylferrocene, *E*_*0/+1*_=−95 mV vs. ferrocene with *E*_*0/+1*_(Fc/Fc^+^)=450 mV).^20^.

All dithieno[1,4]thiazines exhibit reversible oxidations in dichloromethane solutions. By different functionalization the oxidation potentials of mono‐ and dioxidations of the *anti–anti* dithieno[1,4]thiazines (**3 a‐aa**, **3 c‐aa** and **3 e‐aa**) (Figure 1, Table 1) can be tuned over a broad range (Δ*E*_*0/+1*_(**3 a‐aa**, **3 e‐aa**)=670 mV, Δ*E*_*+1/+2*_(**3 a‐aa**,**3 e‐aa**)=610 mV). On one hand, the redox potentials can be shifted anodically up to 540 mV (230 mV for *E*_*+1/+2*_) and on the other hand they can be shifted cathodically by 130 mV (380 mV for *E*_*+1/+2*_) against the unfunctionalized system **2 a** (*E*_*0/+1*_(**2 a‐aa**)=374 mV, *E*_*+1/+2*_(**2 a‐aa**)=1292 mV^9^) by introducing strong acceptors or donors, respectively. This also holds true for *syn–syn* isomers (**3 b‐aa**, **3 d‐aa** and **3 f‐aa**), but the measured range of redox potentials is narrower (Δ*E*_*0/+1*_(**3 b‐ss**,**3 f‐ss**)=600 mV).

**Table 1:** Electrochemical properties and HOMO‐energy levels *E_HOMO_* of compounds **3** and **6**.

Compound	*E*_*0/+1*_ [mV]^[a]^	*E*_*+1/+2*_ [mV]^[b]^	*K_SEM_* ^[a]^	*E_HOMO_* [eV] ^[c]^
**3 a‐aa**	915	1522	1.95×10^10^	−5.594
**3 b‐ss**	893	1552	1.46×10^11^	−5.622
**6**	1118	–	–	−5.923
**3 c‐aa**	453	1202	4.95×10^12^	−5.133
**3 d‐ss**	491	1247	6.51×10^12^	−5.232
**3 e‐aa**	247	916	2.18×10^11^	−4.829
**3 f‐ss**	291	1000	1.01×10^12^	−4.914

[a] Recorded in CH_2_Cl_2_, *T=*298 K, 0.1 m [Bu_4_N][PF_6_], *v*=100 mV s^−1^, Pt‐working, Ag/AgCl‐reference and Pt‐counter electrode, [Cp*Fe]/[Cp*Fe]^+^ as an internal standard (Cp*Fe=decamethylferrocene, *E*_*0/+1*_=−95 mV vs. ferrocene with *E*_*0/+1*_(Fc/Fc^+^)=450 mV).^20^ [b] $K_{SEM}=10^{\frac{E_{+1/+2\;}-{\;E}_{0/+1}}{59\;mV}}$ . [c] PBE1PBE/6‐31G**, PCM CH_2_Cl_2_.

**Figure 2:**
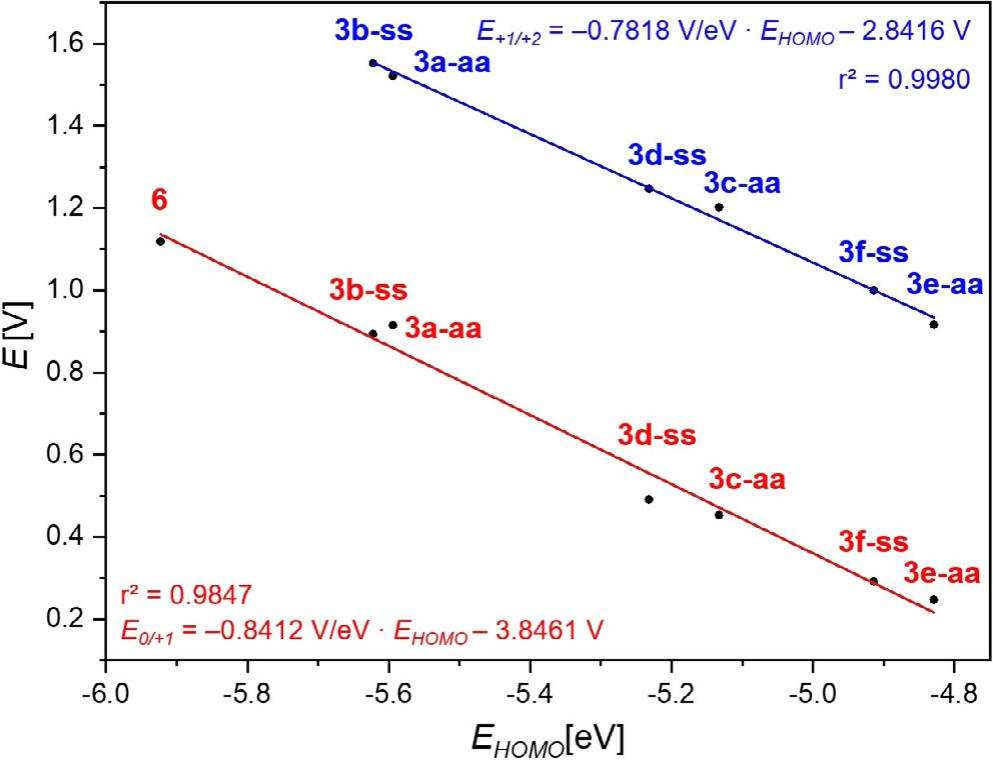
Correlation of the oxidation potentials (0.1 m [Bu_4_N][PF_6_], *v*=100 mV s^−1^, Pt‐working, Ag/AgCl‐reference and Pt‐counter electrode, [Cp*Fe]/[Cp*Fe]^+^ as an internal standard; Cp*Fe=decamethylferrocene) of the monooxidation *E*_*0/+1*_ (red) and the dioxidation *E*_*+1/+2*_ (blue) with the HOMO energies *E_HOMO_* of the compounds **3** and **6** (PBE1PBE/6‐31G** PCM CH_2_Cl_2_).

Phenothiazine **6**, the heterocyclic topological analogue of **3 a‐aa** and **3 b‐ss**, is about 200 mV anodically shifted against **3 a‐aa** and **3 b‐ss**, which is in line with the unfunctionalized systems^9^ and the calculated HOMO energy levels (Figure 2). The HOMO energy levels scale with the electron richness and the ionization potential of a compound. Therefore, the oxidation potentials give a good linear correlation (r^2^=0.9847 for *E*_*0/+1*_, r^2^=0.9980 for *E*_*1/+2*_) with the HOMO energy levels, underlining the continuous impact of the substituents on the electron richness and thus on the redox potentials.

However, there is one exception: *anti–anti* dithieno[1,4]thiazines have been shown to be more electron‐rich than the regioisomeric *syn–syn* dithieno[1,4]thiazines.^9^ But whereas the oxidation potentials of both *anti–anti* diaryl dithieno[1,4]thiazines (**3 c‐aa** and **3 e‐aa**) are shifted about 40 mV cathodically against the corresponding *syn–syn* isomers (**3 d‐ss** and **3 f‐ss**) as expected, surprisingly, a reverse behavior can be observed comparing **3 a**–**aa** and **3 b‐ss** (Δ*E*_*0/+1*_(**3 b‐ss**–**3 a‐aa**)=−22 mV) bearing strong acceptors. Nevertheless, the hierarchy of the oxidation potentials, especially of **3 a‐aa** and **3 b‐ss**, can be correctly reproduced by DFT‐calculated oxidation potentials (see Supporting Information, Section 6). Further studies concerning the rotational barriers of dithieno[1,4]thiazine‐substituent bonds Δ*G* ^*≠*^_*rot*_ (Table 2) imply that the strength of the dithieno[1,4]thiazine‐substituent interaction is larger in the *anti–anti* isomers compared to the *syn–syn* isomers. All DFT‐computed rotational barriers of the *anti–anti* isomers are higher than the barriers of the *syn–syn* isomers, so that a stronger π‐bonding character can be plausibly assumed.

**Table 2:** Differences of the calculated rotational barriers of the dithieno[1,4]thiazine‐substituent bonds between the *anti–anti* and *syn–syn* isomers ΔΔ*G* ^*≠*^_*rot*_ (PBE1PBE/6‐31G** PCM CH_2_Cl_2_).

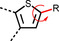	
Compared isomers	ΔΔ*G* ^*≠*^_*rot*._[kcal/mol]
**3 a‐aa**–**3 b‐ss**	1.061
**3 c‐aa**–**3 d‐ss**	0.553
**3 e‐aa**–**3 f‐ss**	0.201

Consequently, the offbeat oxidation potential of **3 a‐aa** together with the larger substituent effects on the oxidation potentials of the *anti–anti* isomers can be rationalized (Table 1). This is also in line with the higher tendency towards delocalization of the *anti–anti* dithieno[1,4]thiazine mother system **2 a‐aa**.^9^ Additionally, the radical cations showed unexpected relative stabilities. The stabilities of radical cations can be compared by their semiquinone formation constants *K_SEM_* (equilibrium constants of the comproportionation of dications and neutral ground states).^21^ As indicated by *K_SEM_*, the radical cations formed upon oxidation of the *anti–anti* isomers, in contrast to the unsubstituted systems,^9^ are always one order of magnitude lower than those of the corresponding *syn–syn* isomers (Table 1). This can well be a consequence of stronger substituent effects in the *anti–anti* isomers resulting in a higher destabilization of the radical cations by the acceptors in **3 a‐aa** and **3 c‐ss** and a higher stabilization of the dication in **3 e‐aa** (Δ*E*_*+1/+2*_(**3 f‐ss**–**3 e‐aa**)=84 mV) relative to the *syn–syn* isomers, respectively. Moreover, the *K_SEM_* values of all substituted dithieno[1,4]thiazines **3** are several magnitudes lower than the *K_SEM_* values of the unsubstituted mother systems **2**.^8^, ^9^ However, in comparison to other organic redox systems^21^ *K_SEM_*≈10^10^–10^12^ are still remarkably high.

**Figure 3:**
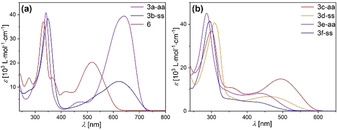
UV–Vis absorption spectra of (a) dithieno[1,4]thiazines **3 a‐aa** and **3 b‐ss** and phenothiazine **6** and (b) dithieno[1,4]thiazines **3 c‐aa**–**3 f‐ss** (*c*(**3**,**6**)=10^−5^ m, CH_2_Cl_2_, *T=*298 K).

Similar to the electrochemical properties, the photophysical properties of the dithieno[1,4]thiazine isomers **3** (Table 3, Figure 3) can be tuned over a broad spectrum of the UV/Vis by functionalization as well. The longest wavelength absorption maxima *λ*_max,abs_ of the *anti–anti* isomers range from *λ*_max,abs_(**3 e‐aa**)=436 nm to *λ*_max,abs_(**3 a‐aa**)=641 nm, equaling an energy difference of 0.91 eV. An equally wide range of 0.96 eV is exhibited by the *syn–syn* isomers. Nevertheless, the *λ*_max,abs_ of *syn–syn* isomers are always hypsochromically shifted compared to the corresponding *anti–anti* isomers. And in addition, the longest wavelength absorption bands of all *anti–anti* isomers are significantly more intense compared to the *syn–syn* isomers. For example, the extinction coefficient *ϵ* at the longest wavelength absorption maximum of **3 a‐aa** (*ϵ(λ*_max,abs_ *)=*39 400 L mol^−1^ cm^−1^) exceeds that of **3 b‐ss** (*ϵ(λ*_max,abs_ *)=*12 350 L mol^−1^ cm^−1^) by more than three times. This accounts for a more efficiently conjugated push‐pull system in **3 a‐aa** than in **3 b‐ss**, which again is in line with the stronger dithieno[1,4]thiazine‐substituent interaction in the *anti–anti* isomers (Table 2).

**Table 3:** Photophysical properties of compounds **3** and **6**.

Compound	*λ*_max,abs._[nm]^[a]^	*ϵ*(*λ*_max,abs_) [L mol^−1^ cm^−1^]	*∫ϵ(λ)dλ* [10^13^ L mol^−1^]	*λ*_max,em_ [nm]^[b]^	*E*_*0‐0*_ [eV]^[c]^	Δ$\tilde{\nu }$ [cm^−1^]^[d]^	*Φ_F_* ^[e]^
**3 a‐aa**	641	39 400	7.12	719	1.79	1700	0.52^[f]^
	472	3300					
	342	41 200					
**3 b‐ss**	623	12 350	4.56	750	1.76	2700	0.01^[f]^
	349	35 700					
**6**	517	20 300	5.17	639	2.09	3700	0.57^[g]^
	418	8000					
	366	15 150					
	331	37 900					
	277	13 960					
**3 c‐aa**	496	14 800	4.82	611	2.17	3800	0.29^[g]^
	300	33 740					
**3 d‐ss**	459	6700	3.57	620	2.23	5700	0.03^[g]^
	309	38 900					
**3 e‐aa**	436	8200	3.69	559	2.39	5000	0.02^[h]^
	287	42 800					
**3 f‐ss**	420	4300	2.84	576	2.48	6400	<0.01^[h]^
	295	31 800

[a] Recorded in CH_2_Cl_2_ at *T=*298 K, c(**3**,**6**)=10^−5^ m. [b] Recorded in CH_2_Cl_2_ at *T=*298 K, *c*(**3**,**6**)=10^−6^  M. [c] *E*_*0‐0*_ was determined from the crossing point of absorption and emission spectra. [d] Δ$\tilde{\nu }$ =1/*λ*_max,abs_ −1/*λ*_max,em_. [e] *Φ_F_* in CH_2_Cl_2_ at *T=*298 K was determined relative to a fluorescence standard. [f] Nile Blue A perchlorate in methanol as a standard (*λ_exc_*=626 nm, *Φ_F_*=0.21^22^). [g] DCM in methanol as a standard (*λ_exc_*=492 nm, *Φ_F_*=0.43^23^). [h] Coumarin 153 in ethanol as a standard (*λ_exc_*=422 nm, *Φ_F_*=0.38^24^).

**Figure 4:**
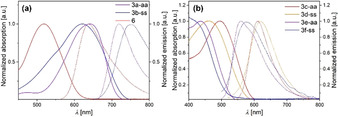
Normalized UV–Vis absorption (solid lines) and emission spectra (dashed lines) of (a) dithieno[1,4]thiazines **3 a‐aa** and **3 b‐ss** and phenothiazine **6** and (b) dithieno[1,4]thiazines **3 c**–**3 f** (recorded in CH_2_Cl_2_ at *T=*298 K, *c*(**3**,**6**)=10^−6^ m, *λ_exc_*(**3**,**6**)=*λ*_*max,abs*_(**3**,**6**)).

Furthermore, the spectral integrals *∫ϵ(λ)dλ* (Table 3) highlight that the overall absorption of the *anti–anti* isomers is significantly intensified. In contrast the analogous phenothiazine **6** exhibits a way more hypsochromically shifted absorption maximum of *λ*_max,abs_=517 nm and almost a halved *ϵ* of the longest wavelength absorption maximum in comparison to **3 a‐aa**. The favorable impact of the benzo‐thieno exchange leading from phenothiazines to dithieno[1,4]thiazines^8^, ^9^ is thereby clearly emphasized. DFT calculations on the UV/Vis absorption spectra (Table 4) correctly reproduce the experimental data and indicate that characteristic longest wavelength absorption maxima mostly originate from HOMO–LUMO transitions. All dithieno[1,4]‐thiazines **3** fluoresce in dichloromethane solutions (Table 3, Figure 4), but the Stokes shifts Δ$\tilde{\nu }$ differ quite noticeably. The Stokes shifts are about 2000 cm^−1^ for the dithieno[1,4]thiazines **3 a‐aa** and **3 b‐ss** bearing strongly electron withdrawing groups, whereas they are in a range of 5000–6000 cm^−1^ for the donor substituted dithieno[1,4]thiazines **3 e‐aa** and **3 f‐ss**.

**Table 4:** TDDFT calculations on the UV/Vis‐absorption maxima of the compounds **3** and **6** (PBE1PBE/6‐31G**, PCM CH_2_Cl_2_).

Compound	*λ*_max,exp_ [nm] (*ϵ* [L mol^−1^ cm^−1^])^[a]^	*λ*_max,calcd_ [nm]	Oscillator strength	Most dominant contributions
**3 a‐aa** ^[b]^	641 (39 400)	625	0.9395	HOMO→LUMO (99 %)
	472 (3300)	477	0.0504	HOMO→LUMO+1 (97 %)
	342 (41 200)	356	0.3642	HOMO−2→LUMO (97 %)
		342	0.1856	HOMO−1→LUMO (97 %)
**3 b‐ss**	632 (12 350)	636	0.4468	HOMO→LUMO (99 %)
	349 (35 700)	350	0.5038	HOMO−2→LUMO (96 %)
**6**	517 (20 300)	535	0.6349	HOMO→LUMO (98 %)
	418 (8000)	421	0.0706	HOMO→LUMO+1 (96 %)
	366 (15 150)	363	0.7812	HOMO−1→LUMO (95 %)
**3 c‐aa**	496 (14 800)	502	0.8492	HOMO→LUMO (97 %)
	300 (33 740)	299	0.9124	HOMO→LUMO+4 (82 %)
**3 d‐ss**	459 (6700)	482	0.3337	HOMO→LUMO (97 %)
	309 (38 900)	311	1.2624	HOMO−2→LUMO (85 %)
**3 e‐aa** ^[b]^	436 (8200)	436	0.5106	HOMO→LUMO (96 %)
	287 (42 800)	284	0.9076	HOMO−2→LUMO (49 %)
				HOMO−1→LUMO+1 (46 %)
**3 f‐ss** ^[b]^	420 (4300)	433	0.1836	HOMO→LUMO (96 %)
	295 (31 800)	297	1.2881	HOMO−1→LUMO+1 (73 %)

[a] Recorded in CH_2_Cl_2_, *c*(**3**,**4**)=10^−5^ m, *T=*293 K. [b] PBE1PBE/6–31+G**, PCM CH_2_Cl_2_ was used instead.

**Figure 5:**
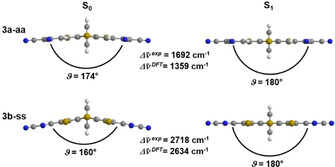
Optimized ground‐state (S_0_) and excited state (S_1_) geometries, S,*N*‐folding angles *ϑ* and stokes shifts Δ$\tilde{\nu }$ of **3 a‐aa** and **3 b‐ss** (PBE1PBE/6‐31G**, PCM CH_2_Cl_2_).

**Figure 6:**
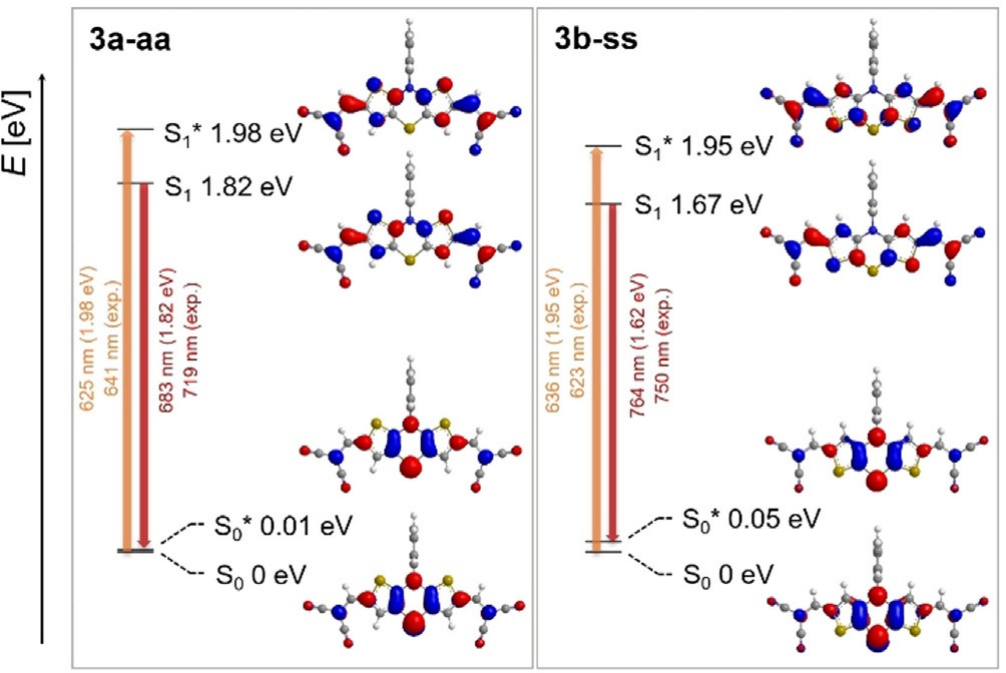
DFT‐computed Jablonski diagrams and Kohn–Sham FMOs corresponding to the S_0_‐S_1_*‐transition (longest wavelength absorption) and the S_1_‐S_0_*‐transition (fluorescence) of **3 a‐aa** and **3 b‐ss** (PBE1PBE/6‐31G**, PCM CH_2_Cl_2_, isosurface value at 0.04 a.u.).

DFT calculations on the ground and excited states of the dithieno[1,4]thiazines **3** and phenothiazine **6** reveal that the Stokes shifts can be explained with geometry changes accompanied by a planarization after photoexcitation (Figures 5 and Figure 6), in analogy to phenothiazines.^25^ The ground state geometries of all functionalized dithieno[1,4]thiazines **3** are folded along the S,*N*‐axis as a consequence of an unfavored Hückel antiaromaticity of a planarized thiazine and, thus, they possess a phenothiazine‐like^26^ butterfly structure. Interestingly, the Stokes shifts are at least 1000 cm^−1^ larger for the *syn–syn* isomers than for the *anti–anti* isomers.

**Figure 7:**
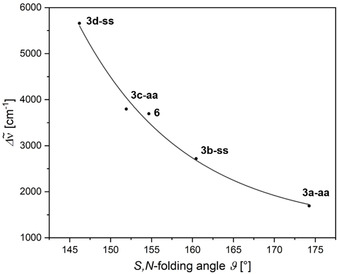
Dependence of the experimental Stokes shifts Δ$\tilde{\nu }$ of the DFT‐computed S,*N*‐folding angle *ϑ* of the diacceptor phenothiazine **6** and the diacceptor dithieno[1,4]thiazines **3 a‐aa**–**3 d‐ss** (PBE1PBE/6‐31G** PCM CH_2_Cl_2_). For illustration, we added an exponential fit (r^2^=0.9881).

Accordingly, the S,*N*‐folding angles *ϑ* are larger in *anti–anti* than in corresponding *syn–syn* isomers, respectively. In addition, *ϑ* increases concomitantly with the electron withdrawing character of the substituents implying a tunable antiaromatic character of the thiazine rings. The more electron‐rich the thiazine ring becomes the more folded it will be. This rationalizes the hierarchy of the Stokes shifts of compounds **3 a‐aa–3 d‐ss** and **6** (Figure 7). For example, the remarkably almost planar ground state geometry of **3 a‐aa** (*ϑ*=174°) is in accordance with the implemented strongly electron withdrawing acceptor substituents. The weaker substituent interactions in the corresponding *syn–syn* isomer **3 b‐ss** (*ϑ*=160°) explain the larger Stokes shift of **3 b‐ss** due to a higher thiazine located electron density. Otherwise, we did not observe a significant effect of donor substituents on *ϑ* (*ϑ*(**3 e‐aa**,**3 f‐ss**)=145°) compared to the 2,6‐unsubstituted dithieno[1,4]thiazines (*ϑ*(**2 a‐aa**,**2 b‐ss**)=144°)^8^, which indicates that the stabilization of the thiazine by folding predominates and delocalization is less favored.

Regarding the fluorescence quantum yields *Φ_F_* in dichloromethane solutions there are also striking differences between the isomers (Table 3). *Anti–anti* diacceptor‐dithieno[1,4]thiazines **3 a‐aa** (*Φ_F_*=0.52) and **3 c** (*Φ_F_*=0.29) fluoresce intensely, whereas their *syn–syn* regioisomers **3 b‐ss** (*Φ_F_*=0.01) and **3 d‐ss** (*Φ_F_*=0.03) fluoresce only weakly. The same trend holds true for the donor substituted dithieno[1,4]thiazines **3 e‐aa** (*Φ_F_*=0.02) and **3 f‐ss**, (*Φ_F_*<0.01) with their much weaker luminescence. In terms of *Φ_F_ anti–anti* dithieno[1,4]thiazine **3 b‐ss** represents a direct analogue to phenothiazine **6** (*Φ_F_*=0.57). In general, *Φ_F_* depends on the ratio of the radiative (*k_R_*) and the sum over all radiative and non‐radiative rate constants (*Σ k_NR_*) of the excited‐state decay (Equation (1)). The larger *k_R_* and the smaller *Σ k_NR_* become, the more *Φ_F_* will increase.

(1) $$\Phi_{F}=\frac{k_{R}}{k_{R}+\sum k_{NR}}\;\;\mathrm{with}\;\;\sum k_{NR}\approx k_{IC}+k_{ISC}$$

For the *anti–anti* dithieno[1,4]thiazines **3 a‐aa**, **3 c‐aa** and **3 e‐aa** the extinction coefficients *ϵ* as well as the TDDFT‐computed oscillator strengths *f* of the *S_0_‐S_1_* transitions are larger than for the corresponding *syn–syn* isomers, respectively (Table 4), suggesting that *k_R_* is larger as well. Furthermore, fluorescence lifetime measurements and TDDFT calculations on *k_R_* of **3 a‐aa** and **3 b‐ss** support this assumption and reveal that there is also a large difference in *Σ k_NR_* (Table 5): *Σ k_NR_*(**3 b‐ss**) is about 30‐times higher than *Σ k_NR_*(**3 a‐aa**). As the rate constants of the internal conversion (*k_IC_*) can be assumed to be approximately similar for both dithieno[1,4]thiazine regioisomers due to their similarly rigid structures and *S_0_‐S_1_* energy gaps (for *E*_*0‐0*_, see Table 3), the major non‐radiative decay could arise from different intersystem crossing rates (*k_ISC_*). The LUMO coefficient densities located on the sulfur atoms, representing their electronic participation in the *S_1_*‐state, are significantly smaller in each *anti–anti* isomer on the thiazine sulfur atoms and relatively similar on the thiophene sulfur atoms of pairs of isomers (Table 6). Since the comparatively high heavy‐atom effect of sulfur can be assumed to cause an enhanced intersystem crossing, a larger *k_ISC_* of the *syn–syn* isomers, along with smaller *k_R_* values, which, in turn, can plausibly rationalize the observed weaker fluorescence of all *syn–syn* isomers compared to the corresponding *anti–anti* isomers.

**Table 5:** Fluorescence lifetime *τ*, radiative rate constant *k_R_*, TDDFT‐computed rate constant *k_R_* ^*DFT*^ and resulting non‐radiative rate constant *Σ k_NR_* of **3 a‐aa** and **3 b‐ss**.

Compound	*τ* [ns]	*k_R_* [s^−1^]^[a]^	*k_R_* ^*DFT*^ [s^−1^]^[b,c]^	Σ *k_NR_* [s^−1^]^[d]^
**3 a‐aa**	2.48	2.10×10^8^	1.26×10^8^	1.49×10^8^
**3 b‐ss**	0.21	4.70×10^7^	5.49×10^7^	4.66×10^9^

[a] *k_R_*=*Φ_F_/τ*. [b] *k_R_* ^*DFT*^=2/3 *f_em_* $\tilde{\nu }$ *_em_* ^2^.^27^ [c PBE1PBE/6‐31G** PCM CH_2_Cl_2_. [d] Σ *k_NR_*=(1‐*Φ_F_*)*/τ*.

**Table 6:** LUMO coefficients localized on the thiazine‐ and the two thiophene‐sulfur atoms *S_LUMO_* 28 (PBE1PBE/6‐31G** PCM CH_2_Cl_2_).

Compound	*S_LUMO_* ^*Thiazine*^ [%]	*S_LUMO_* ^*Thiophene*^ [%]
**3 a‐aa**	0.05	11.20
**3 b‐ss**	0.65	10.11
**6**	0.31	–
**3 c‐aa**	0.32	8.58
**3 d‐ss**	0.81	8.47
**3 e‐aa**	1.62	14.97
**3 f‐ss**	2.11	12.16

**Figure 8:**
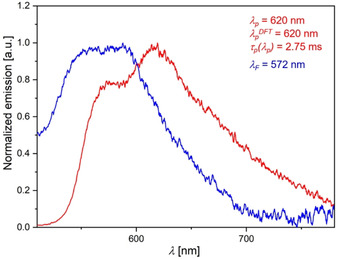
Phosphorescence (red, *λ_p_*), phosphorescence lifetime *τ_p_*, fluorescence (blue, *λ_F_*) of **3 f‐ss** (recorded in toluene, *λ_exc_*=420 nm, c(**3 f‐ss**)=10^−6^ m, *T=*77 K, degassed with N_2_) and TDDFT‐calculated phosphorescence *λ_p_* ^*DFT*^ (PBE1PBE/6‐31G** PCM toluene).

Increased intersystem crossing rates of *syn–syn* isomers are also supported by the phosphorescence of **3 f‐ss** in degassed toluene solutions at 77 K (Figure 8) and the absence of any detectable phosphorescence of **3 e‐aa** under the same conditions.

**Figure 9:**
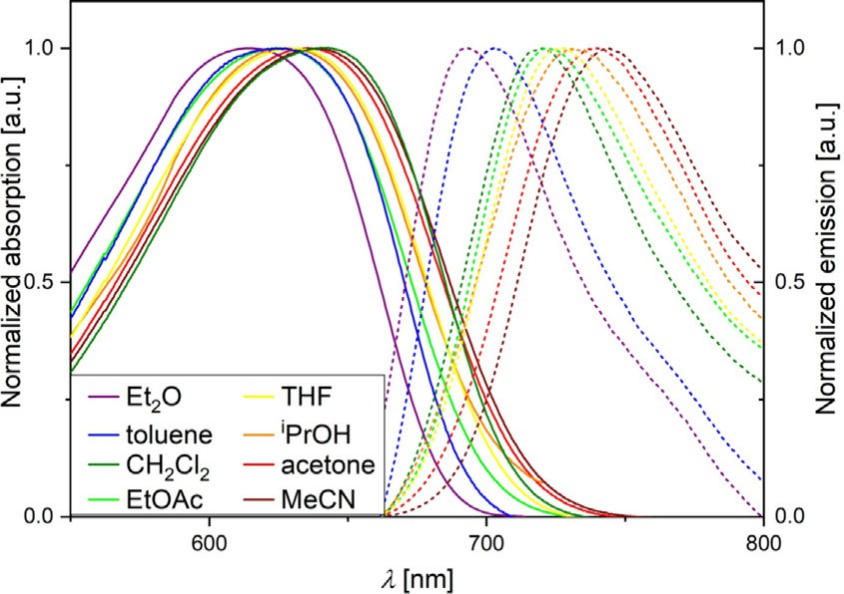
Solvatochromism study on a selected dithieno[1,4]thiazine—Normalized absorption (solid lines) and emission spectra (dashed lines) of **3 a‐aa** recorded in diethyl ether, toluene, dichloromethane, ethyl acetate, THF, isopropanol, acetone and acetonitrile (*c*(**3 a‐aa**)=10^−6^ m, *t=* 298 k, *λ_exc_*(**3 a‐aa**)=641 nm).

The acceptor substituted dithieno[1,4]thiazines **3 a‐aa**–**3 d‐ss** show positive emission solvatochromism as their emission maxima are shifted more bathochromically with increasing solvent polarity (Figure 9). Hence, the dipole moments increase upon photo excitation and the transitions corresponding to the longest wavelength absorption maxima possess charge transfer (CT) character. Using Lippert–Mataga plots (see Supporting Information, Section 5.1) the ground to excited state dipole moment changes Δ*μ(S_0_→S_1_)* were obtained (Table 7).

**Table 7:** Ground to excited state dipole moment change Δ*μ(S_0_→S_1_)* and FMO centroid distance *d_FMO_* (PBE1PBE/6‐31G**) of compounds **3** and **6**.

Compound	Δ*μ(S_0_→S_1_)* [D]	*d_FMO_* [Å]^28^
**3 a‐aa**	2.77	0.50
**3 b‐ss**	3.78	0.85
**6**	5.22	1.24
**3 c‐aa**	6.07	1.44
**3 d‐ss**	8.50	2.53

**Figure 10:**
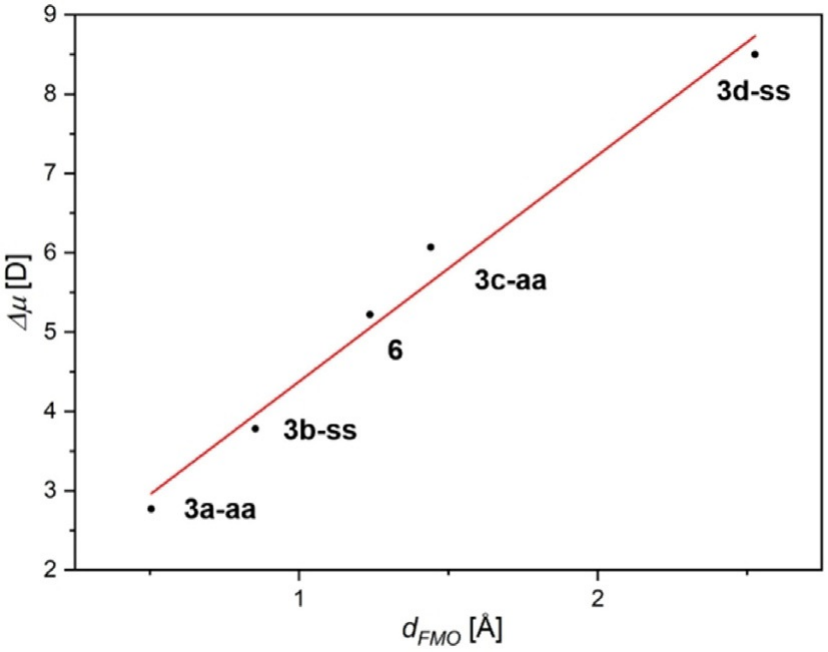
Ground to excited state dipole moment change *Δμ(S_0_→S_1_)* vs. FMO centroid distance *d_FMO_* of the compounds **3** and **6**.

Since the CT‐absorption bands are dominated by HOMO–LUMO transitions (Table 4), the different CT‐character of the isomers is illustrated by correlation of the centroid distances of these frontier molecular orbitals *d_FMO_* with Δ*μ(S_0_→S_1_)* (Figure 10, r^2^=0.9768).

**Figure 11:**
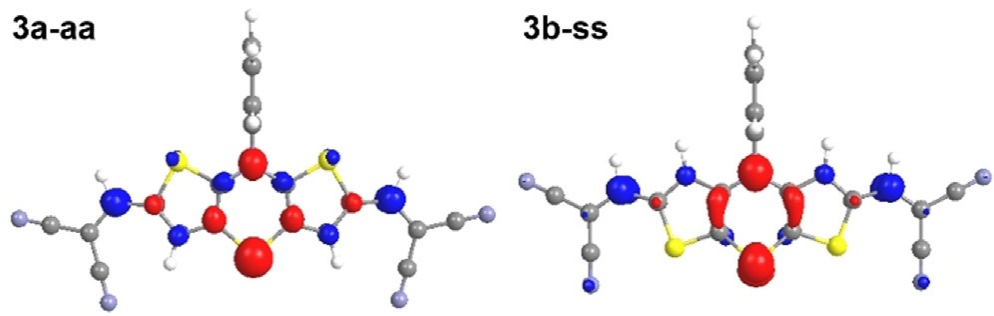
HOMO→LUMO difference plot of **3 a** and **3 b** (red=decrease, blue=increase, PBE1PBE/6‐31G**).

The change of dipole moment is larger for the *syn–syn* isomers **3 b‐ss** and **3 d‐ss** than for the corresponding *anti–anti* isomers **3 a‐aa** and **3 c‐aa**. Thus, the electronic dithieno[1,4]thiazine‐substituent transmission depending on the thiophene anellation mode becomes apparent again. For instance, the change of dipole moment of *syn–syn* **3 b‐ss** is around 1 D larger than of *anti–anti* **3 a**, whereas the difference **3 c‐aa** and **3 d‐ss** is even around 2 D (Table 7). This pronounced larger CT‐character of the *syn*–*syn* isomers is visualized by the difference plots of the Kohn–Sham FMOs suggesting a more clarified separation of the thiazine an acceptor *π*‐systems (Figure 11).

The larger Δ*μ(S_0_→S_1_)* of phenothiazine **6** in comparison to **3 a‐aa** and **3 b‐ss** points out that the electronic interaction with the acceptor substituents is weaker in phenothiazines than in dithieno[1,4]thiazines. Therefore, the benzo‐thieno exchange emphasizes the favorable impact of increased delocalization on the electronic properties, in particular, on charge transmission within the chromophores.

## Conclusions

A complementary series of six novel acceptor‐ and donor‐functionalized dithieno[1,4]thiazine regioisomers was efficiently accessed by employing straightforward one‐pot processes. In addition, the acceptor substituted phenothiazine **6** was also synthesized in a one‐pot fashion for electronic comparison.

The acceptor interaction in *anti–anti* isomers surpasses that in *syn–syn* isomers accompanied by a stronger intramolecular charge transfer, as supported by absorption and emission spectroscopy and calculations. For instance, the DFT calculated ground state geometry of the *anti–anti* dithieno[1,4]thiazine **3 a‐aa** is almost planar, but its *syn–syn* regioisomer **3 b‐ss** is folded noticeably due to a less efficient depopulation of the 8*π*‐electron containing thiazine ring. It is noteworthy that in phenothiazine **6** substituent interactions are smaller than in the corresponding dithieno[1,4]thiazines **3 a‐aa** and **3 b‐ss** according to solvatochromicity studies. As a consequence of the fact that dithieno[1,4]thiazines form much stronger push–pull systems than phenothiazines. Furthermore, the absorption of the relatively small chromophores **3 a‐aa** and **3 b‐ss** (MW=440 g mol^−1^) almost reaches the near infrared region. In comparison, more extended chromophores (*π*‐system with MW=660 g mol^−1^) applied in efficient bulk‐heterojunction solar cells based on dithieno pyrrole as a donor exhibit more hypsochromic absorption bands.^14^ Therefore, especially the very intensively absorbing **3 a‐aa** is a promising candidate for photovoltaics in the miniaturization of dye‐architectures.^6^, ^29^ Dithieno[1,4]thiazines can be considered as phenothiazine substitutes in donor‐acceptor conjugates for improving the properties while maintaining structural changes and molecular sizes small.

Finally, acceptor‐functionalized *anti–anti* dithieno[1,4]‐thiazines fluoresce very intensively in contrast to the *syn–syn* regioisomers, as a consequence of faster intersystem crossing in *syn–syn* isomers inter alia. Hence, in particular acceptor‐functionalized *anti–anti* dithieno[1,4]thiazines can be also considered as intense red‐light emitters (*Φ_F_*=0.52) or even as NIR‐emitters for biomedical imaging^30^ or OLED‐devices.^3^ Further studies on dithieno[1,4]thiazine based donor‐acceptor conjugates and their applications are currently underway.

## Experimental Section

Experimental details, full characterizations, ^1^H and ^13^C NMR spectra of compounds **3** and **6**, additional cyclic voltammograms, absorption and emission spectra, as well as all DFT computed XYZ‐coordinates, energies and absorption spectra are compiled in the Supporting Information.

Typical procedure for the preparation of 2,6‐diacceptor‐substituted dithieno[1,4]thiazine **3 a‐aa** via Lithiation‐Formylation‐Knoevenagel sequence (LiForK): In a flame‐dried Schlenk vessel with magnetic stir bar under nitrogen atmosphere 8‐phenyl‐8*H*‐dithieno[3,2‐*b*:2′,3′‐*e*][1,4]thiazine (**2 a‐aa**) (146 mg, 0.51 mmol) and tetramethylethylene‐diamine (0.19 mL, 1.28 mmol) were dissolved in dry THF (5.10 mL) and cooled down to −78 °C (isopropanol/dry ice). Then, *n‐*butyllithium (0.80 mL, 1.28 mmol, 1.6 m in hexane) was added dropwise slowly via syringe to the vigorously stirred solution. Stirring was continued at −78 °C for 2 h. Then dry DMF (118 μL, 1.53 mmol) was added, stirring was continued at −78 °C for another 90 min and then at ambient temperature for 30 min. To the reaction mixture acetic acid (0.15 mL, 2.55 mmol) was added. After stirring at ambient temperature for 15 min, malononitrile (**4**) (101 mg, 2.62 mmol) was added and the stirring was continued at ambient temperature for 20 min. The volatiles were removed by evaporation and the crude product was purified by flash column chromatography using gradient elution (*n*‐hexane/ethyl acetate 2:1→ethyl acetate) and suspension in ethanol giving compound **3 a‐aa** (185 mg, 83 %) as a dark blue powder, Mp 319–321 °C (decomposition). *R*_f_ (*n*‐hexane/ethyl acetate 2:1)=0.23. ^1^H NMR (600 MHz, [D_6_]DMSO, 372 K): *δ* 7.51 (s, 2 H), 7.69–7.75 (m, 3 H), 7.77–7.81 (m, 2 H), 8.10 ppm (s, 2 H). ^13^C NMR (150 MHz, [D_6_]DMSO, 372 K): *δ* 69.2 (C_quat_), 110.8 (C_quat_), 114.5 (C_quat_), 115.2 (C_quat_), 124.8 (C_quat_), 127.7 (CH), 131.6 (CH), 131.7 (CH), 132.0 (CH), 139.8 (C_quat_), 150.1 (CH), 152.2 ppm (C_quat_). MS(MALDI‐TOF) *m*/*z*: 438.980 ([M]^+^). IR: $\tilde{\nu }$ [cm^−1^]=2212 (w), 1561 (s), 1555 (s), 1501 (w), 1489 (w), 1423 (w), 1368 (s), 1325 (s), 1314 (s), 1287 (s), 1273 (s), 1254 (s), 1209 (s), 1169 (s), 1148 (s), 1121 (s), 1074 (m), 1057 (m), 1026 (m), 930 (m), 887 (m), 866 (m), 851 (m), 810 (m), 797 (m), 748 (m), 691 (s), 648 (m), 604 (s). Anal calcd for C_22_H_9_N_5_S_3_ (439.5): C 60.12, H 2.06, N 15.93, S 21.88; Found: C 59.89, H 1.92, N 15.73, S 22.08.

Typical procedure for the preparation of 2,6‐diarylsubstituted dithieno[1,4]thiazine **3 c‐aa** by dilithiation‐lithium‐zinc exchange‐Negishi coupling: In a flame‐dried Schlenk vessel with magnetic stir bar under nitrogen atmosphere 8*H*‐dithieno[3,2‐*b*:2′,3′‐*e*][1,4]thiazine (**2 a‐aa**) (206 mg, 0.72 mmol) and tetramethylethylenediamine (0.27 mL, 1.80 mmol) were dissolved in dry THF (7.20 mL) and cooled down to −78 °C (isopropanol/dry ice). Then, *n*‐butyllithium (1.13 mL, 1.80 mmol, 1.6 m in hexane) was added dropwise slowly via syringe to the vigorously stirred solution. Stirring was continued at −78 °C for 2 h, while zinc dibromide (486 mg, 2.16 mmol) was vacuum dried at 120 °C for 1.5 h. After cooling dry zinc dibromide to ambient temperature, dry THF (2.00 mL) was added. The resulting zinc dibromide solution was added dropwise into the reaction mixture, which was then stirred at −78 °C for 30 min. After the reaction mixture had slowly warmed up to ambient temperature, tetrakis(triphenylphosphane)palladium(0) (42 mg, 5 mol %) and 4‐bromobenzonitrile (**7**) (328 mg, 1.80 mmol) were added and the reaction solution was stirred at 70 °C for 1 h. The volatiles were removed by evaporation and the crude product was purified by flash column chromatography using gradient elution (*n*‐hexane/ethyl acetate 4:1 with 1 % triethyl amine→*n*‐hexane/ethyl acetate 1:1 with 1 % triethyl amine) and suspension in acetone giving compound **3 c‐aa** (249 mg, 71 %) as a violet powder, Mp 274–275 °C. *R*_f_ (*n*‐hexane/ethyl acetate 3:1)=0.36. ^1^H NMR (600 MHz, [D_6_]DMSO, 393 K): *δ* 6.23 (s, 2 H), 7.54 −7.57 (m, 1 H), 7.57–7.60 (m, 4 H), 7.62–7.66 (m, 2 H), 7.66–7.70 ppm (m, 6 H). ^13^C NMR (150 MHz, [D_6_]DMSO, 393 K): *δ* 108.3 (C_quat_), 108.6 (C_quat_), 117.8 (C_quat_), 122.9 (CH), 124.0 (CH), 126.7 (CH), 128.9 (CH), 130.1 (CH), 130.4 (C_quat_), 132.1 (CH), 136.7 (C_quat_), 142.2 (C_quat_), 142.7 ppm (C_quat_). MS(MALDI‐TOF) *m*/*z*: 489.115 ([M]^+^). IR: $\tilde{\nu }$ [cm^−1^] 3057 (w), 2990 (w), 2886 (w), 2218 (w), 1559 (m), 1493 (m), 1435 (s), 1408 (s), 1362 (w), 1296 (w), 1283 (w), 1271 (w), 1227 (w), 1177 (m), 1165 (m),1111 (w), 1045 (w), 1016 (w), 984 (w), 964 (w), 945 (w), 918 (w), 880 (w), 835 (w), 818 (s), 802 (m), 772 (w), 743 (w), 719 (w), 691 (m), 651 (w). Anal. calcd for C_28_H_15_N_3_S_3_ (489.6): C 68.69, H 3.09, N 8.58, S 19.64; Found: C 68.67, H 3.00, N 8.40, S 19.93.

## Conflict of interest

The authors declare no conflict of interest.

## Supporting information

As a service to our authors and readers, this journal provides supporting information supplied by the authors. Such materials are peer reviewed and may be re‐organized for online delivery, but are not copy‐edited or typeset. Technical support issues arising from supporting information (other than missing files) should be addressed to the authors.

Supplementary
