# Supplementary material for: Widely Electronically Tunable 2,6‐Disubstituted Dithieno[1,4]thiazines—Electron‐Rich Fluorophores Up to Intense NIR Emission
Source: Chemistry. 2020 Sep 3;26(57):12978–86. doi: 10.1002/chem.202000859 (PMC7589349; doi:10.1002/chem.202000859)
Supplement: Supplementary file 1 — Supplementary [file CHEM-26-12978-s001.pdf]

Chemistry–A European Journal

Supporting Information

Widely Electronically Tunable 2,6-Disubstituted Dithieno[1,4]thiazines—Electron-Rich Fluorophores Up to Intense NIR Emission

Lars May and Thomas J. J. Müller*[a]

Supporting Information

Table of Contents

1 General Considerations ... 1

2 Syntheses ... 2

2.1 General procedure 1 (GP1) for the synthesis of **3a-aa**, **3b-ss** and **6** via Lithiation-Formylation-Knoevenagel sequence (LiForK)[8] ... 2

2.1.1 2,2'-((8-Phenyl-8*H*-dithieno[3,2-*b*:2',3'-*e*][1,4]thiazine-2,6-diyl)bis(methanylylidene))dimalononitrile (**3a-aa**) ... 3

2.1.2 2,2'-((4-Phenyl-4*H*-dithieno[2,3-*b*:3',2'-*e*][1,4]thiazine-2,6-diyl)bis(methanylylidene))dimalononitrile (**3b-ss**) ... 4

2.1.3 2,2'-((10-Phenyl-10*H*-phenothiazine-3,7-diyl)bis(methanylylidene))dimalononitrile (**6**) ... 5

2.2 General procedure 2 (GP2) for the synthesis of **3c-aa** – **3f-ss** via dilithiation–lithium–zinc exchange–Negishi coupling[9] ... 6

2.2.1 4,4'-(8-Phenyl-8*H*-dithieno[3,2-*b*:2',3'-*e*][1,4]thiazine-2,6-diyl)dibenzonitrile (**3c-aa**) . 7

2.2.2 4,4'-(4-Phenyl-4*H*-dithieno[2,3-*b*:3',2'-*e*][1,4]thiazine-2,6-diyl)dibenzonitrile (**3d-ss**) . 7

2.2.3 2,6-Bis(4-methoxyphenyl)-8-phenyl-8*H*-dithieno[3,2-*b*:2',3'-*e*][1,4]thiazine (**3e-aa**) .. 8

2.2.4 2,6-Bis(4-methoxyphenyl)-4-phenyl-4*H*-dithieno[2,3-*b*:3',2'-*e*][1,4]thiazine (**3f-ss**) ... 9

3 ^{1}H and ^{13}C NMR spectra ... 10

3.1 2,2'-((8-Phenyl-8*H*-dithieno[3,2-*b*:2',3'-*e*][1,4]thiazine-2,6-diyl)bis(methanylylidene))dimalononitrile (**3a-aa**) ... 10

3.2 2,2'-((4-Phenyl-4*H*-dithieno[2,3-*b*:3',2'-*e*][1,4]thiazine-2,6-diyl)bis(methanylylidene))dimalononitrile (**3b-ss**) ... 11

3.3 2,2'-((10-Phenyl-10*H*-phenothiazine-3,7-diyl)bis(methaneylylidene))dimalononitrile (**6**) ... 12

3.4 4,4'-(8-Phenyl-8*H*-dithieno[3,2-*b*:2',3'-*e*][1,4]thiazine-2,6-diyl)dibenzonitrile (**3c-aa**) 13

3.5 4,4'-(4-Phenyl-4*H*-dithieno[2,3-b:3',2'-*e*][1,4]thiazine-2,6-diyl)dibenzonitrile (**3d-ss**) 14

3.6 2,6-Bis(4-methoxyphenyl)-8-phenyl-8*H*-dithieno[3,2-*b*:2',3'-*e*][1,4]thiazine (**3e-aa**) .15

3.7 2,6-Bis(4-methoxyphenyl)-4-phenyl-4*H*-dithieno[2,3-*b*:3',2'-*e*][1,4]thiazine (**3f-ss**) ..16

4 Cyclovoltammetric Data ... 17

5 Photophysical Properties of Compounds **3** and **6** ... 19

5.1 Solvatochromism Studies ... 19

5.2 Fluorescence Lifetimes of **3a-aa** and **3b-ss** and Phosphorescence Lifetime of **3f-ss** ... 25

6 Data of Quantum Chemical Calculations ... 26

6.1 Computed xyz-Coordinates, excitations of compounds **3** and **6** and selected properties derived from the DFT-calculations ... 27

6.1.1 Compound **3a-aa** ... 27

6.1.1.1 Computed xyz-Coordinates of compound **3a-aa** (PBE1PBE/6-31G** PCM CH_2Cl_2) 27

6.1.1.2 Computed Excitations of compound **3a-aa** (PBE1PBE/6-31+G** PCM CH_2Cl_2) ... 29

6.1.1.3 Computed xyz-Coordinates of S_1 of compound **3a-aa** (PBE1PBE/6-31G** PCM CH_2Cl_2) 30
6.1.1.4 Computed Excitations of S_1 (Emission of S_1) of compound **3a-aa** (PBE1PBE/6-31+G** PCM CH_2Cl_2) 32
6.1.1.5 Computed xyz-Coordinates of radical cation of compound **3a-aa** (uB3LYP/6-311G*) 32
6.1.1.6 Computed xyz-Coordinates of dication (S_0) of compound **3a-aa** (uB3LYP/6-311G*) 34
6.1.1.7 Reoptimization of compound **3a-aa** (uB3LYP/6-311G*) 35
6.1.1.8 Computed xyz-Coordinates of transition state of the acceptor rotation of compound **3a-aa** (PBE1PBE/6-31G** PCM CH_2Cl_2)....36
6.1.2 Compound **3b-ss**....38
6.1.2.1 Computed xyz-Coordinates of compound 3b (PBE1PBE/6-31G** PCM CH_2Cl_2).....38
6.1.2.2 Computed Excitations of compound **3b-ss** (PBE1PBE/6-31G** PCM CH_2Cl_2)........40
6.1.2.3 Computed xyz-Coordinates of S_1 of compound **3b-ss** (PBE1PBE/6-31G** PCM CH_2Cl_2) 41
6.1.2.4 Computed Excitations of S_1 (Emission of S_1) of compound **3b-ss** (PBE1PBE/6-31G** PCM CH_2Cl_2)....43
6.1.2.5 Computed xyz-Coordinates of radical cation of compound **3b-ss** (uB3LYP/6-311G*) 43
6.1.2.6 Computed xyz-Coordinates of dication (S_0) of compound **3b-ss** (uB3LYP/6-311G*) 45
6.1.2.7 Reoptimization of compound **3b-ss** (uB3LYP/6-311G*)....46
6.1.2.8 Computed xyz-Coordinates of transition state of the acceptor rotation of compound **3b-ss** (PBE1PBE/6-31G** PCM CH_2Cl_2) 47
6.1.3 Compound **6**....49
6.1.3.1 Computed xyz-Coordinates of compound **6** (PBE1PBE/6-31G** PCM CH_2Cl_2).......49
6.1.3.2 Computed Excitations of compound **6** (PBE1PBE/6-31G** PCM CH_2Cl_2) 51
6.1.3.3 Computed xyz-Coordinates of S_1 of compound **6** (PBE1PBE/6-31G** PCM CH_2Cl_2) 52
6.1.3.4 Computed Excitations of S_1 (Emission of S_1) of compound **6** (PBE1PBE/6-31G** PCM CH_2Cl_2) 54
6.1.3.5 Computed xyz-Coordinates of radical cation of compound **6** (uB3LYP/6-311G*) 54
6.1.3.6 Reoptimization of compound **6** (uB3LYP/6-311G*) 56
6.1.4 Compound **3c-aa**....57
6.1.4.1 Computed xyz-Coordinates of compound **3c-aa** (PBE1PBE/6-31G** PCM CH_2Cl_2)57
6.1.4.2 Computed Excitations of compound **3c-aa** (PBE1PBE/6-31G** PCM CH_2Cl_2) 59
6.1.4.3 Computed xyz-Coordinates of S_1 of compound **3c-aa** (PBE1PBE/6-31G** PCM CH_2Cl_2) 61
6.1.4.4 Computed Excitations of S_1 (Emission of S_1) of compound **3c-aa** (PBE1PBE/6-31G** PCM CH_2Cl_2)....63
6.1.4.5 Computed xyz-Coordinates of radical cation of compound **3c-aa** (uB3LYP/6-311G*) 63
6.1.4.6 Computed xyz-Coordinates of dication (S_0) of compound **3c-aa** (uB3LYP/6-311G*) 65

6.1.4.7 Reoptimization of compound **3c-aa** (uB3LYP/6-311G*) 67

6.1.4.8 Computed xyz-Coordinates of transition state of the benzonitrile rotation of compound **3c-aa** (PBE1PBE/6-31G** PCM CH_2Cl_2) 68

6.1.5 Compound **3d-ss** 70

6.1.5.1 Computed xyz-Coordinates of compound **3d-ss** (PBE1PBE/6-31G** PCM CH_2Cl_2) 70

6.1.5.2 Computed Excitations of compound **3d-ss** (PBE1PBE/6-31G** PCM CH_2Cl_2) 72

6.1.5.3 Computed xyz-Coordinates of radical cation of compound **3d-ss** (uB3LYP/6-311G*) 73

6.1.5.4 Computed xyz-Coordinates of dication (S_0) of compound **3d-ss** (uB3LYP/6-311G*) 76

6.1.5.5 Reoptimization of compound **3d-ss** (uB3LYP/6-311G*) 78

6.1.5.6 Computed xyz-Coordinates of transition state of the benzonitrile rotation of compound **3d-ss** (PBE1PBE/6-31G** PCM CH_2Cl_2) 79

6.1.6 Compound **3e-aa** 81

6.1.6.1 Computed xyz-Coordinates of compound **3e-aa** (PBE1PBE/6-31G** PCM CH_2Cl_2) 81

6.1.6.2 Computed Excitations of compound **3e-aa** (PBE1PBE/6-31+G** PCM CH_2Cl_2) 83

6.1.6.3 Computed xyz-Coordinates of radical cation of compound **3e-aa** (uB3LYP/6-311G*) 85

6.1.6.4 Computed xyz-Coordinates of dication (S_0) of compound **3e-aa** (uB3LYP/6-311G*) 87

6.1.6.5 Reoptimization of compound **3e-aa** (uB3LYP/6-311G*) 89

6.1.6.6 Computed xyz-Coordinates of transition state of the anisyl rotation of compound **3e-aa** (PBE1PBE/6-31G** PCM CH_2Cl_2) 90

6.1.7 Compound **3f-ss** 93

6.1.7.1 Computed xyz-Coordinates of compound **3f-ss** (PBE1PBE/6-31G** PCM CH_2Cl_2) 93

6.1.7.2 Computed Excitations of compound **3f-ss** (PBE1PBE/6-31+G** PCM CH_2Cl_2) 95

6.1.7.3 Computed xyz-Coordinates of T_1 of compound **3f-ss** (uPBE1PBE/6-31G** PCM toluene) 97

6.1.7.4 Computed Excitations of T_1 (Emission of T_1) of compound **3f-ss** (uPBE1PBE/6-31G** PCM toluene) 99

6.1.7.5 Computed xyz-Coordinates of radical cation of compound **3f-ss** (uB3LYP/6-311G*) 100

6.1.7.6 Computed xyz-Coordinates of dication (S_0) of compound **3f-ss** (uB3LYP/6-311G*) 102

6.1.7.7 Reoptimization of compound **3f-ss** (uB3LYP/6-311G*) 104

6.1.7.8 Computed xyz-Coordinates of transition state of the anisyl rotation of compound **3f-ss** (PBE1PBE/6-31G** PCM CH_2Cl_2) 105

6.2 DFT-Calculation of the redox potentials of compounds **3** and **6** 108

7 References 110

1 General Considerations

All reactions were carried out in flame-dried Schlenk tubes by using syringes under nitrogen atmosphere. Dry solvents for reactions and analytics were directly used from a MB-SPS 800 solvent drying system (MBraun) except of toluene, which was refluxed under nitrogen atmosphere over sodium, destilled and stored in a Schlenk flask over molecular sieve 4 Å under nitrogen atmosphere. 4-Phenyl-4*H*-dithieno[2,3-*b*:3',2'-*e*][1,4]thiazine (**2a-aa**),[1] 8-phenyl-8*H*-dithieno[3,2-*b*:2',3'-*e*][1,4]thiazine (**2b-ss**)[2] and 3,7-dibromo-10-phenyl-10*H*-phenothiazine (**5**)[3] were synthesized according to the literature procedures as indicated. Commercial grade reagents were purchased from Sigma Aldrich, Alfa Aesar, ABCR, Fluorochem and ACROS and used as supplied without further purification. Crude mixtures were adsorbed on Celite® 545 (0.02-0.20 mm) from Carl Roth GmbH Co.KG. The purification of products was performed on silica gel 60 M (0.04–0.063 mm) from Macherey–Nagel by using the flash technique under a pressure of 2 bar. For TLC silica gel coated aluminium plates (60, F_{254}) from Merck were employed and analyzed with UV light at 254 or 365 nm.

^{1}H, ^{13}C, and 135-DEPT NMR spectra were recorded at 293 K, 373 K or 393 K on 300 MHz (Bruker AVIII 300), 500 MHz (Bruker Avance DRX 500) or 600 MHz (Bruker Avance III 600) and the resonances of the residues of non-deuterated DMSO-d_6 (^{1}H δ = 2.50 ppm, ^{13}C δ = 39.52 ppm) was locked as internal standard. The multiplicities of signals are abbreviated as follows: s = singlet, d = doublet and m = multiplet. The assignments of C_{quat} and CH nuclei are based on DEPT spectra.

IR spectra were recorded on a Shimadzu IR Affinity-1 with ATR technique. The intensities of IR signals are abbreviated as s (strong), m (medium) and w (weak).

EI mass spectra were recorded on Triple-Quadrupole mass spectrometer TSQ 7000 (Finnigan MAT). MALDI-TOF mass spectra were measured on an UltrafleXtreme apparatus (Bruker Daltonics). High-resolution ESI mass spectra were recorded on a UHR-QTOF maXis 4G apparatus (Bruker Daltonics).

The elemental analyses were carried out on a Perkin Elmer Series II Analyser 2400 at the Institute for Pharmaceutical and Medicinal Chemistry at Heinrich-Heine-University Düsseldorf.

Melting points (uncorrected) were measured with a Büchi B545 apparatus.

Absorption spectra were recorded in dichloromethane high performance liquid chromatography (HPLC) grade at 293 K on Perkin Elmer UV/vis/NIR Lambda 19 spectrometer. For the determination of the extinction coefficients ε absorption measurements at five different concentrations were carried out. Emission spectra were recorded in dichloromethane HPLC grade at 293 K on a Perkin Elmer LS55 spectrometer. Emission spectra were recorded at

293 K on a LS55 spectrometer (Perkin Elmer). Phosphorescence spectra were recorded in toluene, degassed for at least 15 min with nitrogen, at 77 K (cooling with liquid nitrogen) using the phosphorescence mode of the LS55 spectrometer. Fluorescence quantum yields Φ_F were determined relative to a fluorescence standard (measurements at five concentrations). Nile Blue A perchlorate in MeOH (λ_{exc} = 626 nm, Φ_F = 0.21[4]) was used for compounds **3a-aa** and **3b-ss**, 4-(Dicyanomethylene)-2-methyl-6-(4-dimethylaminostyryl)-4*H*-pyran (DCM) in MeOH (λ_{exc} = 492 nm, Φ_F = 0.43[5]) was used for compounds **3c-aa**, **3d-ss** and **6** and Coumarine 153 in ethanol (λ_{exc} = 422 nm, Φ_F = 0.38[6]) was used for compounds **3e-aa** and **3f-ss**.

Quantum chemical calculations were carried out utilizing the HPC-Cluster Ivybridge of the Zentrum for Informations- und Medientechnologie (ZIM) at the Heinrich-Heine-University Düsseldorf.

Fluorescence lifetimes were measured on the FluoTime 300 (PicoQuant) using a picosecond white light laser (SuperK Extreme with SuperK Extend-UV, NKT Photonics).

Cyclic voltammetry experiments (EG&G Princeton Applied Research Model 263A potentiostat) were performed under argon atmosphere in dry and degassed dichloromethane at 293 K using *n*-Bu_4NPF_6 (0.1 M) as electrolyte and at scan rates v of 100, 250, 500 and 1000 mVs^{-1}. The three-electrode array consists of a working electrode with a 2 mm platinum disk, a platinum wire counter electrode, and an Ag/AgCl (3.0 M NaCl) reference electrode. The potentials were corrected by adding the internal standard decamethylferrocene to each measurement. Decamethylferrocene was referenced to the internal redox standard ferrocene ($E_0^{0/+1}$(decamethylferrocene) = -95 mV vs. $E_0^{0/+1}$(ferrocene) = 450 mV).[7] Therefore the outlined potentials are indirectly referenced to ferrocene.

2 Syntheses

2.1 General procedure 1 (GP1) for the synthesis of 3a-aa, 3b-ss and 6 via Lithiation-Formylation-Knoevenagel sequence (LiForK)[8]

In a flame-dried Schlenk vessel with magnetic stir bar under nitrogen atmosphere *N*-phenyl dithieno[1,4]thiazine **2a-aa** or **2b-ss** or 3,7-dibromo-10-phenyl-10*H*-phenothiazine (**6**) (1.00 equiv) and tetramethylethylenediamine (2.50 equivs) were dissolved in dry THF (10 mL/mmol) and cooled to -78 °C (isopropanol/dry ice). Then, *n*-butyllithium (2.50 equivs, 1.6 M in hexane) was added dropwise slowly via syringe to the vigorously stirred solution. Stirring was continued at -78°C for 2 h. Then dry DMF (3.00 eq.) was added, stirring was continued at -78 °C for another 90 min and then at ambient temperature for 30 min. To the reaction mixture acetic acid (5.00 eq.) was added. After stirring for 15 min at ambient temperature,

malononitrile (**4**) (3.00 eq.) was added and the stirring was continued for 20 – 40 min at ambient temperature. The volatiles were removed by evaporation and the crude product was purified by flash column chromatography and further suspension in ethanol. For Experimental details see Table S1.

Table S1. Experimental details **GP1**.

Entry	Thiazine [mg] (mmol)	DMF [µl] (mmol)	Malononitrile (**4**) [mg] (mmol)	$t_{Knoevenagel}$ [min]	Yield [mg] (%)
1	146 (0.510) of **2a-aa**	118 (1.53)	101 (1.53)	20	185 (84) of **3a-aa**
2	311 (1.08) of **2b-ss**	249 (3.24)	214 (3.24)	40	191 (40) of **3b-ss**
3[a]	433 (1.50) of **5**	346 (4.50)	297 (4.50)	30	315 (74) of **6**

a: Stirring for 30 min for lithiation.

2.1.1 2,2'-((8-Phenyl-8*H*-dithieno[3,2-*b*:2',3'-*e*][1,4]thiazine-2,6-diyl)bis(methanylylidene))dimalononitrile (3a-aa)

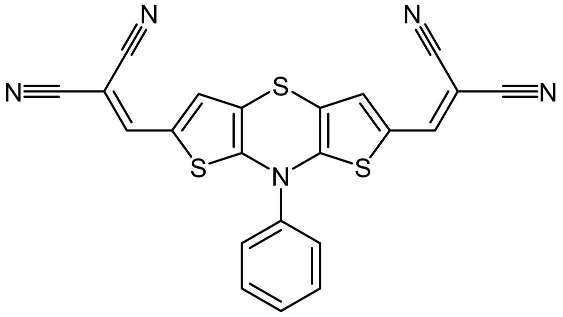

The crude product was synthesized according to **GP1** and purified by flash column chromatography using gradient elution (*n*-hexane/ethyl acetate 2:1 → ethyl acetate) and suspension in ethanol to give product **3a-aa** (185 mg, 88%) as a dark blue powder.

Mp.: 316–320 °C (decomposition). R_f (*n*-hexane/ethyl acetate 2:1) = 0.23. ^{1}H NMR (600 MHz, DMSO-d$_6$, 372K): δ 7.51 (s, 2H), 7.69 – 7.75 (m, 3H), 7.77 - 7.81 (m, 2H), 8.10 (s, 2H). ^{13}C NMR (150 MHz, DMSO-d$_6$): δ 69.2 (C$_{quat}$), 110.8 (C$_{quat}$), 114.5 (C$_{quat}$), 115.2 (C$_{quat}$), 124.8 (C$_{quat}$), 127.7 (CH), 131.6 (CH), 131.7 (CH), 132.0 (CH), 139.8 (C$_{quat}$), 150.1 (CH), 152.2 (C$_{quat}$). MS(MALDI-TOF) *m/z*: 438.980 ([M]$^+$). IR: $\tilde{\nu}$ [cm^{-1}] = 2212 (w), 1561 (s), 1555 (s), 1501

(w), 1489 (w), 1423 (w), 1368 (s), 1325 (s), 1314 (s), 1287 (s), 1273 (s), 1254 (s), 1209 (s), 1169 (s), 1148 (s), 1121 (s), 1074 (m), 1057 (m), 1026 (m), 930 (m), 887 (m), 866 (m), 851 (m), 810 (m), 797 (m), 748 (m), 691 (s), 648 (m), 604 (s). Anal. calcd. for $C_{22}H_9N_5S_3$ (439.5): C 60.12, H 2.06, N 15.93, S 21.88; Found: C 59.89, H 1.92, N 15.73, S 22.08.

2.1.2 2,2'-((4-Phenyl-4*H*-dithieno[2,3-*b*:3',2'-*e*][1,4]thiazine-2,6-diyl)bis(methanylylidene))dimalononitrile (3b-ss)

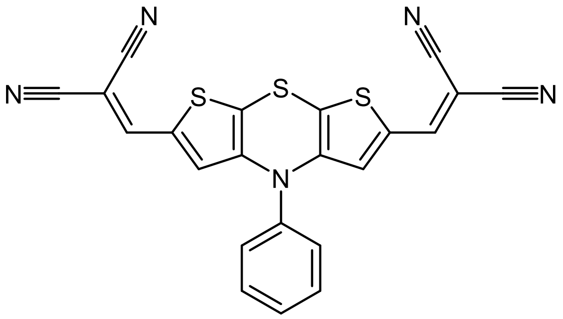

The crude product was synthesized according to **GP1** and purified by flash column chromatography using gradient elution (*n*-hexane/ethyl acetate 2:1 → ethyl acetate) and suspension in ethanol to give product **3b-ss** (191 mg, 40%) as a dark blue powder.

Mp.: 330–335 °C (decomposition). R_f (*n*-hexane/ethyl acetate 2:1) = 0.30. ^{1}H NMR (600 MHz, DMSO-d$_6$, 372K): δ 6.78 (s, 2H), 7.47 – 7.50 (m, 2H), 7.52 – 7.56 (m, 1H), 7.62 – 7.66 (m, 2H), 8.23 (s, 2H). ^{13}C NMR (150 MHz, DMSO-d$_6$, 372K): δ 73.7 (C_{quat}), 113.1 (C_{quat}), 113.8 (C_{quat}), 118.3 (C_{quat}), 125.8 (CH), 127.9(CH), 128.6 (CH), 130.7 (CH), 132.4 (C_{quat}), 140.8 (C_{quat}), 142.8 (C_{quat}), 149.7 (CH). MS(MALDI-TOF) *m*/*z*: 439.073 ([M]$^+$). IR: $\tilde{\nu}$ [cm^{-1}] = 3088 (w), 2988 (w), 2901 (w), 1560 (s), 1549 (s), 1489 (m), 1420 (s), 1369 (s), 1342 (s), 1267 (s), 1209 (s), 1184 (m), 1155 (m), 1111 (m), 1084 (s), 1024 (m), 991 (m), 930 (m), 916 (s), 839 (s), 795 (s), 694 (s), 667 (m). Anal. calcd. for $C_{22}H_9N_5S_3$ (439.5): C 60.12, H 2.06, N 15.93, S 21.88; Found: C 60.00, H 1.96, N 15.68, S 21.64.

2.1.3 2,2'-((10-Phenyl-10*H*-phenothiazine-3,7-diyl)bis(methanylylidene))dimalononitrile (6)

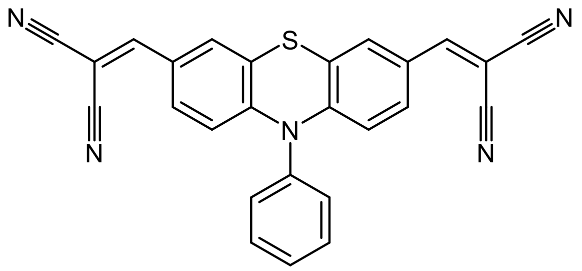

The crude product was synthesized according to **GP1** and purified by flash column chromatography using gradient elution (*n*-hexane/ethyl acetate 2:1 → ethyl acetate) and suspension in ethanol to give product **6** (315 mg, 74%) as a dark red powder.

Mp.: 322-326 °C. R_f (*n*-hexane/ethyl acetate 2:1) = 0.35. ^{1}H NMR (500 MHz, DMSO-d$_6$): δ 6.16 (d, $^3J_{HH}$ = 8.83 Hz, 2H), 7.48 – 7.56 (m, 4H), 7.61 (d, $^4J_{HH}$ = 2.15 Hz, 2H), 7.62 – 7.70 (m, 1H), 7.72 – 7.78 (m, 2H), 8.21 (s, 2H). ^{13}C NMR (125 MHz, DMSO-d$_6$): δ 77.8 (C_{quat}), 99.4 (C_{quat}), 113.5 (C_{quat}), 114.4 (C_{quat}), 116.4 (CH), 118.6 (C_{quat}), 126.8 (C_{quat}), 128.2 (CH), 129.7 (CH), 129.8 (CH), 131.2 (CH), 131.6 (CH), 146.1 (C_{quat}), 158.4 (CH). MS(EI) *m*/*z*: 428 ([M + H]$^+$, 32), 427 ([M]$^+$, 100), 401 ([M – CN]$^+$, 13), 400 ([M – HCN]$^+$, 35), 350 ([M – C_6H_5]$^+$, 22), 349 (16), 77 (25), 51 (17). IR: $\tilde{\nu}$ [cm^{-1}] = 2218 (w), 1557 (m), 1518 (w), 1508 (w), 1474 (s), 1443 (w), 1383 (m), 1325 (m), 1298 (m), 1273 (m), 1248 (m), 1223 (s), 1200 (m), 1179 (s), 1148 (m), 1113 (m), 1065 (m), 1038 (m), 1020 (m), 970 (w), 939 (m), 916 (m), 883 (m), 804 (s), 773 (s), 716 (s), 692 (s), 646 (m), 611 (s). Anal. calcd. for $C_{26}H_{13}N_5S$ (427.5): C 73.05, H 3.07, N 16.38, S 7.50; Found: C 72.83, H 3.08, N 16.26, S 7.26.

2.2 General procedure 2 (GP2) for the synthesis of 3c-aa – 3f-ss via dilithiation–lithium–zinc exchange–Negishi coupling[9]

In a flame-dried Schlenk vessel with magnetic stir bar under nitrogen atmosphere *N*-phenyl dithieno[1,4]thiazine **2a-aa** or **2b-ss** (1.00 equiv) and tetramethylethylenediamine (2.50 equivs) were dissolved in dry THF (10 mL/mmol) and cooled to -78 °C (isopropanol/dry ice). Then, *n*-butyllithium (2.50 equivs, 1.6 M in hexane) was added dropwise slowly via syringe to the vigorously stirred solution. Stirring was continued at -78°C for 2 h, while zinc dibromide (3.00 equivs) was vacuum dried at 120 °C for 1.5 h. After cooling to ambient temperature dry THF (1.0 mL/mmol) was added to zinc dibromide. The resulting zinc dibromide solution (1.0 M in THF) was added dropwise to the reaction mixture, which was stirred at -78 °C for 30 min. After slowly warming to ambient temperature, tetrakis(triphenylphosphane)palladium(0) (5 mol%) and 4-bromobenzonitrile (**7**) or 4-iodoansole (**8**) (2.50 equivs) were added to the reaction mixture and the reaction solution was stirred at 70 °C for 1 – 2 h. The volatiles were removed by evaporation and the crude product was purified by flash column chromatography and further suspension in acetone. For experimental details, see table 2.

Table 2. Experimental details **GP2**.

Dithieno[1,4]thiazine **2** [mg] (mmol)	Aryl halide [mg] (mmol)	$t_{Negishi}$ [h]	Yield **3** [mg] (%)
206 (0.720) of **2a-aa**	328 (1.80) of **7**	1	249 (71) of **3c-aa**
287 (1.00) of **2b-ss**	455 (2.50) of **7**	2	99 (20) of **3d-ss**
277 (0.960) of **2a-aa**	562 (2.40) of **8**	1	314 (65) of **3e-aa**
98.0 (0.340) of **2b-ss**	199 (0.850) of **8**	1	87.0 (51) of **3f-ss**

2.2.1 4,4'-(8-Phenyl-8*H*-dithieno[3,2-*b*:2',3'-*e*][1,4]thiazine-2,6-diyl)dibenzonitrile (3c-aa)

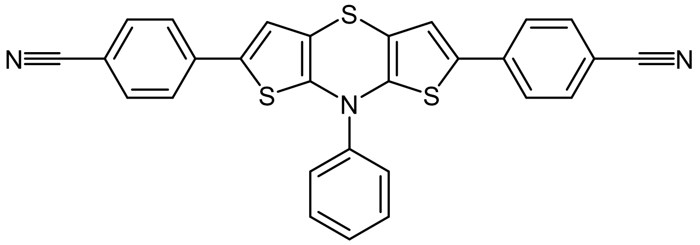

The crude product was synthesized according to **GP2** and purified by flash column chromatography using gradient elution (*n*-hexane/ethyl acetate 4:1 with 1% triethyl amine → *n*-hexane/ethyl acetate 1:1 with 1% triethyl amine) and suspension in acetone to give product **3c-aa** (249 mg, 71%) as a violet powder.

Mp 274–276 °C. R_f (*n*-hexane/ethyl acetate 3:1) = 0.36. ^{1}H NMR (600 MHz, DMSO-d$_6$, 393K): δ 6.23 (s, 2H), 7.54 –7.57 (m, 1H), 7.57 – 7.60 (m, 4H), 7.62 – 7.66 (m, 2H), 7.66 – 7.70 (m, 6H). ^{13}C NMR (150 MHz, DMSO-d$_6$, 393K): δ 108.3 (C$_{quat}$), 108.6 (C$_{quat}$), 117.8 (C$_{quat}$), 122.9 (CH), 124.0 (CH), 126.7 (CH), 128.9 (CH), 130.1 (CH), 130.4 (C$_{quat}$), 132.1 (CH), 136.7 (C$_{quat}$), 142.2 (C$_{quat}$), 142.7 (C$_{quat}$). MS(MALDI-TOF) *m*/*z*: 489.115 ([M]$^+$). IR: $\tilde{\nu}$ [cm^{-1}] 3678 (w), 3057 (w), 2990 (w), 2886 (w), 2218 (w), 1559 (m), 1493 (m), 1435 (s), 1408 (s), 1362 (w), 1296 (w), 1283 (w), 1271 (w), 1227 (w), 1177 (m), 1165 (m),1111 (w), 1045 (w), 1016 (w), 984 (w), 964 (w), 945 (w), 918 (w), 880 (w), 835 (w), 818 (s), 802 (m), 772 (w), 743 (w), 719 (w), 691 (m), 651 (w). Anal. calcd. for $C_{28}H_{15}N_3S_3$ (489.6): C 68.69, H 3.09, N 8.58, S 19.64; Found: C 68.67, H 3.00, N 8.40, S 19.93.

2.2.2 4,4'-(4-Phenyl-4*H*-dithieno[2,3-*b*:3',2'-*e*][1,4]thiazine-2,6-diyl)dibenzonitrile (3d-ss)

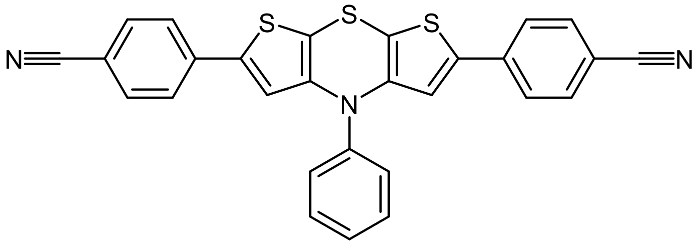

The crude product was synthesized according to **GP2** and purified by flash column chromatography (*n*-hexane/ethyl acetate 4:1 with 1% triethyl amine) and suspension in acetone to give product **3d-ss** (99 mg, 20%) as an orange powder.

Mp 253–256 °C. R_f (*n*-hexane/ethyl acetate 4:1) = 0.38. ^{1}H NMR (300 MHz, DMSO-d$_6$): δ 6.83 (s, 2H), 7.39 –7.46 (m, 1H), 7.46 – 7.51 (m, 2H), 7.52 – 7.60 (m, 2H), 7.61 – 7.68 (m, 4H), 7.74 – 7.82 (m, 4H). ^{13}C NMR (75 MHz, DMSO-d$_6$): δ 107.9 (C$_{quat}$), 109.8 (C$_{quat}$), 118.1 (CH),

118.5 (C_{quat}), 125.0 (CH), 126.6 (CH), 127.2 (CH), 130.4 (CH), 132.9 (CH), 136.7 (C_{quat}), 139.2 (C_{quat}), 142.8 (C_{quat}), 143.7 (C_{quat}). MS(MALDI-TOF) *m*/*z*: 489.124 ($[M]^+$). IR: $\tilde{\nu}$ [cm^{-1}] 2220 (w), 1601 (s), 1497 (s), 1425 (m), 1408 (m), 1371 (m), 1273 (m), 1180 (m), 943 (w), 826 (s), 806 (w), 758 (w), 739 (s), 681 (m). Anal. calcd. for $C_{28}H_{15}N_3S_3$ (489.6): C 68.69, H 3.09, N 8.58, S 19.64; Found: C 68.43, H 3.06, N 8.47, S 19.39.

2.2.3 2,6-Bis(4-methoxyphenyl)-8-phenyl-8*H*-dithieno[3,2-*b*:2',3'-*e*][1,4]thiazine (3e-aa)

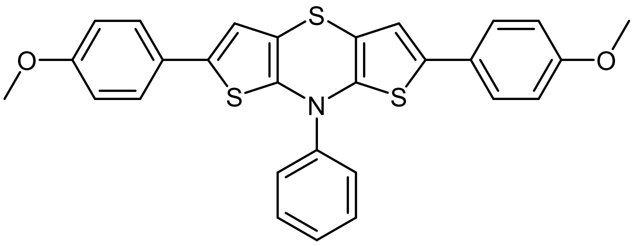

The crude product was synthesized according to **GP2** and purified by flash column chromatography using gradient elution (*n*-hexane/ethyl acetate 3:1 with 1% triethyl amine → *n*-hexane/ethyl acetate 1:1 with 1% triethyl amine) and suspension in acetone to give product **3e-aa** (314 mg, 65%) as an orange powder.

Mp 250–253 °C. R_f (*n*-hexane/ethyl acetate 5:1) = 0.37. ^{1}H NMR (600 MHz, DMSO-d_6, 373K): δ 3.77 (s, 6H), 6.90 (s, 2H), 6.90 –6.93 (m, 4H), 7.37 – 7.40 (m, 4H), 7.44 – 7.47 (m, 1H), 7.56 – 7.59 (m, 2H), 7.60 – 7.63 (m, 2H). ^{13}C NMR (150 MHz, DMSO-d_6, 373K): δ 54.9 (CH_3), 99.0 (C_{quat}), 114.2 (CH), 118.9 (CH), 125.4 (CH), 125.5 (CH), 125.7 (CH), 127.6 (C_{quat}), 129.8 (CH), 133.9 (C_{quat}), 139.6 (C_{quat}), 143.7 (C_{quat}), 158.6 (C_{quat}). MS(ESI-HRMS) *m*/*z*: Calcd. for $C_{28}H_{21}NO_2S_3$: 499.0734, Found: 499.0735 ($[M]^+$). IR: $\tilde{\nu}$ [cm^{-1}] 3026 (w), 3005 (w), 2930 (w), 2905 (w), 2833 (w), 1603 (w), 1558 (w), 1501 (s), 1489 (s), 1472 (m), 1449 (s), 1337 (w),1283 (m), 1244 (s), 1223 (m), 1179 (m), 1159 (m), 1125 (w), 1111 (m), 1072 (w), 1026 (s), 962 (w), 858 (w), 818 (s), 802 (m), 789 (m), 741 (w), 694 (w), 664 (s). Anal. calcd. for $C_{28}H_{21}NO_2S_3$ (499.7): C 67.31, H 4.24, N 2.80, S 19.25; Found: C 67.30, H 4.34, N 2.75, S 19.55.

2.2.4 2,6-Bis(4-methoxyphenyl)-4-phenyl-4*H*-dithieno[2,3-*b*:3',2'-*e*][1,4]thiazine (3f-ss)

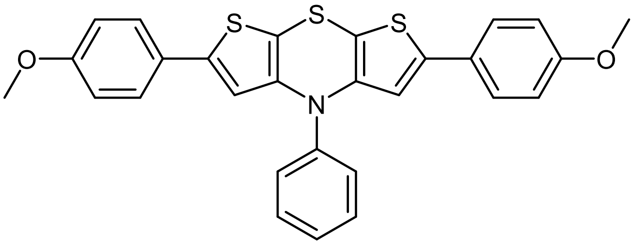

The crude product was synthesized according to **GP2** and purified by flash column chromatography (*n*-hexane/ethyl acetate 3:1 with 1% triethyl) and suspension in acetone to give product **3f-ss** (87 mg, 51%) as a yellow powder.

Mp 237–239 °C. R_f (*n*-hexane/ethyl acetate 3:1) = 0.61. ^{1}H NMR (600 MHz, DMSO-d_6, 373K): δ 3.77 (s, 6H), 6.48 (s, 2H), 6.89 –6.95 (m, 4H), 7.33 – 7.38 (m, 4H), 7.38 – 7.43 (m, 1H), 7.45 – 7.50 (m, 2H), 7.52 – 7.58 (m, 2H). ^{13}C NMR (150 MHz, DMSO-d_6, 373K): δ 54.9 (CH_3), 103.7 (C_{quat}), 114.3 (CH), 115.0 (CH), 125.3 (C_{quat}), 125.8 (CH), 126.1 (CH), 126.4 (CH), 129.7 (CH), 141.3 (C_{quat}), 143.6 (C_{quat}), 143.2 (C_{quat}), 159.0 (C_{quat}). MS(MALDI-TOF) *m*/*z*: 499.090 ([M]$^+$). IR: $\tilde{\nu}$ [cm^{-1}] 2995 (w), 2907 (w), 2835 (w), 1603 (w), 1504 (m), 1489 (s), 1454 (m), 1441 (m), 1369 (m), 1292 (m), 1250 (s), 1233 (m), 1206 (w), 1177 (m), 1169 (m), 1157 (m), 1128 (w), 1113 (m), 1028 (s), 1001 (m), 980 (w), 908 (w), 826 (s), 806 (s), 783 (m), 729 (s), 696 (s), 675 (w), 667 (w). Anal. calcd. for $C_{28}H_{21}NO_2S_3$ (499.7): C 67.31, H 4.24, N 2.80, S 19.25; Found: C 67.21, H 4.20, N 2.80, S 19.13.

3 ^{1}H and ^{13}C NMR spectra

3.1 2,2'-((8-Phenyl-8*H*-dithieno[3,2-*b*:2',3'-*e*][1,4]thiazine-2,6-diyl)bis(methanylylidene))dimalononitrile (3a-aa)

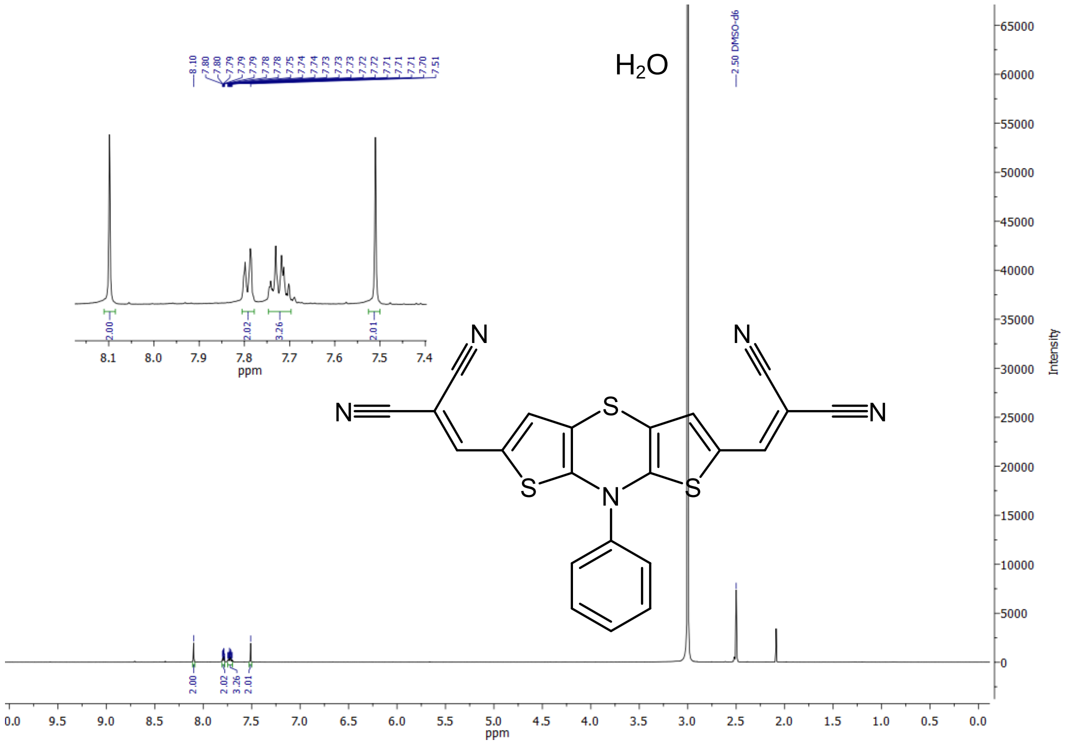

Figure S1. ^{1}H NMR spectrum of **3a-aa** (DMSO-d_6, 372 K, 600 MHz).

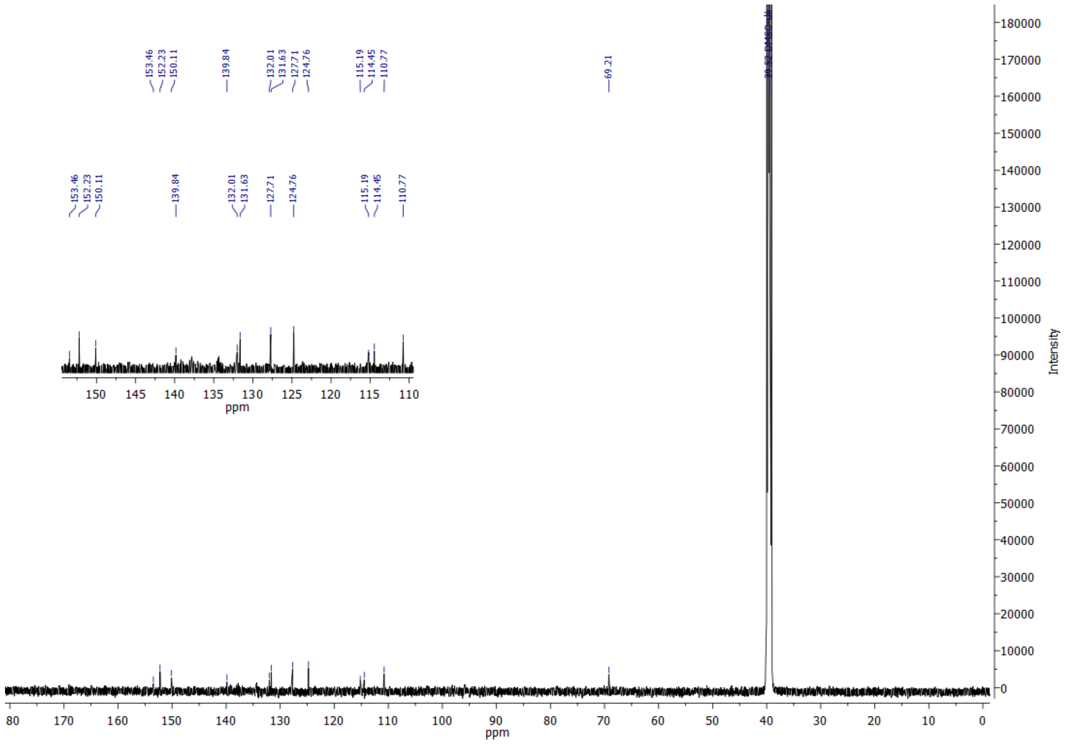

Figure S2. ^{13}C-NMR spectrum of **3a-aa** (DMSO-d_6, 293 K, 150 MHz).

3.2 2,2'-((4-phenyl-4*H*-dithieno[2,3-*b*:3',2'-*e*][1,4]thiazine-2,6-diyl)bis(methanylylidene))dimalononitrile (3b-ss)

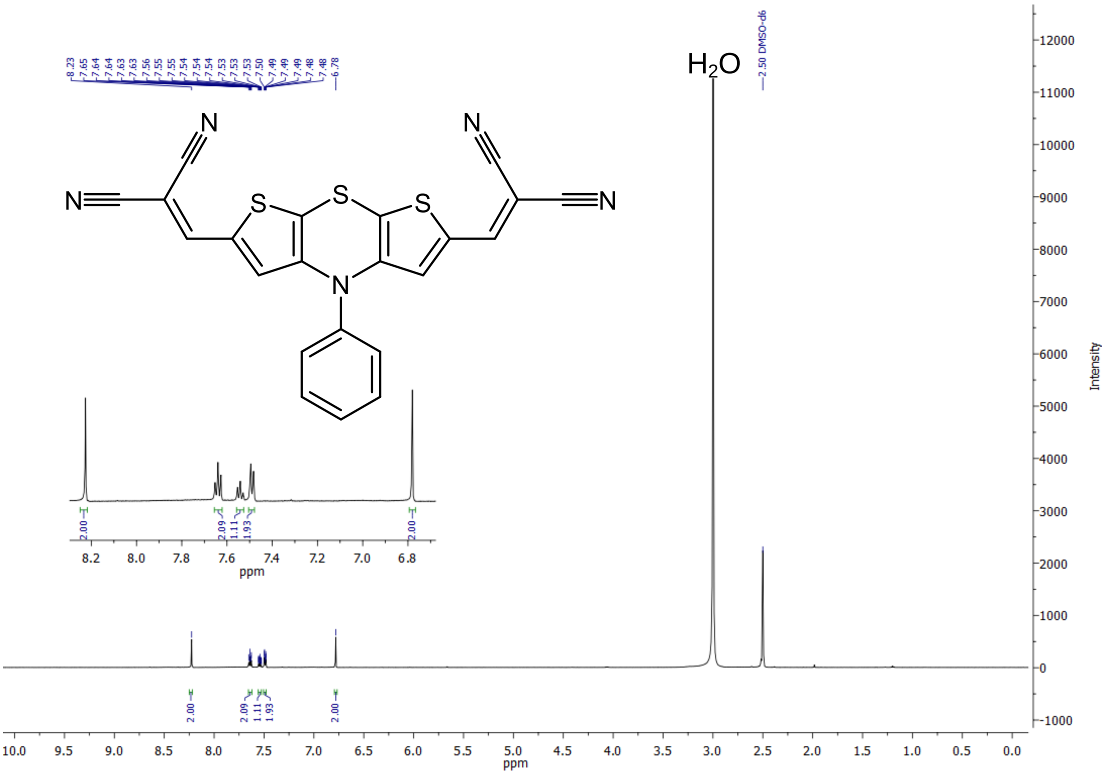

Figure S3. ^{1}H NMR spectrum of **3b-ss** (DMSO-d$_6$, 372 K, 600 MHz).

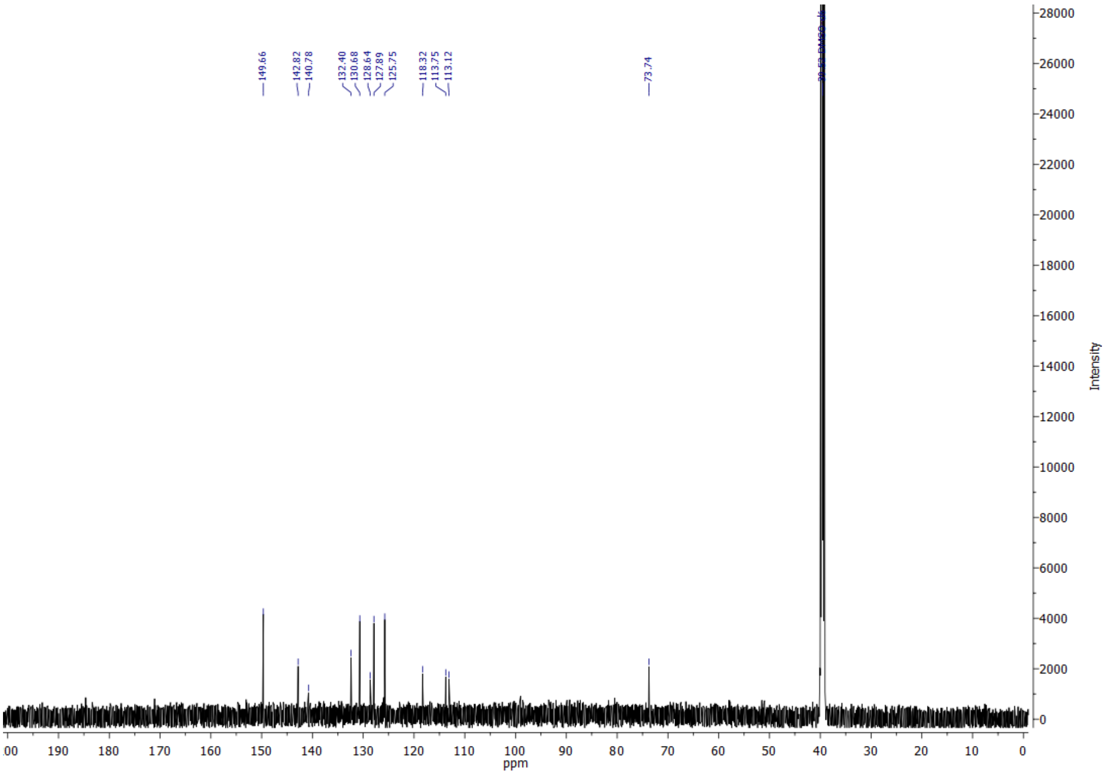

Figure S4. ^{13}C NMR spectrum of **3b-ss** (DMSO-d$_6$, 372 K, 150 MHz).

3.3 2,2'-((10-Phenyl-10*H*-phenothiazine-3,7-diyl)bis(methanylylidene))dimalononitrile (6)

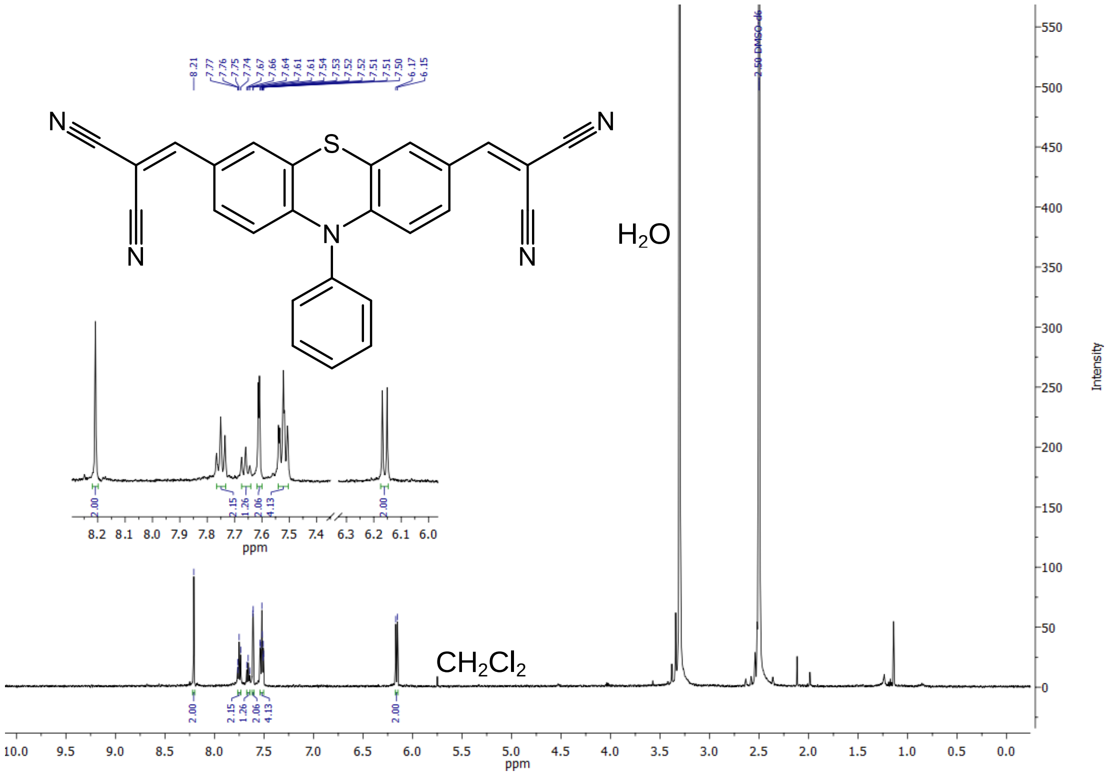

Figure S5. ^{1}H NMR spectrum of **6** (DMSO-d$_6$, 298 K, 500 MHz).

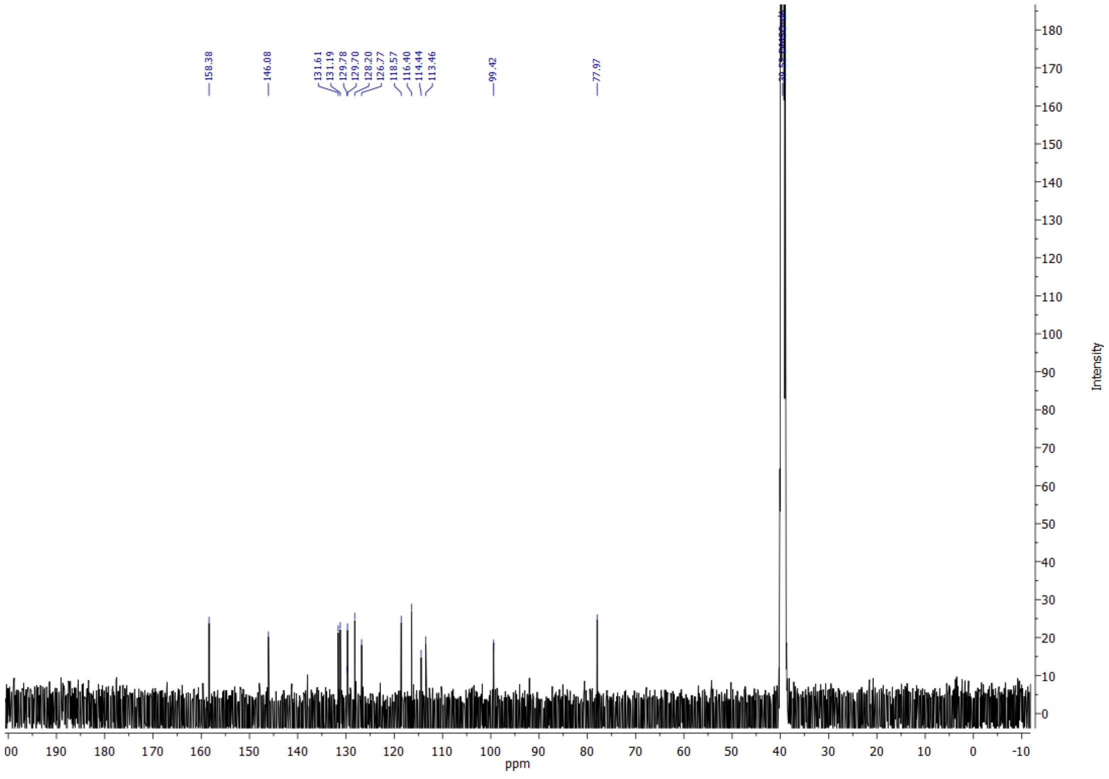

Figure S6. ^{13}C-NMR spectrum of **6** (DMSO-d$_6$, 298 K, 125 MHz).

3.4 4,4'-(8-Phenyl-8*H*-dithieno[3,2-*b*:2',3'-*e*][1,4]thiazine-2,6-diyl)dibenzonitrile (3c-aa)

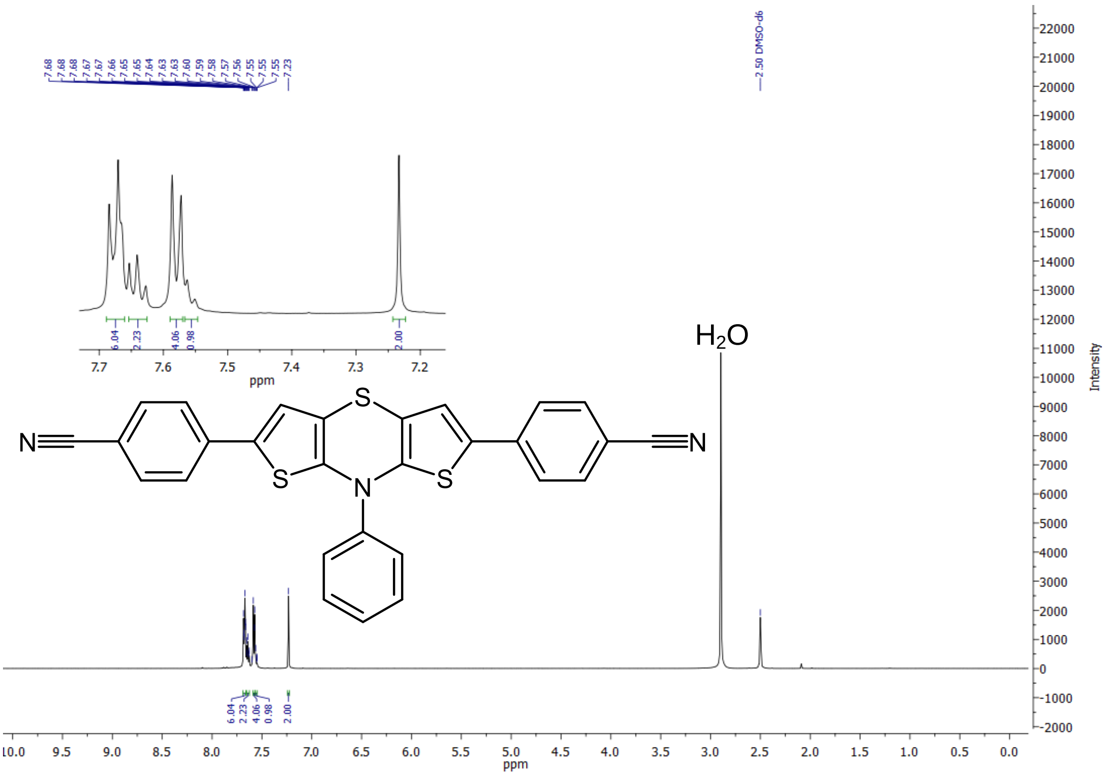

Figure S7. ^{1}H NMR spectrum of **3c-aa** (DMSO-d$_6$, 393 K, 600 MHz).

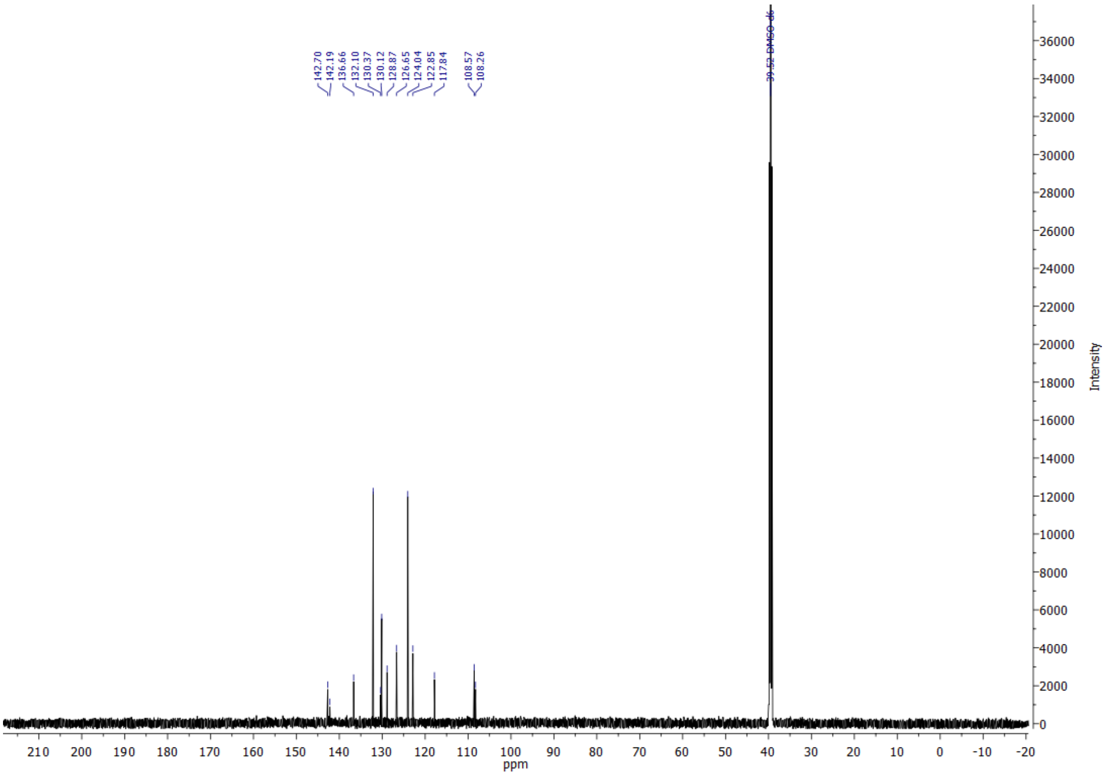

Figure S8. ^{13}C NMR spectrum of **3c-aa** (DMSO-d$_6$, 393 K, 150 MHz).

3.5 4,4'-(4-Phenyl-4*H*-dithieno[2,3-*b*:3',2'-*e*][1,4]thiazine-2,6-diyl)dibenzonitrile (3d-ss)

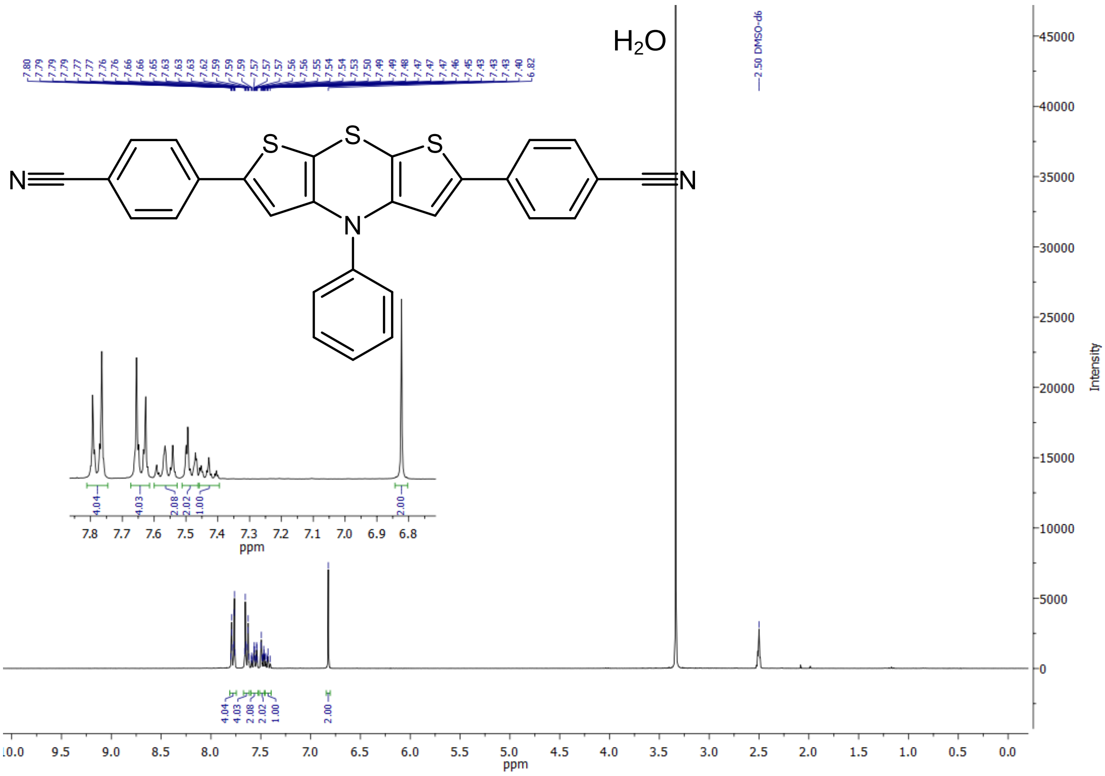

Figure S9. ^{1}H NMR spectrum of **3d-ss** (DMSO-d$_6$, 293 K, 300 MHz).

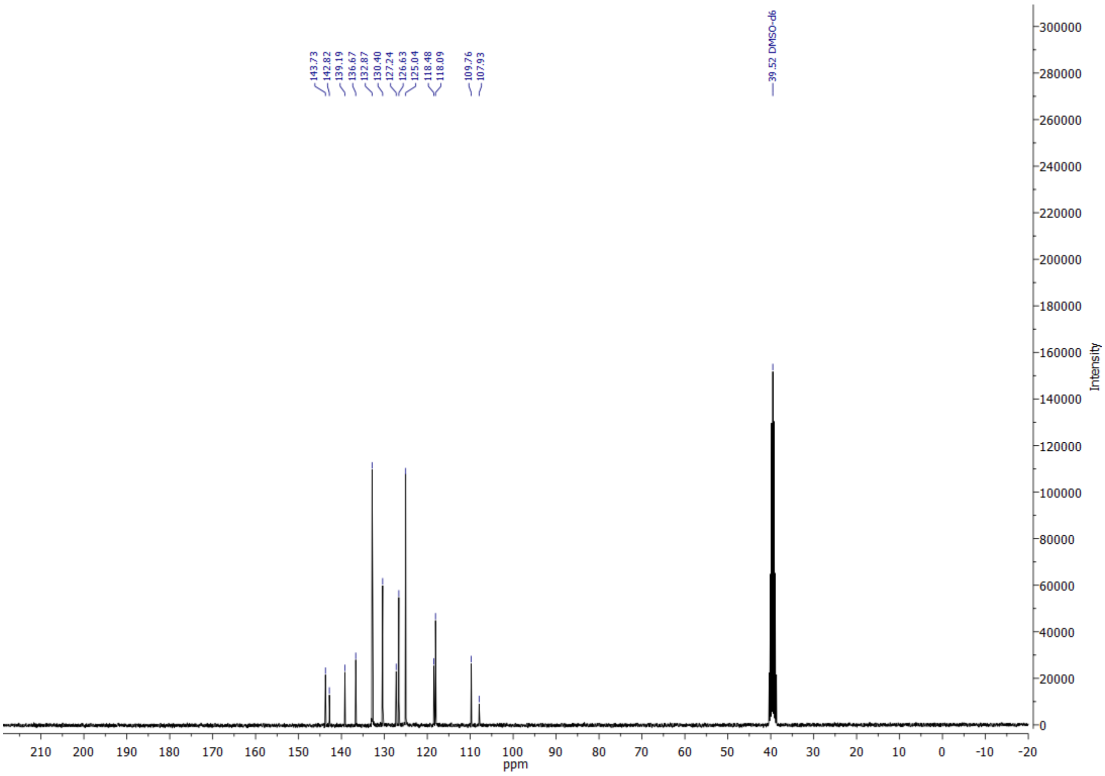

Figure S10. ^{13}C NMR spectrum of **3d-ss** (DMSO-d$_6$, 293 K, 75 MHz).

3.6 2,6-Bis(4-methoxyphenyl)-8-phenyl-8*H*-dithieno[3,2-*b*:2',3'-*e*][1,4]thiazine (3e-aa)

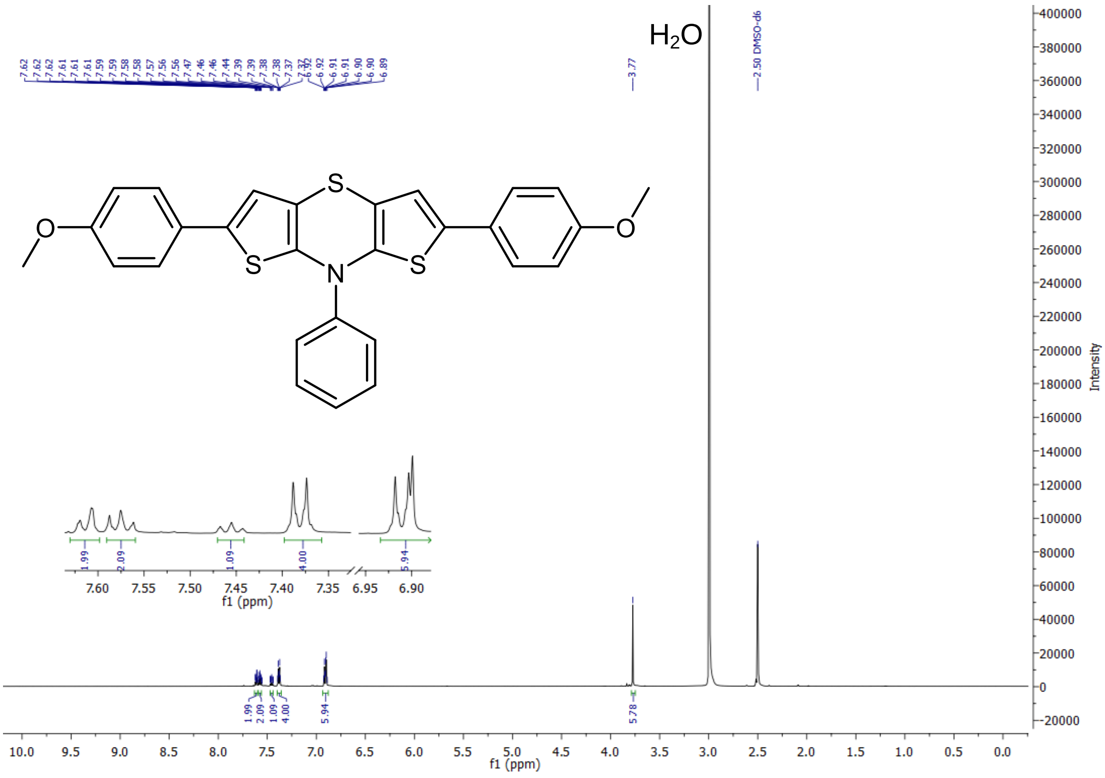

Figure S11. ^{1}H NMR spectrum of **3e-aa** (DMSO-d$_6$, 373 K, 600 MHz).

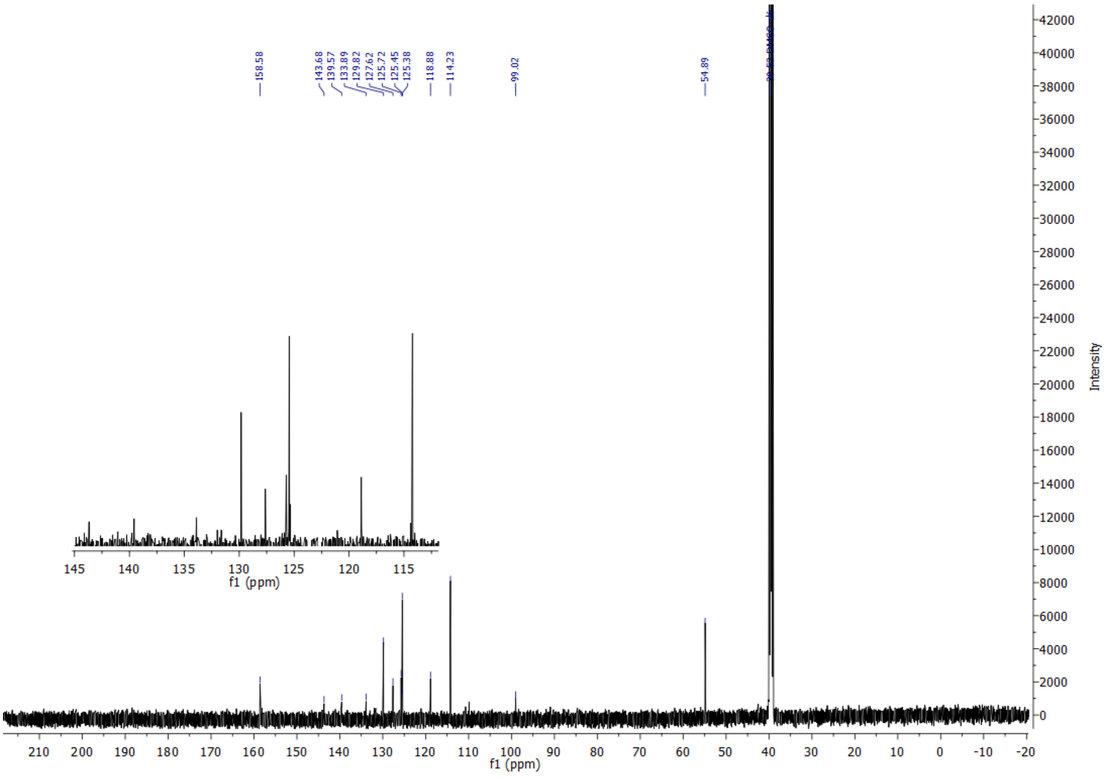

Figure S12. ^{13}C NMR spectrum of **3e-aa** (DMSO-d$_6$, 373 K, 150 MHz).

3.7 2,6-Bis(4-methoxyphenyl)-4-phenyl-4*H*-dithieno[2,3-*b*:3',2'-*e*][1,4]thiazine (3f-ss)

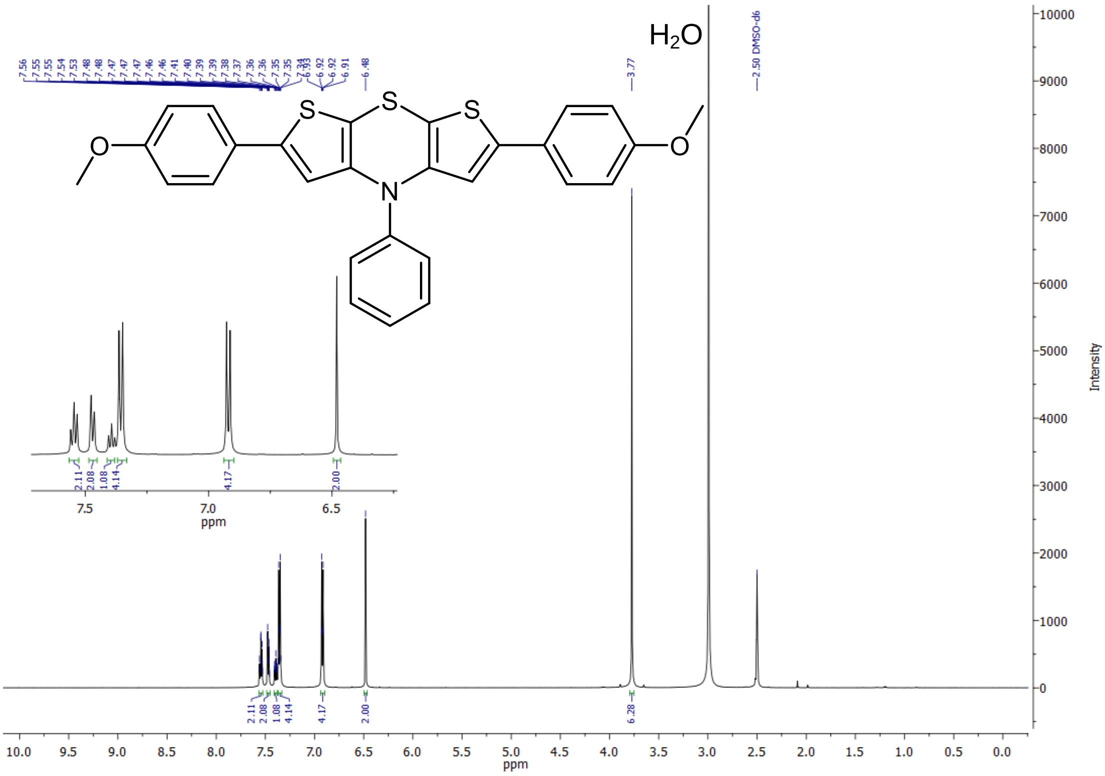

Figure S13. ^{1}H NMR spectrum of **3f-ss** (DMSO-d$_6$, 373 K, 600 MHz).

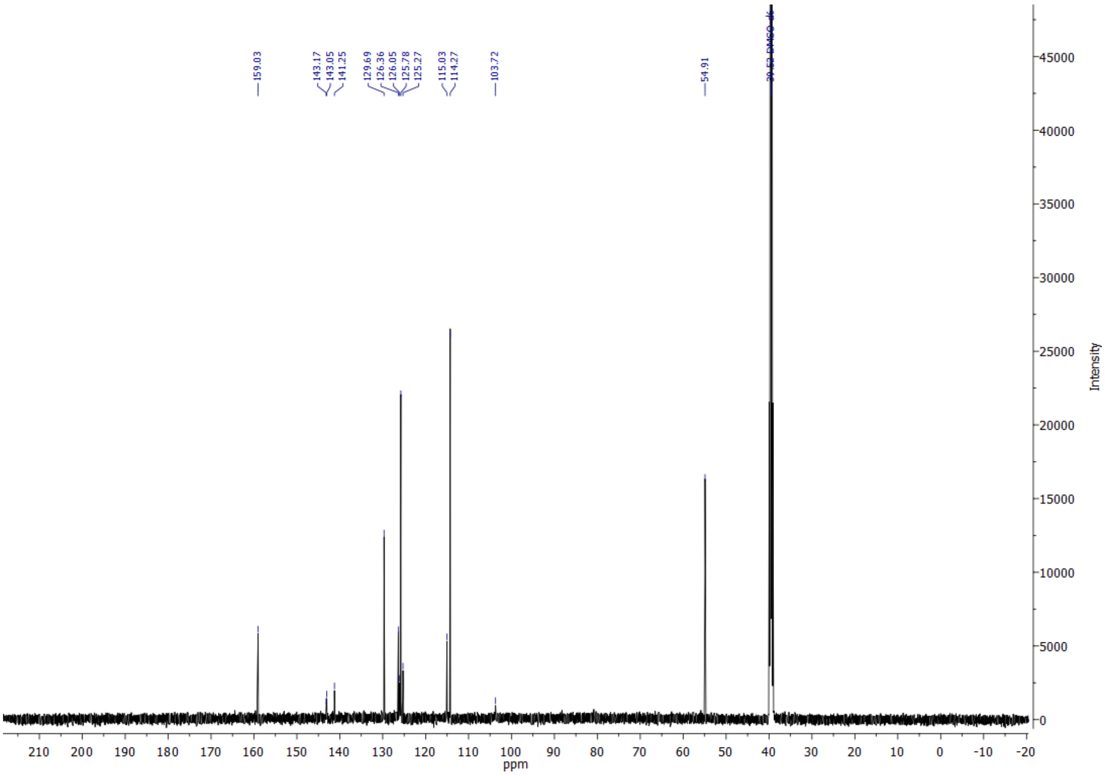

Figure S14. ^{13}C NMR spectrum of **3f-ss** (DMSO-d$_6$, 373 K, 150 MHz).

4 Cyclovoltammetric Data

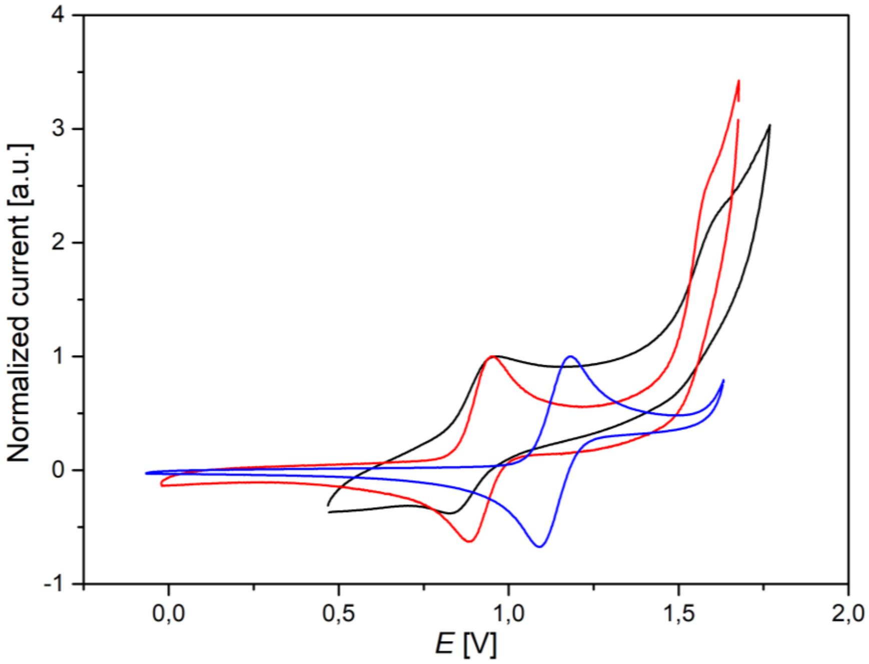

Figure S15. Cyclic voltammograms of **3a-aa (red)**, **3b-ss** (black) and **6** (blue) (CH_2Cl_2, T = 298 K, 0.1 M $[Bu_4N][PF_6]$, v = 100 mV/s, Pt-working, Ag/AgCl-reference and Pt-counter electrode, $[Me_{10}Fc]/[Me_{10}Fc]^+$ as an internal standard; $Me_{10}Fc$ = decamethylferrocene, $E_{0/+1}$ = -95 mV vs. ferrocene with $E_{0/+1}(Fc/Fc^+)$ = 450 mV).

The cyclic voltammograms of **3a-aa** and **3b-ss** were processed by convolution and deconvolution (Figures S16 and S17) for the determination of the second oxidation potentials using the condecon program.[10]

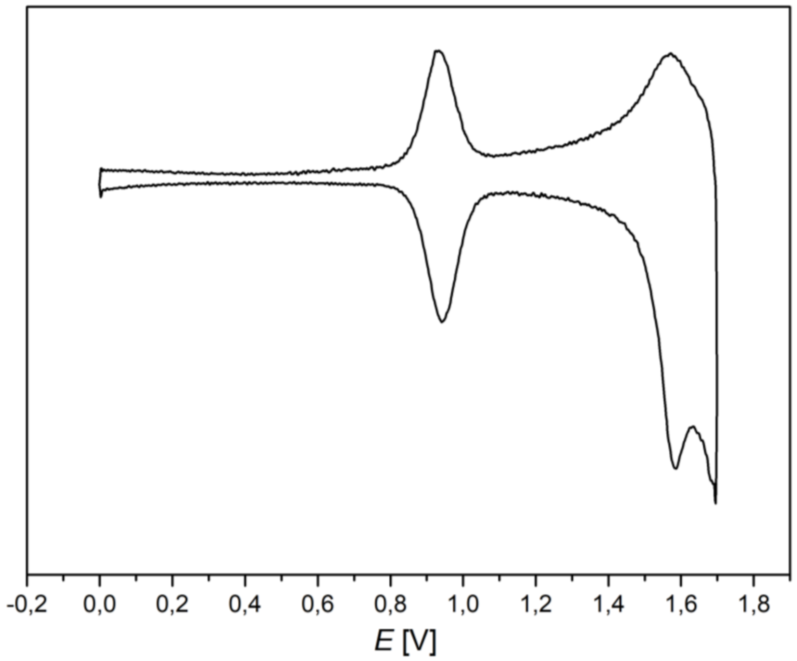

Figure S16. Convolution-deconvolution of the cyclic voltammograms of **3a-aa** (CH_2Cl_2, T = 298 K, 0.1 M $[Bu_4N][PF_6]$, v = 100 mV/s, Pt-working, Ag/AgCl-reference and Pt-counter electrode, $[Me_{10}Fc]/[Me_{10}Fc]^+$ as an internal standard; $Me_{10}Fc$ = decamethylferrocene, $E_{0/+1}$ = -95 mV vs. ferrocene with $E_{0/+1}(Fc/Fc^+)$ = 450 mV).

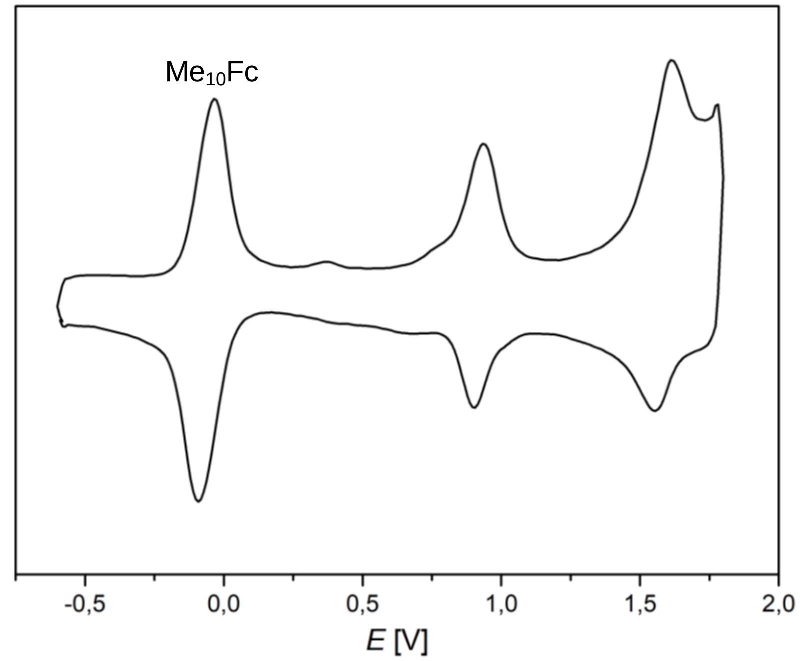

Figure S17. Convolution-deconvolution of the cyclic voltammograms of **3b-ss** (CH_2Cl_2, T = 298 K, 0.1 M $[Bu_4N][PF_6]$, v = 100 mV/s, Pt-working, Ag/AgCl-reference and Pt-counter electrode, $[Me_{10}Fc]/[Me_{10}Fc]^+$ as an internal standard; $Me_{10}Fc$ = decamethylferrocene, $E_{0/+1}$ = -95 mV vs. ferrocene with $E_{0/+1}(Fc/Fc^+)$ = 450 mV).

5 Photophysical Properties of Compounds 3 and 6

5.1 Solvatochromism Studies

Compounds **3a-aa** – **3d-ss** and **6** showed positive solvatochromism since the emission bands are shifted more bathochromically with increasing solvent polarity (Figures S19 – S28). The change of the dipole moments from the electronic ground- to excited-states $\Delta\mu$ were obtained by Lippert-Mataga plots (Figures S19–S28; equations 1, 2).[11]

$$\Delta\tilde{\nu} = \frac{\Delta\mu^2}{2\pi hc\varepsilon_0 a^3}\Delta f \qquad \text{(eq. 1)}$$

Δf orientation polarization

$\Delta\tilde{\nu}$ stokes shift

a Onsager radius

$$\Delta f = \frac{\varepsilon-1}{2\varepsilon+1} - \frac{n^2-1}{2n^2+1} \qquad \text{(eq. 2)}$$

ε solvent permittivity

n refractive index

The Onsager radii were estimated from the optimized ground-state geometries (Figure S18). Due to the C_s symmetry of all compounds, the Onsager radii were measured between the dithienothiazines' centers and acceptor substituents respectively.

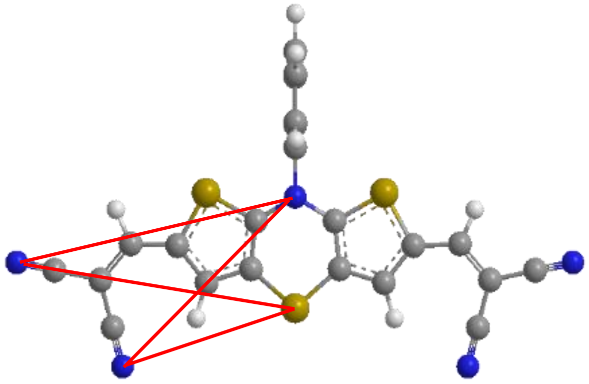

Figure S18. Exemplarily estimation of the Onsager radius of **3a-aa** from the optimized ground-state geometry (mean of marked distances, PBE1PBE/6-31G**, PCM CH_2Cl_2).

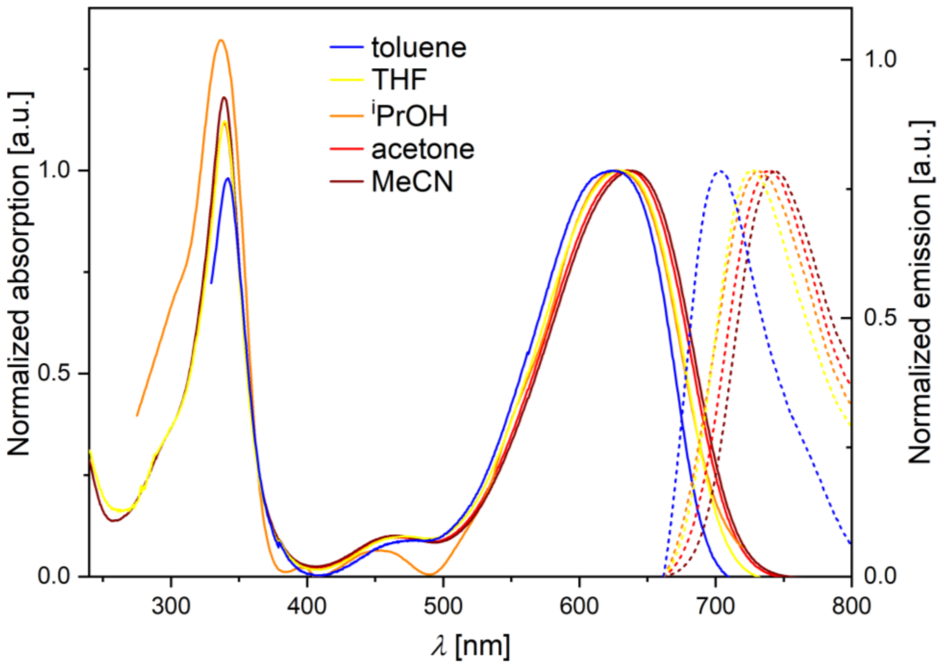

Figure S19. Normalized absorption and emission spectra of **3a-aa** recorded in different solvents (c(**3a-aa**) = 10^{-6} M, 298 K).

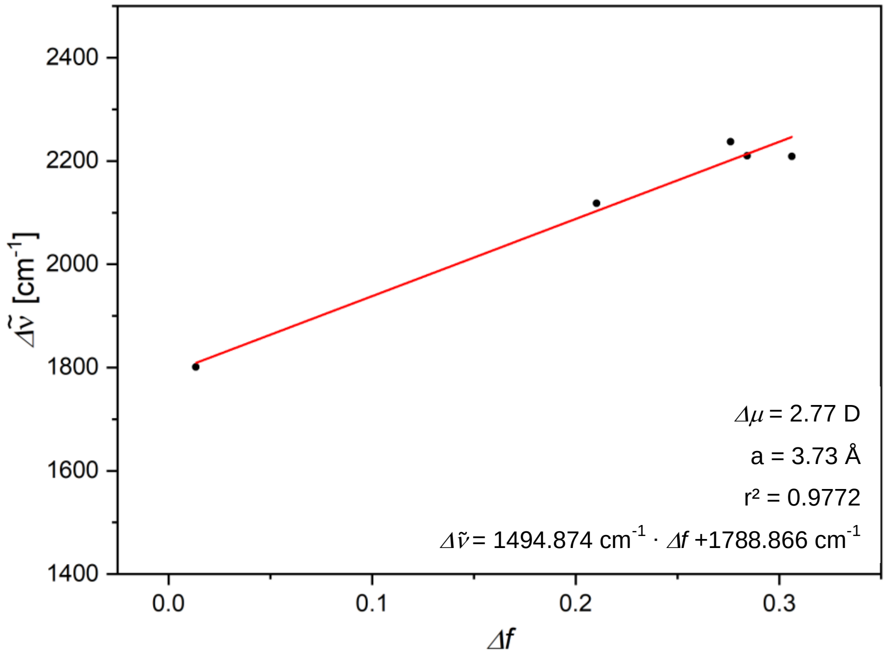

Figure S20. Lippert plot of compound **3a-aa**.

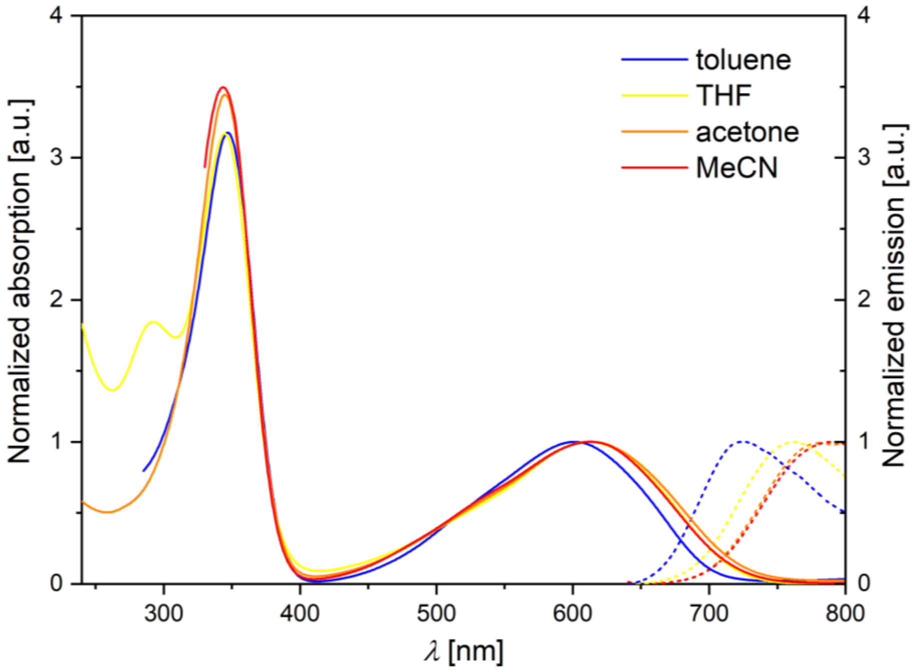

Figure S21. Normalized absorption and emission spectra of **3b-ss** recorded in different solvents (c(**3b-ss**) = 10^{-6} M, 298 K).

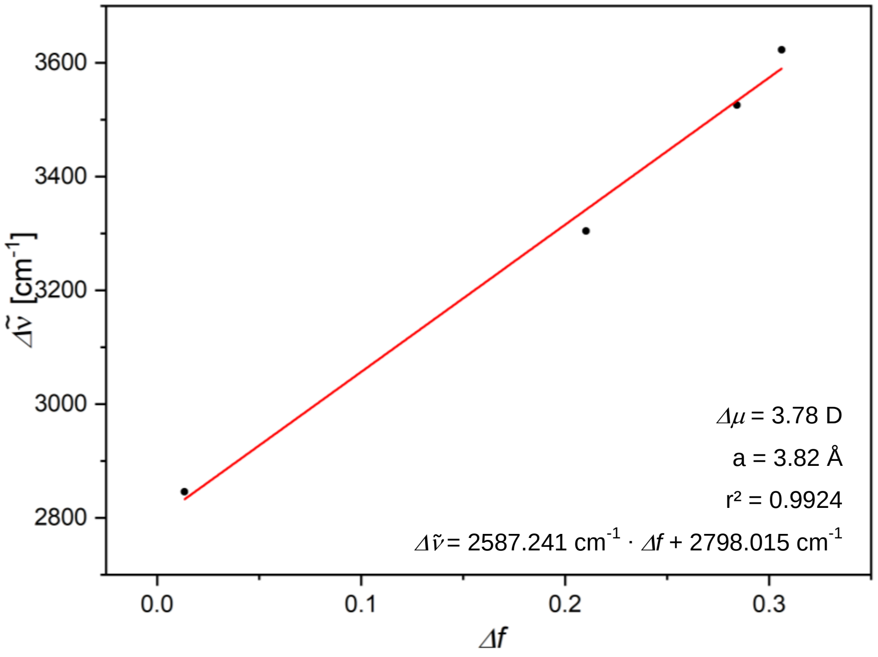

Figure S22. Lippert plot of compound **3b-ss**.

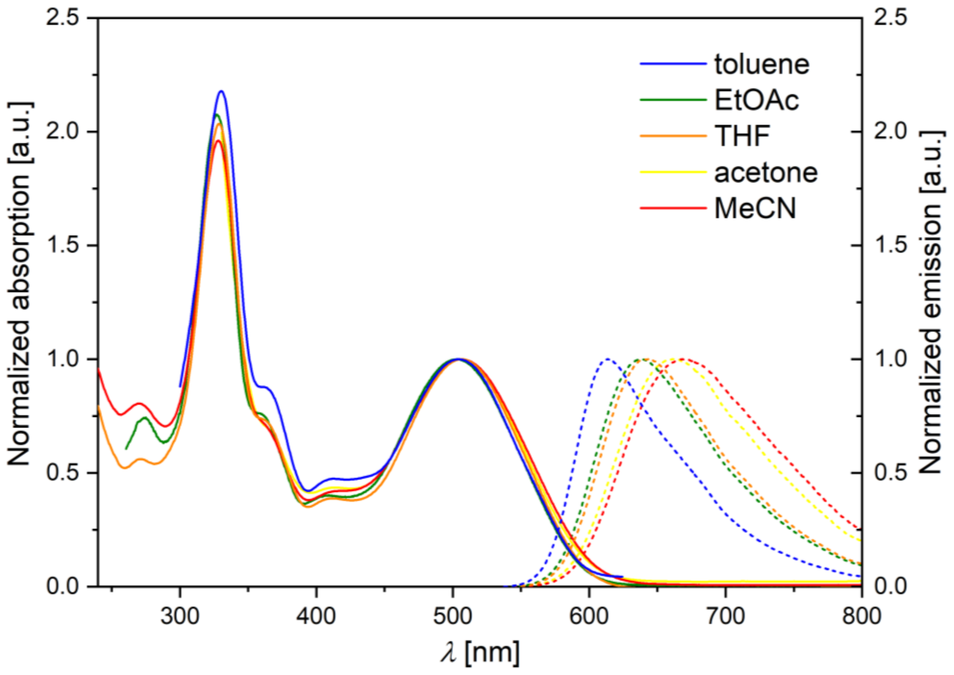

Figure S23. Normalized absorption and emission spectra of **6** recorded in different solvents (c(**6**) = 10^{-6} M, 298 K).

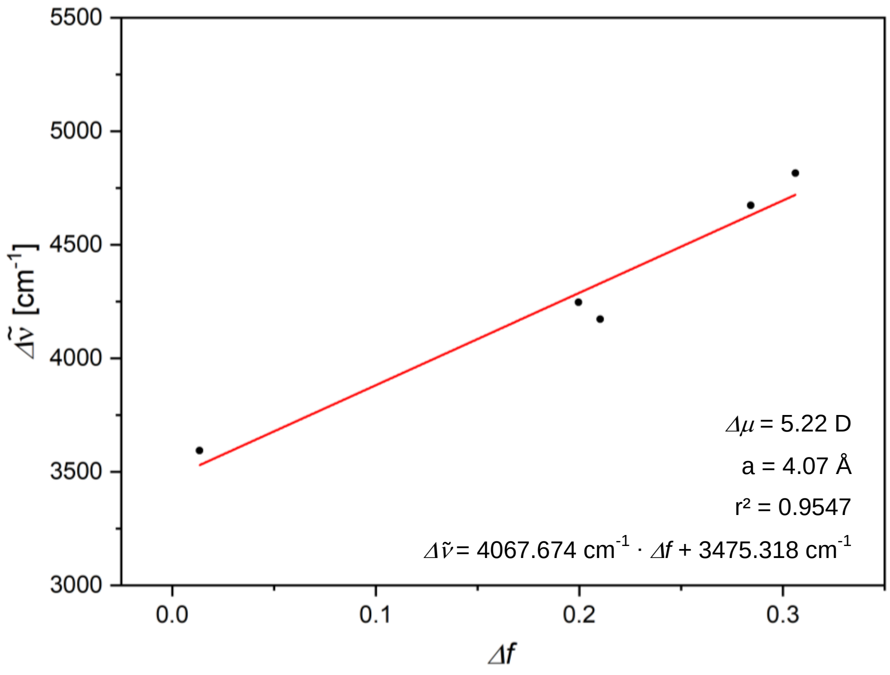

Figure S24. Lippert plot of compound **6**.

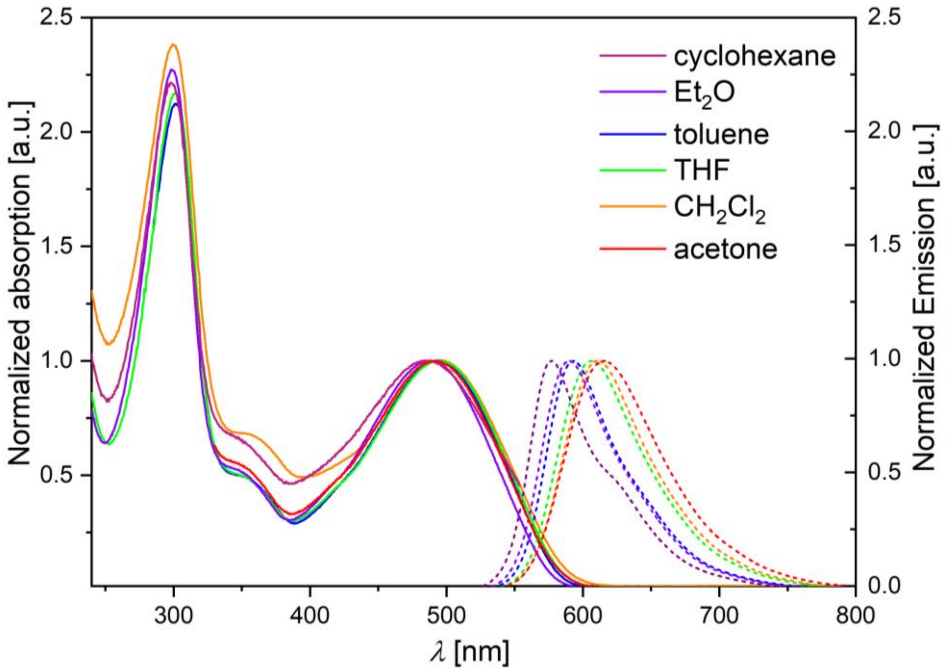

Figure S25. Normalized absorption and emission spectra of **3c-aa** recorded in different solvents (c(**3c-aa**) = 10^{-6} M, 298 K).

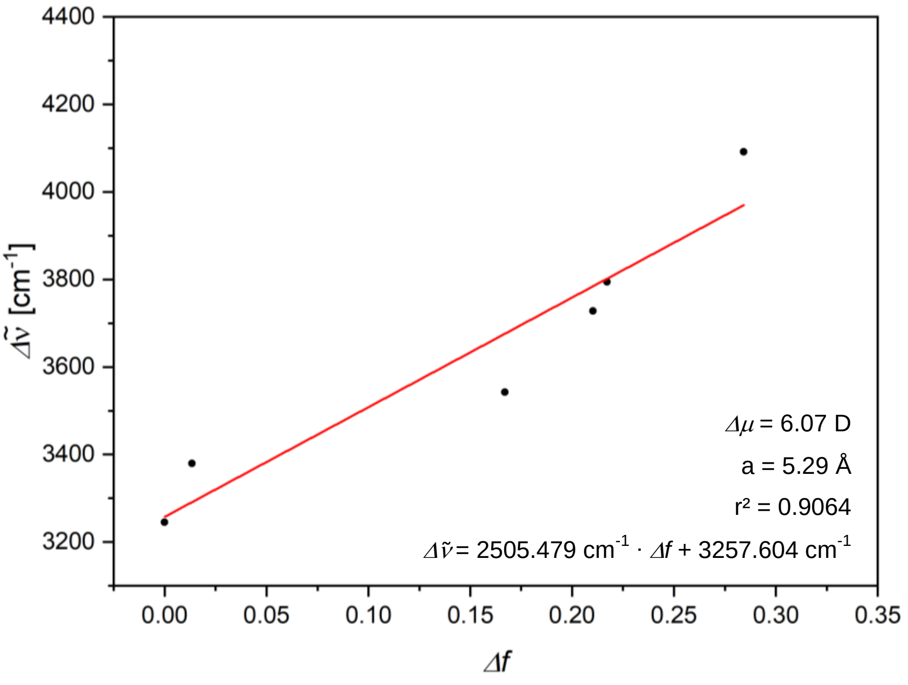

Figure S26. Lippert plot of compound **3c-aa**.

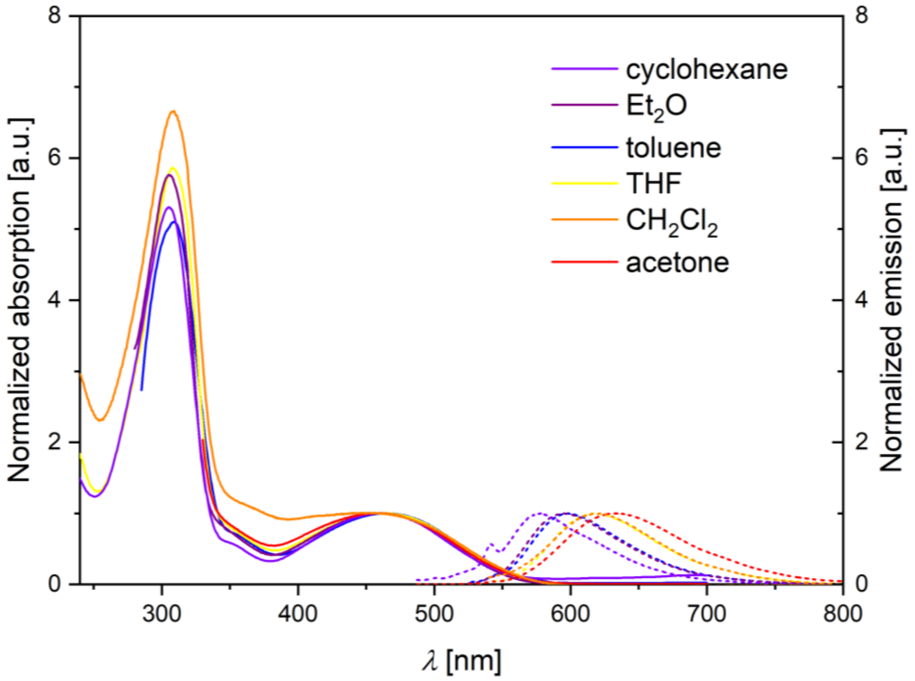

Figure S27. Normalized absorption and emission spectra of **3d-ss** recorded in different solvents (c(**3d-ss**) = 10^{-6} M, 298 K).

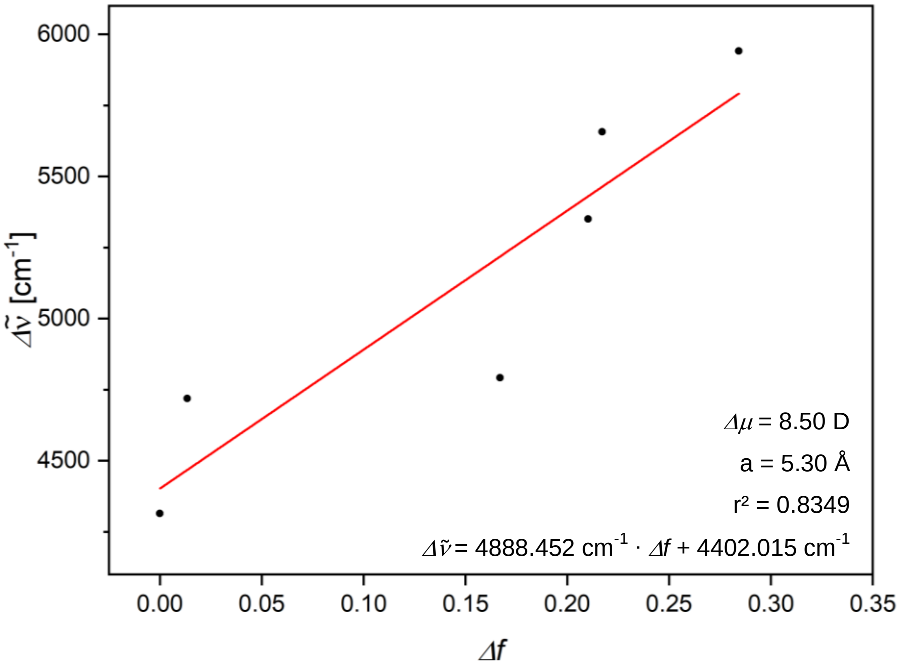

Figure S28. Lippert plot of compound **3d-ss**.

5.2 Fluorescence Lifetimes of 3a-aa and 3b-ss and Phosphorescence Lifetime of 3f-ss

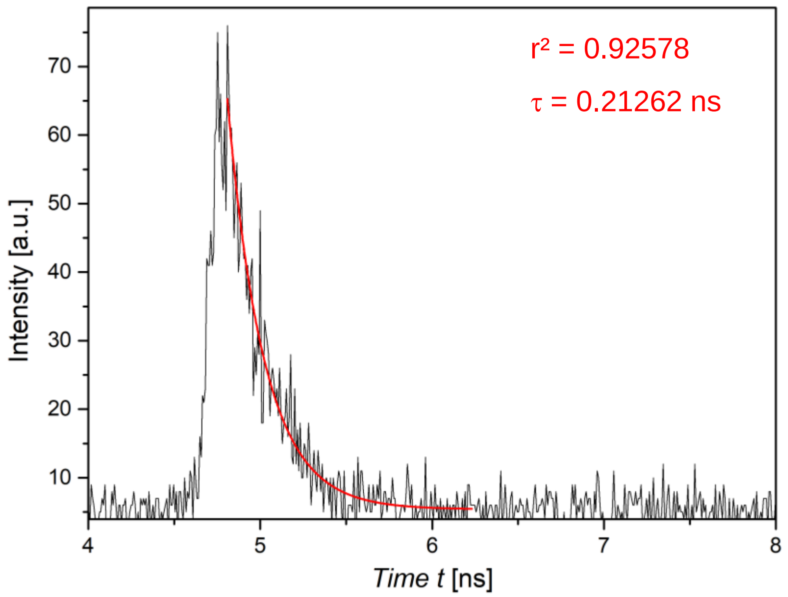

Figure S29. Exponential fit and fluorescence lifetime τ (red) of the fluorescence decay of compound **3b-ss** (CH_2Cl_2, c(**3b-ss**) = 10^{-6} M, 298 K, λ_{exc} = 623 nm, λ_{em} = 750nm).

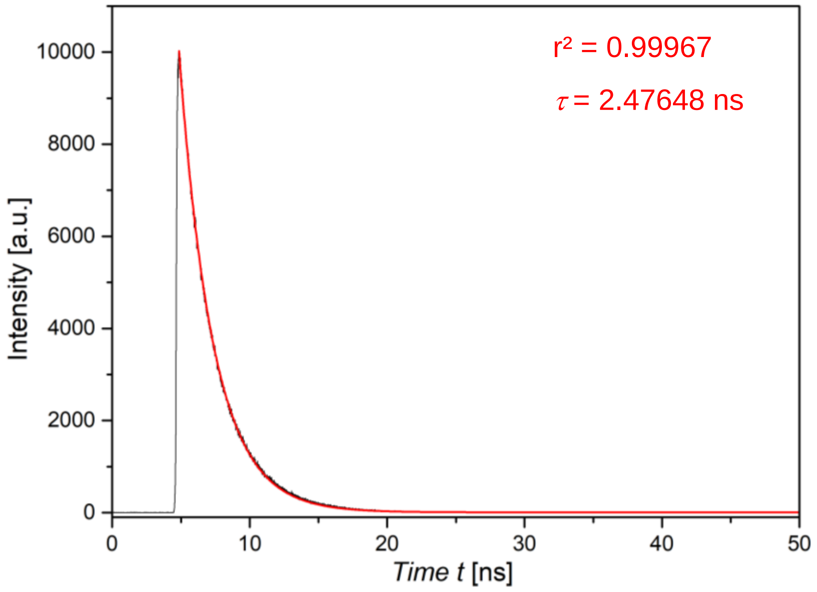

Figure S30. Exponential fit and fluorescence lifetime τ (red) of the fluorescence decay of compound **3a-aa** (CH_2Cl_2, c(**3a-aa**) = 10^{-6} M, 298 K, λ_{exc} = 641 nm, λ_{em} = 719nm).

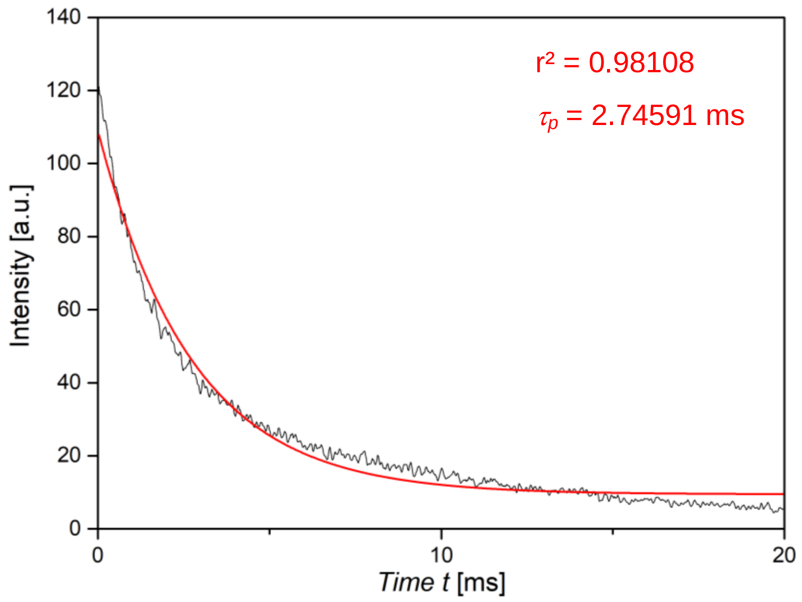

Figure S31. Exponential fit and phosphorescence lifetime τ_p (red) of the phosphorescence decay of compound **3f-ss** (toluene, c(**3f-ss**) = 10^{-6} M, 77 K, λ_{exc} = 420 nm, λ_{em} = 620 nm, degassed with N_2 for 15 min).

6 Data of Quantum Chemical Calculations

The ground state geometries of the compounds **3** and **6** were optimized using the Gaussian09 program package,[12] the PBE1PBE hybrid-functional[13] and the 6-31G** basis set.[14] The Excitation energies of compounds **3** and **6** and the excited state geometries (S_1) of **3a-aa**, **3b-ss**, **3c-aa** and **6** were calculated with TDDFT[15] methods implemented in the Gaussian09 program package using the same PBE1PBE functional[13] and either the 6-31G** or the 6-31+G** basis set[14] as indicated. The polarizable continuum model (PCM) with dichloromethane or toluene as a solvent was applied for the calculations.[16] For the calculation of redox potentials (see chapter 6.2) the ground state geometries of the radical cations and dications of compounds **3** and **6** were optimized using the Gaussian09 program package,[12] the uB3LYP functional[17] and the 6-311G* basis set[14] in the gas-phase. Then, single point calculations on the gas phase geometries was applied to determine the solvation enthalpies using the SMD solvation model with dichloromethane as a solvent.[18] All optimized geometries were confirmed as minima (NImag = 0) or as saddle points (transition states, NImag = 1) by analytical frequency analyses.

6.1 Computed xyz-Coordinates, excitations of compounds 3 and 6 and selected properties derived from the DFT-calculations

Table 3. S,N-folding angles of the S_0- and S_1-geometries ϑ and the HOMO- and LUMO-energies E_{HOMO} and E_{LUMO} derived from the optimized geometries of compounds **3** and **6** (PBE1PBE/6-31G**, PCM CH_2Cl_2).

	E_{HOMO} [eV]	E_{LUMO} [eV]	$\vartheta(S_0)$ [°]	$\vartheta(S_1)$ [°]
**3a-aa**	-5.594	-3.042	174	180
**3b-ss**	-5.622	-3.028	160	180
**6**	-5.923	-2.959	155	180
**3c-aa**	-5.133	-1.975	152	180
**3d-ss**	-5.232	-1.942	146	-
**3e-aa**	-4.829	-1.046	146	-
**3f-ss**	-4.914	-1.105	146	-

6.1.1 Compound 3a-aa

6.1.1.1 Computed xyz-Coordinates of compound 3a-aa (PBE1PBE/6-31G** PCM CH_2Cl_2)

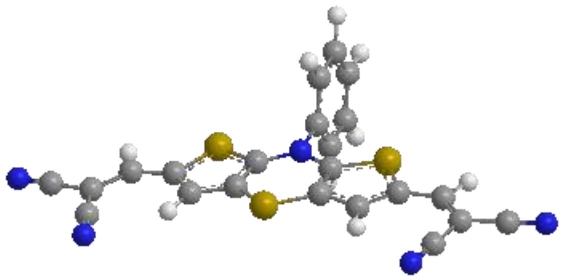

Figure S32. Optimized ground state geometry of **3a-aa** (PBE1PBE/6-31G** PCM CH_2Cl_2).

C	1.33692	-1.05961	0.17801
C	1.18717	0.32165	0.11099
N	-0.00002	1.01482	0.14157
C	-1.18724	0.32168	0.11097
C	-1.33701	-1.05958	0.17799
S	-0.00006	-2.19102	0.41776

C	2.67471	-1.45366	0.09069
C	3.56103	-0.38627	-0.02930
S	2.69003	1.13386	-0.05617
S	-2.69007	1.13391	-0.05617
C	-3.56110	-0.38620	-0.02931
C	-2.67481	-1.45360	0.09068
C	0.00002	2.45249	0.09360
C	4.97014	-0.33853	-0.12984
C	5.87500	-1.37746	-0.14731
C	7.26662	-1.09855	-0.25667
C	5.51191	-2.74879	-0.06142
C	-4.97022	-0.33845	-0.12980
C	-5.87505	-1.37739	-0.14728
C	-7.26669	-1.09852	-0.25658
C	-5.51192	-2.74872	-0.06144
N	8.40446	-0.87258	-0.34612
N	5.21105	-3.87087	0.00945
N	-8.40453	-0.87257	-0.34599
N	-5.21088	-3.87075	0.00940
C	-0.00019	3.09339	-1.14479
C	-0.00011	4.48436	-1.18181
C	0.00019	5.21447	0.00482
C	0.00040	4.56129	1.23578
C	0.00032	3.17109	1.28761
H	2.99225	-2.48851	0.12352
H	-2.99237	-2.48845	0.12351
H	5.41220	0.65202	-0.20605
H	-5.41228	0.65211	-0.20597
H	-0.00038	2.50687	-2.05833
H	-0.00028	4.99598	-2.13873
H	0.00028	6.29946	-0.02976

H 0.00062 5.13310 2.15802

H 0.00047 2.64237 2.23550

SCF Done: E(RPBE1PBE) = -2310.24984389 A.U. after 1 cycles

Zero-point correction = 0.255560 (Hartree/Particle)

Thermal correction to Energy = 0.281885

Thermal correction to Enthalpy = 0.282829

Thermal correction to Gibbs Free Energy = 0.194331

Sum of electronic and zero-point Energies = -2309.994284

Sum of electronic and thermal Energies= -2309.967959

Sum of electronic and thermal Enthalpies= -2309.967015

Sum of electronic and thermal Free Energies= -2310.055513

6.1.1.2 Computed Excitations of compound 3a-aa (PBE1PBE/6-31+G PCM CH_2Cl_2)**

Excited State 1: Singlet-A 1.9850 eV 624.61 nm f=0.9395 <S**2>=0.000

112 ->113 0.70343

This state for optimization and/or second-order correction.

Total Energy, E(TD-HF/TD-KS) = -2310.21026603

Copying the excited state density for this state as the 1-particle RhoCI density.

Excited State 2: Singlet-A 2.5981 eV 477.20 nm f=0.0504 <S**2>=0.000

111 ->113 -0.11074

112 ->114 0.69524

Excited State 3: Singlet-A 3.4792 eV 356.36 nm f=0.3642 <S**2>=0.000

110 ->113 0.69580

Excited State 4: Singlet-A 3.6245 eV 342.08 nm f=0.1856 <S**2>=0.000

111 ->113 0.69607

112 ->114 0.11187

Excited State 5: Singlet-A 3.6568 eV 339.05 nm f=0.0659 <S**2>=0.000

112 ->115 0.63301

112 ->116 -0.27741

Excited State 6: Singlet-A 3.7484 eV 330.77 nm f=0.2728 <S**2>=0.000

112 ->115 0.29296

112 ->116 0.62814

6.1.1.3 Computed xyz-Coordinates of S_1 of compound 3a-aa (PBE1PBE/6-31G** PCM CH_2Cl_2)

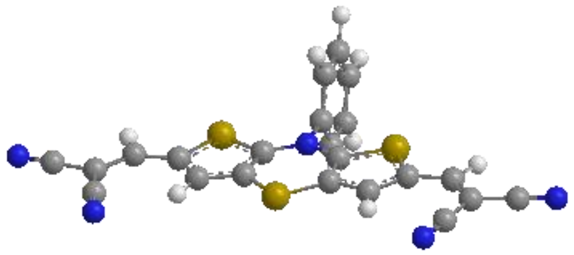

Figure S33. Optimized S_1 geometry of **3a-aa** (PBE1PBE/6-31G** PCM CH_2Cl_2).

C	-1.33248	-1.03199	-0.00001
C	-1.18407	0.36702	-0.00004
N	0.00000	1.03662	-0.00010
C	1.18407	0.36702	-0.00001
C	1.33248	-1.03199	0.00000
S	0.00000	-2.14871	0.00024
C	-2.66691	-1.44247	-0.00005
C	-3.56756	-0.37544	-0.00016
S	-2.71572	1.17835	-0.00015
S	2.71572	1.17835	-0.00014
C	3.56756	-0.37544	-0.00017
C	2.66691	-1.44247	-0.00005
C	0.00000	2.48082	0.00005
C	-4.97632	-0.35469	-0.00022
C	-5.86086	-1.42738	-0.00008
C	-7.25921	-1.18176	-0.00016

C	-5.46814	-2.78904	0.00016
C	4.97632	-0.35469	-0.00024
C	5.86086	-1.42738	-0.00010
C	7.25921	-1.18176	-0.00020
C	5.46814	-2.78904	0.00013
N	-8.40671	-0.97857	-0.00022
N	-5.14360	-3.90900	0.00038
N	8.40671	-0.97857	-0.00027
N	5.14360	-3.90900	0.00035
C	0.00016	3.15392	1.21909
C	0.00016	4.54512	1.20987
C	0.00000	5.23667	0.00031
C	-0.00016	4.54536	-1.20938
C	-0.00016	3.15415	-1.21884
H	-2.97272	-2.48061	-0.00007
H	2.97272	-2.48061	-0.00007
H	-5.44752	0.62458	-0.00038
H	5.44752	0.62458	-0.00040
H	0.00028	2.59520	2.14946
H	0.00029	5.08676	2.14997
H	0.00000	6.32206	0.00042
H	-0.00029	5.08716	-2.14938
H	-0.00028	2.59558	-2.14930

Excited State 1: Singlet-A 1.7443 eV 710.80 nm f=1.0400 <S**2>=0.000

112 ->113 -0.70428

This state for optimization and/or second-order correction.

Total Energy, E(TD-HF/TD-KS) = -2310.18282357

SCF Done: E(RPBE1PBE) = -2310.24692472 A.U. after 7 cycles

Zero-point correction = 0.253662 (Hartree/Particle)

Thermal correction to Energy = 0.280227

Thermal correction to Enthalpy = 0.281171

Thermal correction to Gibbs Free Energy = 0.192340

Sum of electronic and zero-point Energies = -2309.929161

Sum of electronic and thermal Energies = -2309.902596

Sum of electronic and thermal Enthalpies = -2309.901652

Sum of electronic and thermal Free Energies = -2309.990483

6.1.1.4 Computed Excitations of S_1 (Emission of S_1) of compound 3a-aa (PBE1PBE/6-31+G** PCM CH_2Cl_2)

Excited State 1: Singlet-A 1.8148 eV 683.18 nm f=0.8789 <S**2>=0.000

112 ->113 0.70340

This state for optimization and/or second-order correction.

Total Energy, E(TD-HF/TD-KS) = -2310.21382814

Copying the excited state density for this state as the 1-particle RhoCI density.

6.1.1.5 Computed xyz-Coordinates of radical cation of compound 3a-aa (uB3LYP/6-311G*)

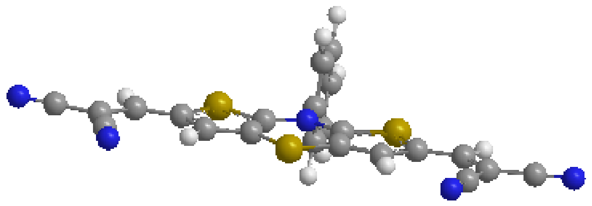

Figure S34. Optimized ground state geometry of radical cation of **3a-aa** (uB3LYP/6-311G* PCM CH_2Cl_2).

C	1.34035	-1.02030	0.33359
C	1.19413	0.34941	0.18266
N	0.00000	1.05432	0.23854
C	-1.19412	0.34941	0.18266
C	-1.34035	-1.02030	0.33359
S	0.00000	-2.10172	0.79512
C	2.66756	-1.44867	0.17370
C	3.56106	-0.41629	-0.07962
S	2.70366	1.12740	-0.16196
S	-2.70366	1.12739	-0.16196
C	-3.56106	-0.41629	-0.07962
C	-2.66756	-1.44867	0.17370
C	-0.00000	2.49773	0.17820

C	4.96980	-0.39354	-0.27236
C	5.86558	-1.43406	-0.27539
C	7.25020	-1.17203	-0.49380
C	5.48957	-2.79059	-0.06711
C	-4.96980	-0.39354	-0.27236
C	-5.86558	-1.43406	-0.27539
C	-7.25020	-1.17203	-0.49380
C	-5.48956	-2.79059	-0.06711
N	8.37073	-0.94955	-0.67169
N	5.15760	-3.88478	0.10618
N	-8.37072	-0.94955	-0.67169
N	-5.15760	-3.88478	0.10618
C	0.00001	3.13850	-1.06125
C	0.00001	4.53063	-1.10826
C	-0.00000	5.26919	0.07346
C	-0.00001	4.62050	1.30764
C	-0.00001	3.22962	1.36496
H	2.96716	-2.48430	0.25754
H	-2.96716	-2.48430	0.25754
H	5.40884	0.58514	-0.44507
H	-5.40884	0.58513	-0.44507
H	0.00002	2.55137	-1.97306
H	0.00002	5.03589	-2.06772
H	-0.00000	6.35306	0.03289
H	-0.00002	5.19637	2.22646
H	-0.00002	2.70887	2.31602

SCF Done: E(UB3LYP) = -2312.01711920 A.U. after 1 cycles

Zero-point correction= 0.251861 (Hartree/Particle)

Thermal correction to Energy= 0.278354

Thermal correction to Enthalpy= 0.279298

Thermal correction to Gibbs Free Energy= 0.190737

Sum of electronic and zero-point Energies= -2311.765258

Sum of electronic and thermal Energies= -2311.738766

Sum of electronic and thermal Enthalpies= -2311.737821

Sum of electronic and thermal Free Energies= -2311.826382

6.1.1.6 Computed xyz-Coordinates of dication (S_0) of compound 3a-aa (uB3LYP/6-311G*)

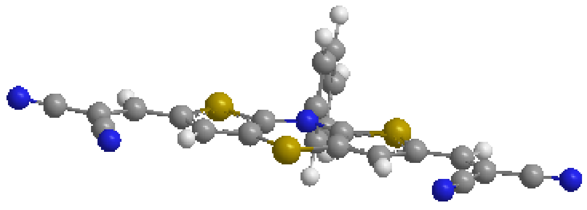

Figure S35. Optimized ground state geometry of dication of **3a-aa** (uB3LYP/6-311G* PCM CH_2Cl_2).

C	-1.33538	-1.07416	-0.00005
C	-1.18366	0.34339	0.00001
N	-0.00000	0.99781	0.00004
C	1.18366	0.34339	-0.00000
C	1.33538	-1.07416	-0.00007
S	0.00000	-2.17263	-0.00012
C	-2.67356	-1.47303	-0.00006
C	-3.55648	-0.39420	0.00000
S	-2.70919	1.16066	0.00007
S	2.70919	1.16066	0.00002
C	3.55648	-0.39420	-0.00006
C	2.67356	-1.47303	-0.00011
C	-0.00000	2.47376	0.00011
C	-4.98620	-0.33768	0.00002
C	-5.86719	-1.39029	0.00000
C	-7.26689	-1.13926	0.00003
C	-5.45817	-2.75421	-0.00004
C	4.98620	-0.33768	-0.00007
C	5.86719	-1.39029	-0.00011
C	7.26689	-1.13926	-0.00011
C	5.45817	-2.75421	-0.00014
N	-8.40421	-0.92986	0.00005
N	-5.07703	-3.84507	-0.00008
N	8.40421	-0.92986	-0.00012
N	5.07703	-3.84507	-0.00017
C	0.00004	3.13803	1.22630
C	0.00004	4.53078	1.21240
C	0.00000	5.22174	0.00024
C	-0.00004	4.53090	-1.21198

C	-0.00004	3.13815	-1.22602
H	-2.98817	-2.50745	-0.00011
H	2.98817	-2.50745	-0.00016
H	-5.43594	0.65031	0.00006
H	5.43594	0.65031	-0.00005
H	0.00007	2.59065	2.16274
H	0.00007	5.07280	2.15085
H	0.00000	6.30563	0.00029
H	-0.00007	5.07301	-2.15039
H	-0.00007	2.59086	-2.16251

SCF Done: E(UB3LYP) = -2311.62617605 A.U. after 3 cycles

Zero-point correction= 0.252140 (Hartree/Particle)

Thermal correction to Energy= 0.278626

Thermal correction to Enthalpy= 0.279570

Thermal correction to Gibbs Free Energy= 0.191478

Sum of electronic and zero-point Energies= -2311.374036

Sum of electronic and thermal Energies= -2311.347550

Sum of electronic and thermal Enthalpies= -2311.346606

Sum of electronic and thermal Free Energies= -2311.434698

6.1.1.7 Reoptimization of compound 3a-aa (uB3LYP/6-311G*)

Compound **3a-aa** in the gas phase (uB3LYP/6-311G*):

SCF Done: E(UB3LYP) = -2312.27309502 A.U. after 1 cycles

Zero-point correction= 0.252016 (Hartree/Particle)

Thermal correction to Energy= 0.278543

Thermal correction to Enthalpy= 0.279487

Thermal correction to Gibbs Free Energy= 0.191034

Sum of electronic and zero-point Energies= -2312.021079

Sum of electronic and thermal Energies= -2311.994552

Sum of electronic and thermal Enthalpies= -2311.993608

Sum of electronic and thermal Free Energies= -2312.082061

Compound **3a-aa** in CH_2Cl_2 (uB3LYP/6-311G* SMD CH_2Cl_2):

SCF Done: E(UB3LYP) = -2312.31485331 A.U. after 14 cycles

Radical cation of compound **3a-aa** in CH_2Cl_2 (uB3LYP/6-311G* SMD CH_2Cl_2):

SCF Done: E(UB3LYP) = -2312.11971867   A.U. after   20 cycles

Dication of compound **3a-aa** in CH_2Cl_2 (uB3LYP/6-311G* SMD CH_2Cl_2):

SCF Done: E(UB3LYP) = -2311.87979427   A.U. after   15 cycles

6.1.1.8 Computed xyz-Coordinates of transition state of the acceptor rotation of compound 3a-aa (PBE1PBE/6-31G** PCM CH_2Cl_2)

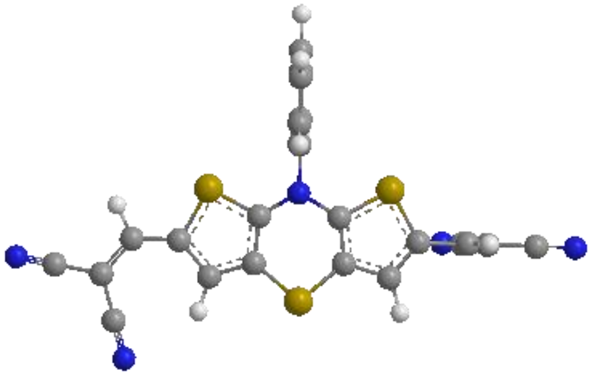

Figure S36. Optimized geometry of the transition state of the acceptor rotation of **3a-aa** (PBE1PBE/6-31G** PCM CH_2Cl_2).

C	-1.19557	-1.22448	-0.94076
C	-1.20283	0.10669	-0.61660
N	-0.08367	0.92431	-0.46540
C	1.14082	0.33005	-0.33887
C	1.43429	-1.00680	-0.61469
S	0.27349	-2.11445	-1.36520
C	-2.49843	-1.78973	-0.94115
C	-3.46986	-0.88099	-0.62290
S	-2.79954	0.69022	-0.29047
S	2.51135	1.18367	0.25179
C	3.51816	-0.24986	0.11744
C	2.76257	-1.32963	-0.34079
C	-0.24110	2.33369	-0.24017
C	-4.92102	-1.08801	-0.60076
C	-5.60123	-1.42842	0.51504
C	-7.02057	-1.62712	0.49008
C	-4.94891	-1.61457	1.77839
C	4.87821	-0.13613	0.46567

C	5.86067	-1.10783	0.47815
C	7.18430	-0.76673	0.87074
C	5.63953	-2.46321	0.11749
N	-8.16940	-1.78866	0.47390
N	-4.44322	-1.76890	2.81119
N	8.26814	-0.48894	1.19206
N	5.45420	-3.57372	-0.17985
C	-0.43283	2.80591	1.05843
C	-0.58825	4.17397	1.26366
C	-0.55155	5.05060	0.18185
C	-0.35904	4.56723	-1.11122
C	-0.20244	3.20241	-1.32924
H	-2.70800	-2.82536	-1.18153
H	3.17644	-2.31855	-0.49480
H	-5.48550	-0.96449	-1.52375
H	5.21346	0.85164	0.77362
H	-0.45882	2.10819	1.88995
H	-0.73826	4.55258	2.26957
H	-0.67354	6.11665	0.34688
H	-0.33076	5.25283	-1.95204
H	-0.05189	2.80456	-2.32778

SCF Done: E(RPBE1PBE) = -2310.23053850   A.U. after   1 cycles

Zero-point correction= 0.254929 (Hartree/Particle)

Thermal correction to Energy= 0.280670

Thermal correction to Enthalpy= 0.281614

Thermal correction to Gibbs Free Energy= 0.194935

Sum of electronic and zero-point Energies= -2309.975609

Sum of electronic and thermal Energies= -2309.949869

Sum of electronic and thermal Enthalpies= -2309.948925

Sum of electronic and thermal Free Energies= -2310.035603

6.1.2 Compound 3b-ss

6.1.2.1 Computed xyz-Coordinates of compound 3b-ss (PBE1PBE/6-31G** PCM CH_2Cl_2)

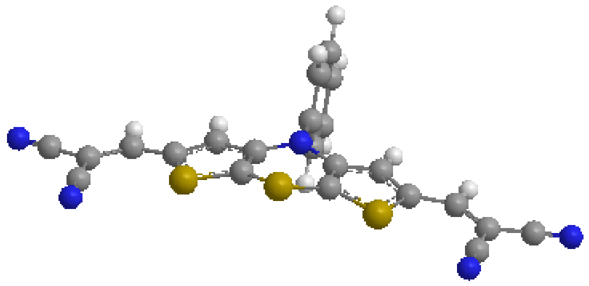

Figure S37. Optimized ground state geometry of **3b-ss** (PBE1PBE/6-31G** PCM CH_2Cl_2).

C	-1.30194	-1.21188	0.41265
C	-1.20126	0.16696	0.29710
N	-0.00012	0.86287	0.43219
C	1.20114	0.16674	0.29739
C	1.30166	-1.21207	0.41309
S	-0.00029	-2.30292	0.84477
S	-2.89807	-1.79507	0.16634
C	-3.48944	-0.17056	-0.05826
C	-2.45294	0.74984	0.03050
C	2.45280	0.74956	0.03073
C	3.48931	-0.17084	-0.05779
S	2.89780	-1.79540	0.16707
C	-4.83456	0.19617	-0.31180
C	-5.95727	-0.58327	-0.43773
C	-7.21887	0.02526	-0.69871
C	-5.94986	-2.00143	-0.32290
C	0.00014	2.29042	0.30989
C	4.83441	0.19579	-0.31151
C	5.95706	-0.58371	-0.43773
C	7.21860	0.02475	-0.69917

C	5.94960	-2.00186	-0.32292
C	0.00038	2.88755	-0.95159
C	0.00079	4.27588	-1.05230
C	0.00099	5.05953	0.10021
C	0.00078	4.45746	1.35645
C	0.00037	3.06928	1.46499
N	-5.94341	-3.16119	-0.22981
N	-8.24964	0.51956	-0.91188
N	5.94317	-3.16162	-0.22972
N	8.24920	0.51924	-0.91275
H	-2.60977	1.81523	-0.08796
H	2.60954	1.81493	-0.08805
H	-4.98817	1.26670	-0.42483
H	4.98805	1.26631	-0.42454
H	0.00024	2.26465	-1.84144
H	0.00097	4.74476	-2.03139
H	0.00132	6.14219	0.01842
H	0.00094	5.06781	2.25415
H	0.00021	2.58215	2.43511

SCF Done: E(RPBE1PBE) = -2310.24468646   A.U. after   1 cycles

Zero-point correction= 0.255568 (Hartree/Particle)

Thermal correction to Energy= 0.281827

Thermal correction to Enthalpy= 0.282772

Thermal correction to Gibbs Free Energy= 0.194805

Sum of electronic and zero-point Energies= -2309.989118

Sum of electronic and thermal Energies= -2309.962859

Sum of electronic and thermal Enthalpies= -2309.961915

Sum of electronic and thermal Free Energies= -2310.049882

6.1.2.2 Computed Excitations of compound 3b-ss (PBE1PBE/6-31G** PCM CH_2Cl_2)

Excited State 1: Singlet-A 1.9490 eV 636.14 nm f=0.4468 <S**2>=0.000

112 ->113 0.70383

This state for optimization and/or second-order correction.

Total Energy, E(TD-HF/TD-KS) = -2310.17306121

Copying the excited state density for this state as the 1-particle RhoCI density.

Excited State 2: Singlet-A 2.3499 eV 527.60 nm f=0.0200 <S**2>=0.000

112 ->114 0.69862

Excited State 3: Singlet-A 3.5472 eV 349.53 nm f=0.5038 <S**2>=0.000

110 ->113 0.69321

Excited State 4: Singlet-A 3.6622 eV 338.55 nm f=0.0102 <S**2>=0.000

110 ->114 -0.26343

111 ->113 0.64776

Excited State 5: Singlet-A 3.7851 eV 327.56 nm f=0.9112 <S**2>=0.000

111 ->114 0.68041

112 ->115 -0.14553

Excited State 6: Singlet-A 3.8547 eV 321.65 nm f=0.0571 <S**2>=0.000

110 ->114 0.64470

111 ->113

6.1.2.3 Computed xyz-Coordinates of S_1 of compound 3b-ss (PBE1PBE/6-31G** PCM CH_2Cl_2)

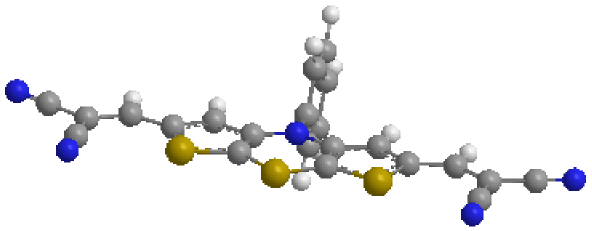

Figure S38. Optimized S_1 geometry of **3b-ss** (PBE1PBE/6-31G** PCM CH_2Cl_2).

C	-1.30459	-1.27774	0.00044
C	-1.20478	0.12726	0.00020
N	-0.00000	0.79976	0.00012
C	1.20478	0.12726	0.00021
C	1.30459	-1.27774	0.00047
S	0.00000	-2.38202	0.00073
S	-2.95437	-1.81226	0.00023
C	-3.52295	-0.14195	0.00004
C	-2.45911	0.75349	-0.00001
C	2.45911	0.75349	-0.00002
C	3.52295	-0.14195	0.00004
S	2.95437	-1.81226	0.00026
C	-4.87652	0.25291	-0.00013
C	-6.02458	-0.52216	-0.00034
C	-7.29853	0.10764	-0.00053
C	-6.03353	-1.94134	-0.00043
C	-0.00000	2.23805	0.00015
C	4.87652	0.25291	-0.00014
C	6.02458	-0.52216	-0.00036
C	7.29853	0.10764	-0.00057
C	6.03353	-1.94134	-0.00044
C	-0.00009	2.91639	-1.21566
C	-0.00009	4.30841	-1.20845

C	-0.00000	5.00154	0.00014
C	0.00009	4.30840	1.20874
C	0.00009	2.91639	1.21598
N	-6.06022	-3.10687	-0.00049
N	-8.34184	0.62545	-0.00070
N	6.06022	-3.10687	-0.00049
N	8.34184	0.62545	-0.00074
H	-2.59841	1.82690	-0.00004
H	2.59841	1.82690	-0.00006
H	-5.03063	1.32870	-0.00012
H	5.03063	1.32870	-0.00014
H	-0.00016	2.35842	-2.14666
H	-0.00016	4.84943	-2.14916
H	-0.00000	6.08702	0.00017
H	0.00016	4.84945	2.14944
H	0.00016	2.35844	2.14699

Excited State 1: Singlet-A 1.5546 eV 797.55 nm f=0.5973 <S**2>=0.000

112 ->113 -0.70526

This state for optimization and/or second-order correction.

Total Energy, E(TD-HF/TD-KS) = -2310.18317739

SCF Done: E(RPBE1PBE) = -2310.24030626 A.U. after 7 cycles

Zero-point correction= 0.253969 (Hartree/Particle)

Thermal correction to Energy= 0.280413

Thermal correction to Enthalpy= 0.281357

Thermal correction to Gibbs Free Energy= 0.193423

Sum of electronic and zero-point Energies= -2309.929208

Sum of electronic and thermal Energies= -2309.902765

Sum of electronic and thermal Enthalpies= -2309.901820

Sum of electronic and thermal Free Energies= -2309.989755

6.1.2.4 Computed Excitations of S_1 (Emission of S_1) of compound 3b-ss (PBE1PBE/6-31G** PCM CH_2Cl_2)

Excited State   1:   Singlet-A   1.6233 eV 763.79 nm f=0.4802 <S**2>=0.000

112 ->113   0.70440

This state for optimization and/or second-order correction.

Total Energy, E(TD-HF/TD-KS) = -2310.18065176

Copying the excited state density for this state as the 1-particle RhoCI density.

6.1.2.5 Computed xyz-Coordinates of radical cation of compound 3b-ss (uB3LYP/6-311G*)

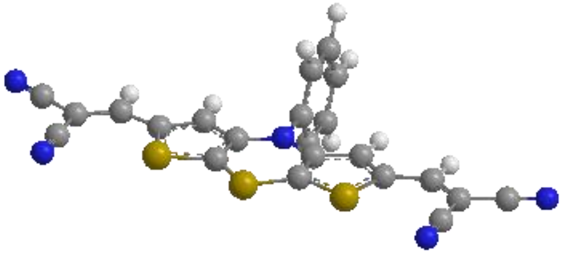

Figure S39. Optimized ground state geometry of radical cation of **3b-ss** (uB3LYP/6-311G* PCM CH_2Cl_2).

C	-1.30911	-1.22181	0.42190
C	-1.21046	0.15408	0.29478
N	0.00000	0.85588	0.43328
C	1.21047	0.15409	0.29478
C	1.30911	-1.22180	0.42189
S	0.00000	-2.30936	0.89106
S	-2.91212	-1.81600	0.16641
C	-3.50547	-0.17987	-0.07541
C	-2.46446	0.73453	0.01195
C	2.46446	0.73453	0.01195
C	3.50547	-0.17987	-0.07541
S	2.91213	-1.81599	0.16640
C	-4.85495	0.18909	-0.33774
C	-5.98361	-0.57734	-0.45824
C	-7.23738	0.04754	-0.72919
C	-5.98480	-1.99532	-0.32638
C	-0.00000	2.29103	0.32266
C	4.85496	0.18910	-0.33774

C	5.98362	-0.57733	-0.45824
C	7.23738	0.04755	-0.72918
C	5.98481	-1.99531	-0.32637
C	0.00003	2.90513	-0.93200
C	0.00003	4.29543	-1.02145
C	-0.00002	5.07003	0.13801
C	-0.00006	4.45473	1.38836
C	-0.00005	3.06421	1.48289
N	-5.97382	-3.14665	-0.21797
N	-8.24874	0.56218	-0.94891
N	5.97383	-3.14664	-0.21796
N	8.24875	0.56219	-0.94889
H	-2.61793	1.79811	-0.11373
H	2.61794	1.79811	-0.11372
H	-5.00149	1.25852	-0.46213
H	5.00150	1.25852	-0.46213
H	0.00007	2.29367	-1.82845
H	0.00005	4.77264	-1.99562
H	-0.00003	6.15242	0.06620
H	-0.00010	5.05575	2.29129
H	-0.00008	2.57080	2.44859

SCF Done: E(UB3LYP) = -2312.01387400 A.U. after 1 cycles

Zero-point correction= 0.252247 (Hartree/Particle)

Thermal correction to Energy= 0.278640

Thermal correction to Enthalpy= 0.279584

Thermal correction to Gibbs Free Energy= 0.191603

Sum of electronic and zero-point Energies= -2311.761627

Sum of electronic and thermal Energies= -2311.735234

Sum of electronic and thermal Enthalpies= -2311.734290

Sum of electronic and thermal Free Energies= -2311.822271

6.1.2.6 Computed xyz-Coordinates of dication (S_0) of compound 3b-ss (uB3LYP/6-311G*)

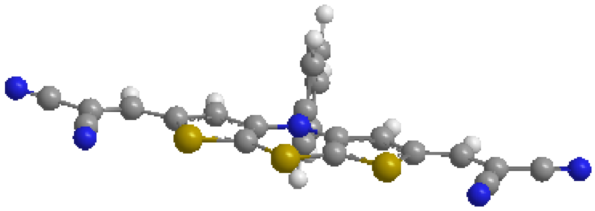

Figure S40. Optimized ground state geometry of dication of **3b-ss** (uB3LYP/6-311G* PCM CH_2Cl_2).

C	1.31118	-1.26659	-0.00004
C	1.20540	0.16045	0.00002
N	0.00000	0.81110	0.00004
C	-1.20540	0.16045	0.00002
C	-1.31118	-1.26659	-0.00003
S	-0.00000	-2.34595	-0.00008
S	2.94495	-1.82085	-0.00006
C	3.51814	-0.13714	-0.00000
C	2.47319	0.77014	0.00004
C	-2.47319	0.77014	0.00004
C	-3.51814	-0.13714	0.00001
S	-2.94495	-1.82085	-0.00005
C	4.89642	0.25084	0.00001
C	6.01234	-0.54113	-0.00003
C	7.30662	0.04927	-0.00003
C	5.94635	-1.96474	-0.00011
C	0.00000	2.28039	0.00010
C	-4.89642	0.25084	0.00002
C	-6.01234	-0.54113	-0.00001
C	-7.30662	0.04928	-0.00001
C	-5.94635	-1.96474	-0.00009
C	0.00000	2.94861	1.22265
C	0.00000	4.34261	1.21107
C	0.00000	5.03530	0.00019

C	-0.00000	4.34270	-1.21074
C	-0.00000	2.94870	-1.22241
N	5.84474	-3.11632	-0.00016
N	8.35558	0.53511	-0.00004
N	-5.84474	-3.11632	-0.00014
N	-8.35558	0.53511	-0.00001
H	2.62434	1.84040	0.00007
H	-2.62434	1.84040	0.00008
H	5.06342	1.32356	0.00005
H	-5.06342	1.32356	0.00006
H	0.00001	2.40191	2.15959
H	0.00001	4.88373	2.15021
H	0.00000	6.11918	0.00023
H	-0.00001	4.88388	-2.14983
H	-0.00001	2.40206	-2.15939

SCF Done: E(UB3LYP) = -2311.61890387 A.U. after 1 cycles

Zero-point correction= 0.252047 (Hartree/Particle)

Thermal correction to Energy= 0.278433

Thermal correction to Enthalpy= 0.279377

Thermal correction to Gibbs Free Energy= 0.191772

Sum of electronic and zero-point Energies= -2311.366857

Sum of electronic and thermal Energies= -2311.340471

Sum of electronic and thermal Enthalpies= -2311.339527

Sum of electronic and thermal Free Energies= -2311.427131

6.1.2.7 Reoptimization of compound 3b-ss (uB3LYP/6-311G*)

Compound **3b-ss** in the gas phase (uB3LYP/6-311G*):

SCF Done: E(UB3LYP) = -2312.26932403 A.U. after 1 cycles

Zero-point correction= 0.251865 (Hartree/Particle)

Thermal correction to Energy= 0.278348

Thermal correction to Enthalpy= 0.279293

Thermal correction to Gibbs Free Energy= 0.190961

Sum of electronic and zero-point Energies= -2312.017459

Sum of electronic and thermal Energies= -2311.990976

Sum of electronic and thermal Enthalpies= -2311.990031

Sum of electronic and thermal Free Energies= -2312.078363

Compound **3b-ss** in CH_2Cl_2 (uB3LYP/6-311G* SMD CH_2Cl_2):

SCF Done: E(UB3LYP) = -2312.30962381 A.U. after 15 cycles

Radical cation of compound **3b-ss** in CH_2Cl_2 (uB3LYP/6-311G* SMD CH_2Cl_2):

SCF Done: E(UB3LYP) = -2312.11729747 A.U. after 22 cycles

Dication of compound **3b-ss** in CH_2Cl_2 (uB3LYP/6-311G* SMD CH_2Cl_2):

SCF Done: E(UB3LYP) = -2311.87603765 A.U. after 16 cycles

6.1.2.8 Computed xyz-Coordinates of transition state of the acceptor rotation of compound 3b-ss (PBE1PBE/6-31G** PCM CH_2Cl_2)

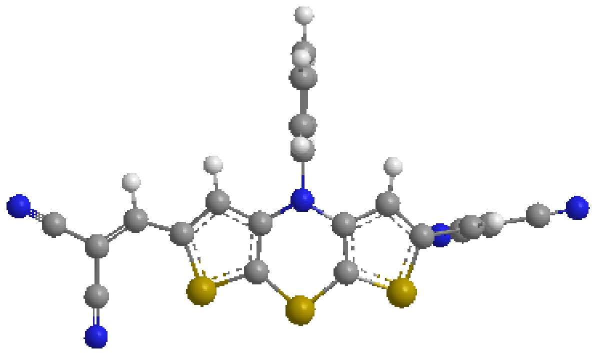

Figure S41. Optimized geometry of the transition state of the acceptor rotation of **3b-ss** (PBE1PBE/6-31G** PCM CH_2Cl_2).

C	1.34110	-1.20093	-0.73430
C	1.10657	0.15166	-0.52093
N	-0.12620	0.75778	-0.77512
C	-1.26880	-0.05132	-0.77027
C	-1.22910	-1.40403	-1.00569
S	0.20726	-2.31386	-1.47048
S	2.93109	-1.68348	-0.30254

C	3.34679	-0.04521	0.12919
C	2.25396	0.79936	-0.03116
C	-2.58877	0.40283	-0.48301
C	-3.50806	-0.61026	-0.49207
S	-2.77787	-2.14424	-0.83425
C	4.60907	0.39508	0.59432
C	5.76987	-0.30707	0.81178
C	6.92910	0.36775	1.29110
C	5.90140	-1.70512	0.58459
C	-0.26228	2.16878	-0.56903
C	-4.95696	-0.49830	-0.29710
C	-5.55535	-0.63991	0.90491
C	-6.97622	-0.51601	1.04683
C	-4.81170	-0.91255	2.10025
C	-0.43905	2.68199	0.71717
C	-0.56979	4.05632	0.89588
C	-0.52290	4.91119	-0.20382
C	-0.34487	4.39397	-1.48491
C	-0.21361	3.02015	-1.67064
N	6.01098	-2.84886	0.40009
N	7.87701	0.91651	1.68235
N	-4.23079	-1.13148	3.08045
N	-8.12595	-0.41568	1.16606
H	2.30042	1.85851	0.19241
H	-2.84230	1.43591	-0.28128
H	4.65488	1.45926	0.81346
H	-5.58773	-0.29041	-1.15976
H	-0.47456	2.00475	1.56573
H	-0.70861	4.45875	1.89459
H	-0.62513	5.98270	-0.06123
H	-0.30799	5.05938	-2.34189

H	-0.07426	2.59827	-2.66112

SCF Done: E(RPBE1PBE) = -2310.22667657 A.U. after 1 cycles

Zero-point correction= 0.254974 (Hartree/Particle)

Thermal correction to Energy= 0.280641

Thermal correction to Enthalpy= 0.281585

Thermal correction to Gibbs Free Energy= 0.195012

Sum of electronic and zero-point Energies= -2309.971703

Sum of electronic and thermal Energies= -2309.946036

Sum of electronic and thermal Enthalpies= -2309.945092

Sum of electronic and thermal Free Energies= -2310.031664

6.1.3 Compound 6

6.1.3.1 Computed xyz-Coordinates of compound 6 (PBE1PBE/6-31G** PCM CH_2Cl_2)

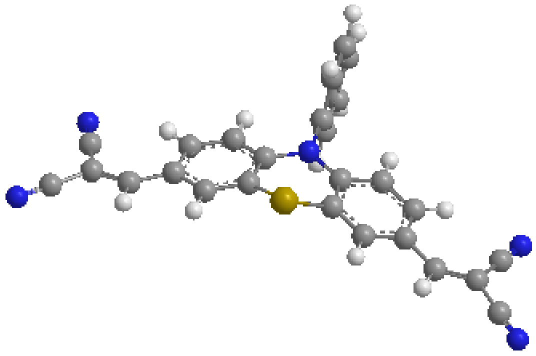

Figure S42. Optimized ground state geometry of **6** (PBE1PBE/6-31G** PCM CH_2Cl_2).

C	1.34435	-1.06771	0.58936
C	1.22712	0.30526	0.29163
N	0.00001	0.96701	0.37188
C	-1.22701	0.30515	0.29134
C	-1.34420	-1.06783	0.58904
S	0.00002	-1.95122	1.31473
C	2.55606	-1.71492	0.43546
C	3.71989	-1.02665	0.03847
C	3.60526	0.35282	-0.21075

C	2.38885	0.99607	-0.09531
C	-2.38870	0.99587	-0.09591
C	-3.60505	0.35253	-0.21157
C	-3.71964	-1.02695	0.03767
C	-2.55582	-1.71514	0.43484
C	4.92699	-1.79983	-0.07054
C	6.19444	-1.44187	-0.44315
C	7.22227	-2.43319	-0.46483
C	6.60753	-0.13297	-0.82857
C	-4.92665	-1.80022	-0.07161
C	-6.19433	-1.44208	-0.44327
C	-7.22196	-2.43360	-0.46562
C	-6.60788	-0.13281	-0.82691
C	-0.00007	2.40376	0.27530
C	-0.00034	3.15037	1.44985
C	-0.00046	4.54094	1.37569
C	-0.00032	5.17452	0.13551
C	-0.00005	4.41972	-1.03616
C	0.00007	3.02954	-0.97116
N	8.06133	-3.23757	-0.48339
N	6.97203	0.92415	-1.14768
N	-8.06087	-3.23813	-0.48475
N	-6.97285	0.92460	-1.14451
H	2.61219	-2.77815	0.65255
H	4.46655	0.93779	-0.50834
H	2.33291	2.05604	-0.30819
H	-2.33277	2.05581	-0.30888
H	-4.46629	0.93741	-0.50945
H	-2.61190	-2.77839	0.65185
H	4.81686	-2.85172	0.18279
H	-4.81624	-2.85233	0.18066

H	-0.00046	2.63991	2.40785
H	-0.00067	5.12777	2.28883
H	-0.00042	6.25875	0.08074
H	0.00006	4.91226	-2.00341
H	0.00030	2.42924	-1.87616

SCF Done: E(RPBE1PBE) = -1668.86050563 A.U. after 1 cycles

Zero-point correction= 0.323283 (Hartree/Particle)

Thermal correction to Energy= 0.349905

Thermal correction to Enthalpy= 0.350849

Thermal correction to Gibbs Free Energy= 0.262578

Sum of electronic and zero-point Energies= -1668.537223

Sum of electronic and thermal Energies= -1668.510601

Sum of electronic and thermal Enthalpies= -1668.509657

Sum of electronic and thermal Free Energies= -1668.597927

6.1.3.2 Computed Excitations of compound 6 (PBE1PBE/6-31G PCM CH_2Cl_2)**

Excited State 1: Singlet-A 2.3171 eV 535.09 nm f=0.6349 <S**2>=0.000
110 ->111 0.70086
This state for optimization and/or second-order correction.
Total Energy, E(TD-HF/TD-KS) = -1668.77535535
Copying the excited state density for this state as the 1-particle RhoCI density.

Excited State 2: Singlet-A 2.9458 eV 420.89 nm f=0.0706 <S**2>=0.000
110 ->112 0.69417

Excited State 3: Singlet-A 3.4187 eV 362.66 nm f=0.7812 <S**2>=0.000
109 ->111 0.68747
110 ->113 -0.10821

Excited State 4: Singlet-A 3.9214 eV 316.17 nm f=0.0001 <S**2>=0.000
105 ->111 -0.14632
108 ->111 -0.33620
109 ->112 0.57254
110 ->114 -0.15126

Excited State 5: Singlet-A 3.9694 eV 312.35 nm f=0.1125 <S**2>=0.000

106 ->111 0.17976

108 ->111 0.59325

109 ->112 0.26083

110 ->112 -0.10455

110 ->114 -0.14412

Excited State 6: Singlet-A 4.0241 eV 308.11 nm f=0.7064 <S**2>=0.000

108 ->112 0.12274

110 ->113 0.65752

110 ->116 -0.13343

6.1.3.3 Computed xyz-Coordinates of S_1 of compound 6 (PBE1PBE/6-31G** PCM CH_2Cl_2)

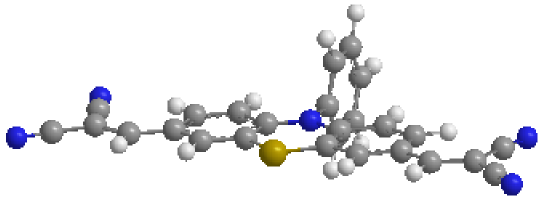

Figure S43. Optimized S_1 geometry of **6** (PBE1PBE/6-31G** PCM CH_2Cl_2).

C	1.35341	-1.18297	0.00008
C	1.22552	0.23836	-0.00005
N	0.00000	0.86876	-0.00007
C	-1.22551	0.23836	-0.00005
C	-1.35341	-1.18297	0.00008
S	-0.00000	-2.25870	0.00010
C	2.60542	-1.78663	0.00015
C	3.79182	-1.03831	0.00005
C	3.65634	0.38328	-0.00009
C	2.42707	0.98748	-0.00014
C	-2.42706	0.98748	-0.00013
C	-3.65633	0.38328	-0.00009
C	-3.79182	-1.03831	0.00005
C	-2.60542	-1.78663	0.00015

C	5.02119	-1.76167	0.00008
C	6.33102	-1.30803	-0.00004
C	7.39124	-2.25654	-0.00003
C	6.74426	0.05025	-0.00017
C	-5.02119	-1.76166	0.00008
C	-6.33102	-1.30803	-0.00004
C	-7.39124	-2.25654	-0.00003
C	-6.74426	0.05025	-0.00017
C	0.00000	2.31424	0.00001
C	0.00000	2.99143	1.21527
C	0.00000	4.38343	1.20835
C	-0.00000	5.07730	0.00023
C	-0.00000	4.38361	-1.20801
C	-0.00000	2.99162	-1.21514
N	8.25839	-3.03462	-0.00005
N	7.11530	1.15551	-0.00028
N	-8.25838	-3.03462	-0.00005
N	-7.11532	1.15551	-0.00028
H	2.66189	-2.87210	0.00031
H	4.53600	1.01424	-0.00016
H	2.37362	2.06866	-0.00024
H	-2.37361	2.06866	-0.00023
H	-4.53599	1.01424	-0.00015
H	-2.66189	-2.87210	0.00030
H	4.92029	-2.84354	0.00018
H	-4.92029	-2.84353	0.00018
H	0.00000	2.43371	2.14640
H	0.00000	4.92386	2.14940
H	-0.00000	6.16272	0.00030
H	-0.00000	4.92421	-2.14896
H	-0.00000	2.43402	-2.14635

Excited State 1: Singlet-A 1.8263 eV 678.88 nm f=0.6197 <S**2>=0.000

110 ->111 -0.70375

This state for optimization and/or second-order correction.

Total Energy, E(TD-HF/TD-KS) = -1668.78756592

SCF Done: E(RPBE1PBE) = -1668.85468164 A.U. after 7 cycles

Zero-point correction= 0.320970 (Hartree/Particle)

Thermal correction to Energy= 0.347895

Thermal correction to Enthalpy= 0.348839

Thermal correction to Gibbs Free Energy= 0.259869

Sum of electronic and zero-point Energies= -1668.466595

Sum of electronic and thermal Energies= -1668.439671

Sum of electronic and thermal Enthalpies= -1668.438727

Sum of electronic and thermal Free Energies= -1668.527697

6.1.3.4 Computed Excitations of S_1 (Emission of S_1) of compound 6 (PBE1PBE/6-31G** PCM CH_2Cl_2)

Excited State 1: Singlet-A 1.8815 eV 658.97 nm f=0.4870 <S**2>=0.000

110 ->111 0.70200

This state for optimization and/or second-order correction.

Total Energy, E(TD-HF/TD-KS) = -1668.78553875

Copying the excited state density for this state as the 1-particle RhoCI density.

6.1.3.5 Computed xyz-Coordinates of radical cation of compound 6 (uB3LYP/6-311G*)

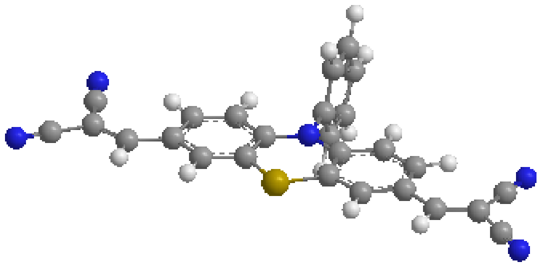

Figure S44. Optimized ground state geometry of radical cation of **6** (uB3LYP/6-311G* PCM CH_2Cl_2).

C	1.36277	-1.19406	-0.00008
C	1.23436	0.22061	-0.00017
N	-0.00003	0.86200	-0.00016

C	-1.23439	0.22056	-0.00017
C	-1.36274	-1.19412	-0.00009
S	0.00004	-2.28487	-0.00016
C	2.62476	-1.78860	-0.00001
C	3.80022	-1.03199	-0.00006
C	3.66452	0.38062	-0.00021
C	2.42819	0.98005	-0.00026
C	-2.42826	0.97995	-0.00026
C	-3.66455	0.38048	-0.00022
C	-3.80019	-1.03214	-0.00006
C	-2.62471	-1.78871	-0.00002
C	5.05727	-1.76048	0.00005
C	6.34315	-1.31140	0.00011
C	7.41399	-2.25929	0.00018
C	6.73175	0.06287	0.00008
C	-5.05723	-1.76064	0.00004
C	-6.34308	-1.31148	0.00010
C	-7.41399	-2.25929	0.00018
C	-6.73155	0.06283	0.00009
C	-0.00007	2.32436	-0.00003
C	-0.00009	3.00159	1.21633
C	-0.00013	4.39510	1.20929
C	-0.00014	5.08951	0.00026
C	-0.00012	4.39536	-1.20892
C	-0.00009	3.00185	-1.21624
N	8.27334	-3.03054	0.00026
N	7.04193	1.17536	0.00005
N	-8.27340	-3.03047	0.00025
N	-7.04149	1.17539	0.00007
H	2.68882	-2.87200	0.00009
H	4.53625	1.01881	-0.00030

H	2.37233	2.05842	-0.00035
H	-2.37244	2.05833	-0.00036
H	-4.53633	1.01861	-0.00032
H	-2.68873	-2.87211	0.00009
H	4.95537	-2.84123	0.00010
H	-4.95537	-2.84139	0.00009
H	-0.00008	2.45119	2.15091
H	-0.00015	4.93518	2.14910
H	-0.00017	6.17353	0.00037
H	-0.00013	4.93563	-2.14862
H	-0.00007	2.45163	-2.15093

SCF Done: E(UB3LYP) = -1670.48252963 A.U. after 1 cycles

Zero-point correction= 0.319252 (Hartree/Particle)

Thermal correction to Energy= 0.346172

Thermal correction to Enthalpy= 0.347117

Thermal correction to Gibbs Free Energy= 0.257395

Sum of electronic and zero-point Energies= -1670.163277

Sum of electronic and thermal Energies= -1670.136357

Sum of electronic and thermal Enthalpies= -1670.135413

Sum of electronic and thermal Free Energies= -1670.225134

6.1.3.6 Reoptimization of compound 6 (uB3LYP/6-311G*)

Compound **4** in the gas phase (uB3LYP/6-311G*):

SCF Done: E(UB3LYP) = -1670.74815348 A.U. after 1 cycles

Zero-point correction= 0.319327 (Hartree/Particle)

Thermal correction to Energy= 0.346086

Thermal correction to Enthalpy= 0.347030

Thermal correction to Gibbs Free Energy= 0.258537

Sum of electronic and zero-point Energies= -1670.428826

Sum of electronic and thermal Energies= -1670.402068

Sum of electronic and thermal Enthalpies= -1670.401124

Sum of electronic and thermal Free Energies= -1670.489617

Compound **6** in CH_2Cl_2 (uB3LYP/6-311G* SMD CH_2Cl_2):

SCF Done: E(UB3LYP) = -1670.79174203   A.U. after   14 cycles

Radical cation of compound **6** in CH_2Cl_2 (uB3LYP/6-311G* SMD CH_2Cl_2):

SCF Done: E(UB3LYP) = -1670.59154871   A.U. after   20 cycles

6.1.4 Compound 3c-aa

6.1.4.1 Computed xyz-Coordinates of compound 3c-aa (PBE1PBE/6-31G** PCM CH_2Cl_2)

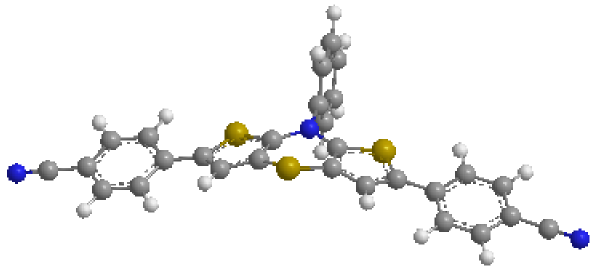

Figure S45. Optimized ground state geometry of **3c-aa** (PBE1PBE/6-31G** PCM CH_2Cl_2).

C	1.32379	-1.16032	0.84709
C	1.18286	0.16631	0.51325
N	-0.00007	0.89044	0.55092
C	-1.18305	0.16636	0.51276
C	-1.32398	-1.16028	0.84662
S	-0.00023	-2.10214	1.55942
S	-2.66339	0.83605	-0.08458
C	-3.50243	-0.67874	0.15867
C	-2.63770	-1.63793	0.63223
C	2.63742	-1.63808	0.63239
C	3.50210	-0.67899	0.15852
S	2.66314	0.83585	-0.08471
C	-4.92619	-0.79895	-0.11799
C	4.92585	-0.79929	-0.11821
C	0.00028	2.30459	0.31856

C	5.75109	0.33269	-0.23949
C	7.10429	0.20849	-0.50128
C	7.67521	-1.06363	-0.64329
C	6.86608	-2.20358	-0.52525
C	5.51302	-2.06885	-0.27369
C	0.00413	3.17280	1.40887
C	0.00468	4.54606	1.18607
C	0.00128	5.04290	-0.11621
C	-0.00252	4.16964	-1.20124
C	-0.00292	2.79359	-0.98856
C	-5.51341	-2.06847	-0.27363
C	-6.86650	-2.20312	-0.52508
C	-7.67560	-1.06313	-0.64286
C	-7.10464	0.20895	-0.50070
C	-5.75141	0.33307	-0.23900
C	9.07062	-1.19833	-0.91024
C	-9.07104	-1.19775	-0.90970
N	10.20777	-1.30816	-1.12743
N	-10.20822	-1.30752	-1.12681
H	-2.94179	-2.65089	0.86959
H	2.94152	-2.65101	0.86983
H	5.33278	1.32679	-0.11062
H	7.72600	1.09314	-0.58837
H	7.30223	-3.18989	-0.64315
H	4.89847	-2.96065	-0.21389
H	0.00659	2.76531	2.41484
H	0.00766	5.22829	2.03035
H	0.00165	6.11535	-0.28553
H	-0.00504	4.55722	-2.21507
H	-0.00557	2.09958	-1.82381
H	-4.89887	-2.96030	-0.21404

H -7.30269 -3.18940 -0.64310

H -7.72633 1.09363 -0.58759

H -5.33306 1.32714 -0.11000

SCF Done: E(RPBE1PBE) = -2432.94264178 A.U. after 1 cycles

Zero-point correction= 0.353385 (Hartree/Particle)

Thermal correction to Energy= 0.381469

Thermal correction to Enthalpy= 0.382413

Thermal correction to Gibbs Free Energy= 0.289619

Sum of electronic and zero-point Energies= -2432.589257

Sum of electronic and thermal Energies= -2432.561173

Sum of electronic and thermal Enthalpies= -2432.560228

Sum of electronic and thermal Free Energies= -2432.653022

6.1.4.2 Computed Excitations of compound 3c-aa (PBE1PBE/6-31G PCM CH_2Cl_2)**

Excited State 1: Singlet-A 2.4700 eV 501.97 nm f=0.8492 <S**2>=0.000

126 ->127 0.69692

This state for optimization and/or second-order correction.

Total Energy, E(TD-HF/TD-KS) = -2432.85187213

Copying the excited state density for this state as the 1-particle RhoCI density.

Excited State 2: Singlet-A 2.9356 eV 422.35 nm f=0.0386 <S**2>=0.000

126 ->128 0.69990

Excited State 3: Singlet-A 3.5783 eV 346.49 nm f=0.0108 <S**2>=0.000

126 ->129 0.67744

126 ->133 -0.11186

Excited State 4: Singlet-A 3.7554 eV 330.15 nm f=0.1396 <S**2>=0.000

126 ->129 -0.18102

126 ->130 -0.19049

126 ->132 0.43547

126 ->133 -0.43062

126 ->136 -0.18227

Excited State 5: Singlet-A 3.8315 eV 323.59 nm f=0.0004 <S**2>=0.000

126 ->130 0.58277

126 ->132 0.36341

126 ->133 0.11394

Excited State 6: Singlet-A 3.9123 eV 316.91 nm f=0.0020 <S**2>=0.000

126 ->131 0.66413

126 ->134 0.13740

126 ->135 0.14445

Excited State 7: Singlet-A 4.0019 eV 309.81 nm f=0.0020 <S**2>=0.000

126 ->130 -0.30615

126 ->132 0.38578

126 ->133 0.47525

126 ->136 0.10268

Excited State 8: Singlet-A 4.0408 eV 306.83 nm f=0.0626 <S**2>=0.000

125 ->127 0.69692

Excited State 9: Singlet-A 4.1488 eV 298.84 nm f=0.9124 <S**2>=0.000

124 ->127 0.64158

125 ->128 0.26370

Excited State 10: Singlet-A 4.2850 eV 289.34 nm f=0.2203 <S**2>=0.000

124 ->127 -0.26410

125 ->128 0.64170

6.1.4.3 Computed xyz-Coordinates of S_1 of compound 3c-aa (PBE1PBE/6-31G** PCM CH_2Cl_2)

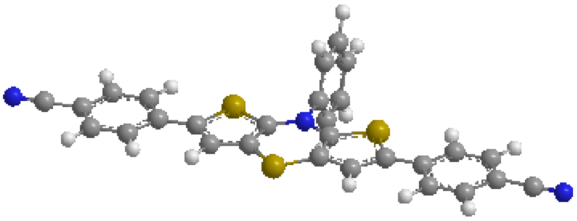

Figure S46. Optimized S_1 geometry of **3c-aa** (PBE1PBE/6-31G** PCM CH_2Cl_2).

C	1.18487	0.03778	-0.00013
C	1.33244	-1.35801	-0.00014
S	0.00000	-2.47476	-0.00035
C	-1.33244	-1.35801	-0.00013
C	-1.18487	0.03778	-0.00011
N	-0.00000	0.71205	-0.00009
C	-2.67413	-1.76363	-0.00008
C	-3.57464	-0.70898	-0.00001
S	-2.72752	0.84641	-0.00002
S	2.72752	0.84641	-0.00003
C	3.57464	-0.70898	-0.00002
C	2.67413	-1.76363	-0.00009
C	5.01064	-0.75340	0.00002
C	-5.01064	-0.75340	0.00002
C	-0.00000	2.15326	-0.00003
C	5.70148	-1.99080	0.00019
C	7.07807	-2.04205	0.00023
C	7.83647	-0.85297	0.00011
C	7.16728	0.38477	-0.00005
C	5.78857	0.42963	-0.00010
C	-5.78857	0.42963	-0.00011
C	-7.16728	0.38476	-0.00007
C	-7.83647	-0.85297	0.00010

C	-7.07807	-2.04205	0.00023
C	-5.70148	-1.99080	0.00020
C	0.00006	2.82952	-1.21735
C	0.00006	4.22090	-1.20908
C	-0.00000	4.91304	0.00010
C	-0.00006	4.22078	1.20922
C	-0.00006	2.82940	1.21735
C	9.25614	-0.90366	0.00016
C	-9.25614	-0.90366	0.00015
N	10.42099	-0.94579	0.00022
N	-10.42099	-0.94579	0.00020
H	-2.97390	-2.80419	-0.00008
H	2.97390	-2.80419	-0.00008
H	5.14712	-2.92319	0.00033
H	7.58471	-3.00169	0.00037
H	7.74037	1.30620	-0.00015
H	5.30262	1.40157	-0.00025
H	-5.30262	1.40157	-0.00028
H	-7.74037	1.30620	-0.00018
H	-7.58471	-3.00169	0.00038
H	-5.14712	-2.92319	0.00035
H	0.00010	2.27081	-2.14774
H	0.00010	4.76249	-2.14938
H	-0.00000	5.99854	0.00015
H	-0.00010	4.76228	2.14957
H	-0.00010	2.27060	2.14768

Excited State 1: Singlet-A 1.8876 eV 656.82 nm f=1.0361 <S**2>=0.000

126 ->127 0.70022

This state for optimization and/or second-order correction.

Total Energy, E(TD-HF/TD-KS) = -2432.86685164

SCF Done: E(RPBE1PBE) = -2432.93622076 A.U. after 6 cycles

Zero-point correction= 0.350865 (Hartree/Particle)

Thermal correction to Energy= 0.379450

Thermal correction to Enthalpy= 0.380394

Thermal correction to Gibbs Free Energy= 0.285526

Sum of electronic and zero-point Energies= -2432.515986

Sum of electronic and thermal Energies= -2432.487402

Sum of electronic and thermal Enthalpies= -2432.486457

Sum of electronic and thermal Free Energies= -2432.581325

6.1.4.4 Computed Excitations of S_1 (Emission of S_1) of compound 3c-aa (PBE1PBE/6-31G PCM CH_2Cl_2)**

Excited State 1: Singlet-A 1.9594 eV 632.75 nm f=0.8906 <S**2>=0.000

126 ->127 0.69905

This state for optimization and/or second-order correction.

Total Energy, E(TD-HF/TD-KS) = -2432.86421280

Copying the excited state density for this state as the 1-particle RhoCI density.

6.1.4.5 Computed xyz-Coordinates of radical cation of compound 3c-aa (uB3LYP/6-311G*)

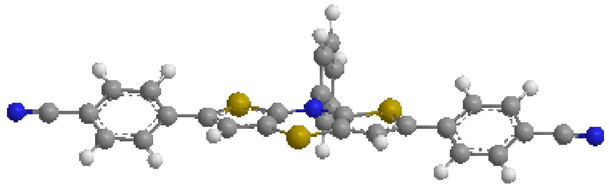

Figure S47. Optimized ground state geometry of radical cation of **3c-aa** (uB3LYP/6-311G* PCM CH_2Cl_2).

C	1.19022	0.03002	0.00398
C	1.33840	-1.35429	-0.06948
S	-0.00003	-2.48293	-0.14752
C	-1.33843	-1.35427	-0.06916
C	-1.19022	0.03004	0.00407
N	0.00001	0.71583	0.03557
C	-2.69358	-1.75468	-0.09299
C	-3.57788	-0.70249	-0.03558

S	-2.72961	0.83958	0.06256
S	2.72963	0.83949	0.06260
C	3.57786	-0.70253	-0.03586
C	2.69354	-1.75471	-0.09341
C	5.03739	-0.74389	-0.03556
C	-5.03743	-0.74383	-0.03532
C	0.00003	2.17039	0.10248
C	5.70808	-1.87871	0.45599
C	7.09143	-1.94108	0.45197
C	7.84288	-0.86325	-0.04052
C	7.18493	0.27520	-0.52799
C	5.80066	0.33060	-0.52496
C	-5.80064	0.33059	-0.52494
C	-7.18491	0.27517	-0.52812
C	-7.84291	-0.86321	-0.04057
C	-7.09151	-1.94095	0.45219
C	-5.70815	-1.87857	0.45634
C	0.00004	2.90178	-1.08392
C	0.00006	4.29235	-1.01273
C	0.00008	4.92948	0.22754
C	0.00007	4.18284	1.40490
C	0.00005	2.79131	1.35019
C	9.27075	-0.92376	-0.04241
C	-9.27078	-0.92376	-0.04265
N	10.42438	-0.97232	-0.04405
N	-10.42440	-0.97238	-0.04442
H	-3.00722	-2.78636	-0.18489
H	3.00717	-2.78635	-0.18577
H	5.14496	-2.70610	0.87200
H	7.59875	-2.81656	0.83900
H	7.76320	1.10574	-0.91435

H	5.31216	1.20806	-0.93519
H	-5.31211	1.20804	-0.93516
H	-7.76314	1.10565	-0.91468
H	-7.59885	-2.81637	0.83933
H	-5.14507	-2.70591	0.87252
H	0.00003	2.39217	-2.04110
H	0.00007	4.87580	-1.92626
H	0.00010	6.01254	0.27671
H	0.00008	4.68114	2.36746
H	0.00003	2.19765	2.25766

SCF Done: E(UB3LYP) = -2434.89272424 A.U. after 1 cycles

Zero-point correction= 0.349194 (Hartree/Particle)

Thermal correction to Energy= 0.377344

Thermal correction to Enthalpy= 0.378288

Thermal correction to Gibbs Free Energy= 0.285687

Sum of electronic and zero-point Energies= -2434.543531

Sum of electronic and thermal Energies= -2434.515381

Sum of electronic and thermal Enthalpies= -2434.514436

Sum of electronic and thermal Free Energies= -2434.607037

6.1.4.6 Computed xyz-Coordinates of dication (S_0) of compound 3c-aa (uB3LYP/6-311G*)

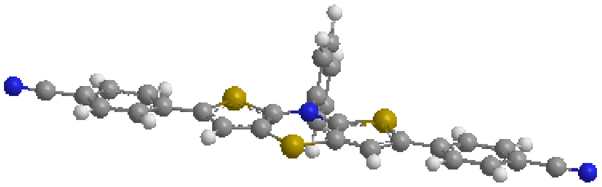

Figure S48. Optimized ground state geometry of dication of **3c-aa** (uB3LYP/6-311G* PCM CH_2Cl_2).

C	1.18528	0.02714	0.00261
C	1.34075	-1.38292	-0.02104
S	0.00002	-2.48759	-0.04675
C	-1.34072	-1.38294	-0.02129

C	-1.18527	0.02713	0.00253
N	-0.00000	0.68162	0.01118
C	-2.67904	-1.77202	-0.02892
C	-3.57119	-0.69995	-0.00972
S	-2.71507	0.84793	0.02379
S	2.71508	0.84796	0.02394
C	3.57119	-0.69989	-0.00959
C	2.67909	-1.77199	-0.02865
C	5.01065	-0.74033	-0.01066
C	-5.01063	-0.74039	-0.01071
C	-0.00001	2.15443	0.03116
C	5.69018	-1.97629	0.12735
C	7.06851	-2.02840	0.12729
C	7.81765	-0.84483	-0.01351
C	7.15907	0.39186	-0.15184
C	5.78074	0.43965	-0.14868
C	-5.78075	0.43987	-0.14631
C	-7.15907	0.39213	-0.14942
C	-7.81767	-0.84483	-0.01350
C	-7.06853	-2.02869	0.12486
C	-5.69020	-1.97661	0.12492
C	0.00010	2.83702	-1.18386
C	0.00012	4.22951	-1.15291
C	-0.00001	4.90459	0.06789
C	-0.00015	4.19723	1.27015
C	-0.00013	2.80433	1.26395
C	9.24088	-0.89744	-0.01519
C	-9.24089	-0.89741	-0.01516
N	10.39563	-0.93932	-0.01664
N	-10.39565	-0.93926	-0.01653
H	-2.99454	-2.80572	-0.06298

H	2.99463	-2.80567	-0.06296
H	5.13971	-2.90030	0.25204
H	7.58007	-2.97650	0.23896
H	7.73934	1.29941	-0.26356
H	5.30058	1.40500	-0.27048
H	-5.30060	1.40548	-0.26603
H	-7.73933	1.29992	-0.25921
H	-7.58010	-2.97700	0.23462
H	-5.13978	-2.90092	0.24755
H	0.00021	2.30056	-2.12643
H	0.00025	4.78391	-2.08411
H	-0.00001	5.98842	0.08236
H	-0.00028	4.72653	2.21583
H	-0.00024	2.24308	2.19200

SCF Done: E(UB3LYP) = -2434.53501764 A.U. after 1 cycles

Zero-point correction= 0.349368 (Hartree/Particle)

Thermal correction to Energy= 0.377619

Thermal correction to Enthalpy= 0.378564

Thermal correction to Gibbs Free Energy= 0.285155

Sum of electronic and zero-point Energies= -2434.185649

Sum of electronic and thermal Energies= -2434.157398

Sum of electronic and thermal Enthalpies= -2434.156454

Sum of electronic and thermal Free Energies= -2434.249863

6.1.4.7 Reoptimization of compound 3c-aa (uB3LYP/6-311G*)

Compound **3c-aa** in the gas phase (uB3LYP/6-311G*):

SCF Done: E(UB3LYP) = -2435.11980901 A.U. after 1 cycles

Zero-point correction= 0.348525 (Hartree/Particle)

hermal correction to Energy= 0.376847

Thermal correction to Enthalpy= 0.377791

Thermal correction to Gibbs Free Energy= 0.284413

Sum of electronic and zero-point Energies= -2434.771284

Sum of electronic and thermal Energies= -2434.742963

Sum of electronic and thermal Enthalpies= -2434.742018

Sum of electronic and thermal Free Energies= -2434.835396

Compound **3c-aa** in CH_2Cl_2 (uB3LYP/6-311G* SMD CH_2Cl_2):

SCF Done: E(UB3LYP) = -2435.16524136 A.U. after 14 cycles

Radical cation of compound **3c-aa** in CH_2Cl_2 (uB3LYP/6-311G* SMD CH_2Cl_2):

SCF Done: E(UB3LYP) = -2434.99326328 A.U. after 20 cycles

Dication of compound **3c-aa** in CH_2Cl_2 (uB3LYP/6-311G* SMD CH_2Cl_2):

SCF Done: E(UB3LYP) = -2434.77599913 A.U. after 15 cycles

6.1.4.8 Computed xyz-Coordinates of transition state of the benzonitrile rotation of compound 3c-aa (PBE1PBE/6-31G** PCM CH_2Cl_2)

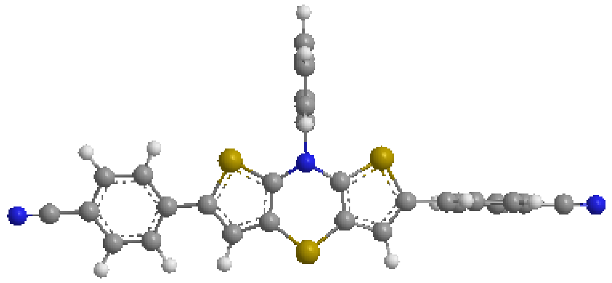

Figure S49. Optimized geometry of the transition state of the benzonitrile rotation of **3c-aa** (PBE1PBE/6-31G** PCM CH_2Cl_2).

C	1.32479	-1.08226	1.00131
C	1.18373	0.21698	0.59025
N	-0.00332	0.94934	0.61206
C	-1.18087	0.21883	0.57722
C	-1.32055	-1.09025	0.97723
S	-0.00225	-1.96898	1.77707
S	-2.65128	0.84009	-0.09517
C	-3.48398	-0.66891	0.20630
C	-2.62303	-1.59297	0.75164

C	2.63369	-1.59280	0.77303
C	3.47664	-0.67701	0.20997
S	2.65371	0.82912	-0.09966
C	-4.89883	-0.81599	-0.09925
C	4.91121	-0.82369	-0.11384
C	-0.00011	2.34743	0.30097
C	5.88812	-0.51671	0.84112
C	7.23598	-0.66637	0.54806
C	7.62018	-1.12651	-0.71795
C	6.65279	-1.43383	-1.68297
C	5.30830	-1.27882	-1.37682
C	0.00756	3.27672	1.33981
C	0.01197	4.63557	1.04054
C	0.00871	5.05881	-0.28739
C	0.00133	4.12576	-1.32138
C	-0.00268	2.76390	-1.03139
C	-5.47410	-2.09662	-0.20173
C	-6.81897	-2.25521	-0.48145
C	-7.63236	-1.12976	-0.68179
C	-7.07334	0.15254	-0.59293
C	-5.72850	0.30113	-0.30301
C	9.00814	-1.28341	-1.02715
C	-9.01904	-1.28951	-0.97803
N	10.13538	-1.41083	-1.27861
N	-10.14928	-1.41962	-1.21919
H	-2.92385	-2.59541	1.03394
H	2.94429	-2.59633	1.04072
H	5.58390	-0.16187	1.82058
H	7.99039	-0.43092	1.29105
H	6.95724	-1.79031	-2.66115
H	4.55379	-1.51601	-2.11992

H	0.00962	2.92614	2.36702
H	0.01771	5.36404	1.84531
H	0.01204	6.12009	-0.51667
H	-0.00088	4.45584	-2.35544
H	-0.00784	2.02485	-1.82707
H	-4.85572	-2.97907	-0.07753
H	-7.24534	-3.24988	-0.55795
H	-7.69801	1.02654	-0.74358
H	-5.32033	1.30404	-0.21737

SCF Done: E(RPBE1PBE) = -2432.93539748   A.U. after   1 cycles

Zero-point correction= 0.353210 (Hartree/Particle)

Thermal correction to Energy= 0.380485

Thermal correction to Enthalpy= 0.381429

Thermal correction to Gibbs Free Energy= 0.291012

Sum of electronic and zero-point Energies= -2432.582188

Sum of electronic and thermal Energies= -2432.554913

Sum of electronic and thermal Enthalpies= -2432.553969

Sum of electronic and thermal Free Energies= -2432.644386

6.1.5 Compound 3d-ss

6.1.5.1 Computed xyz-Coordinates of compound 3d-ss (PBE1PBE/6-31G** PCM CH_2Cl_2)

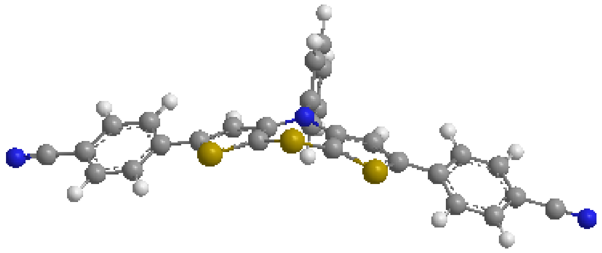

Figure S50. Optimized ground state geometry of **3d-ss** (PBE1PBE/6-31G** PCM CH_2Cl_2).

C	1.29144	-1.63881	0.88887
C	1.19808	-0.27477	0.72448
N	0.00009	0.42587	0.92421

C	-1.19792	-0.27482	0.72471
C	-1.29129	-1.63885	0.88918
S	0.00016	-2.65524	1.52093
C	-2.42415	0.29563	0.29013
C	-3.42729	-0.62982	0.12324
S	-2.85646	-2.23378	0.47722
S	2.85661	-2.23375	0.47691
C	3.42748	-0.62979	0.12314
C	2.42437	0.29569	0.29004
C	-0.00013	1.85367	0.82677
C	4.80451	-0.39309	-0.29730
C	-4.80430	-0.39318	-0.29731
C	5.38073	0.88007	-0.14544
C	6.67946	1.12687	-0.55419
C	7.44388	0.09446	-1.11648
C	6.88689	-1.18292	-1.26452
C	5.58260	-1.41614	-0.86371
C	-0.00197	2.61089	1.99644
C	-0.00240	4.00133	1.91966
C	-0.00093	4.63198	0.67762
C	0.00091	3.87287	-0.49118
C	0.00125	2.48252	-0.42009
C	-5.58269	-1.41657	-0.86271
C	-6.88694	-1.18340	-1.26367
C	-7.44363	0.09425	-1.11680
C	-6.67892	1.12699	-0.55553
C	-5.38022	0.88026	-0.14663
C	8.78641	0.34333	-1.53462
C	-8.78612	0.34306	-1.53509
N	9.87927	0.54628	-1.87539
N	-9.87897	0.54577	-1.87604

H	-2.54753	1.35163	0.08477
H	2.54787	1.35169	0.08476
H	4.81052	1.67947	0.31588
H	7.11232	2.11373	-0.42972
H	7.47476	-1.98236	-1.70244
H	5.15647	-2.40420	-1.01133
H	-0.00304	2.10205	2.95538
H	-0.00383	4.59134	2.83109
H	-0.00122	5.71628	0.61957
H	0.00201	4.36276	-1.46014
H	0.00255	1.88009	-1.32402
H	-5.15683	-2.40488	-1.00943
H	-7.47502	-1.98313	-1.70079
H	-7.11151	2.11408	-0.43198
H	-4.80982	1.67999	0.31387

SCF Done: E(RPBE1PBE) = -2432.94087638 A.U. after 1 cycles

Zero-point correction= 0.353628 (Hartree/Particle)

Thermal correction to Energy= 0.381593

Thermal correction to Enthalpy= 0.382537

Thermal correction to Gibbs Free Energy= 0.290290

Sum of electronic and zero-point Energies= -2432.587249

Sum of electronic and thermal Energies= -2432.559284

Sum of electronic and thermal Enthalpies= -2432.558339

Sum of electronic and thermal Free Energies= -2432.650586

6.1.5.2 Computed Excitations of compound 3d-ss (PBE1PBE/6-31G PCM CH_2Cl_2)**

Excited State 1: Singlet-A 2.5717 eV 482.11 nm f=0.3337 <S**2>=0.000

126 ->127 0.69512

This state for optimization and/or second-order correction.

Total Energy, E(TD-HF/TD-KS) = -2432.84636815

Copying the excited state density for this state as the 1-particle RhoCI density.

Excited State 2: Singlet-A 2.7733 eV 447.07 nm f=0.0641 <S**2>=0.000

126 ->128 0.70188

Excited State 3: Singlet-A 3.7344 eV 332.00 nm f=0.1958 <S**2>=0.000

126 ->127 0.11095

126 ->129 0.59025

126 ->131 0.27203

126 ->133 -0.15229

126 ->135 0.13626

Excited State 4: Singlet-A 3.8794 eV 319.60 nm f=0.0203 <S**2>=0.000

126 ->131 -0.18579

126 ->132 0.64948

126 ->133 -0.14319

Excited State 5: Singlet-A 3.9905 eV 310.70 nm f=1.2624 <S**2>=0.000

124 ->127 -0.22093

125 ->128 0.65191

Excited State 6: Singlet-A 4.0077 eV 309.37 nm f=0.0056 <S**2>=0.000

125 ->127 -0.14391

126 ->130 0.66585

126 ->136 0.13452

6.1.5.3 Computed xyz-Coordinates of radical cation of compound 3d-ss (uB3LYP/6-311G*)

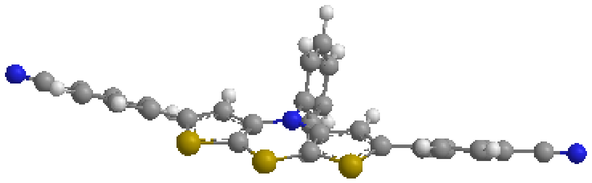

Figure S51. Optimized ground state geometry of radical cation of **3d-ss** (uB3LYP/6-311G* PCM CH_2Cl_2).

C	-1.30952	-1.75996	-0.09237
C	-1.20915	-0.36726	-0.01911
N	0.00000	0.30769	0.02314
C	1.20915	-0.36727	-0.01902
C	1.30953	-1.75997	-0.09223
S	0.00000	-2.89556	-0.14354
C	2.48568	0.25723	0.01862
C	3.53076	-0.62967	-0.02801
S	2.95922	-2.29013	-0.13547
S	-2.95921	-2.29012	-0.13566
C	-3.53076	-0.62968	-0.02810
C	-2.48568	0.25723	0.01860
C	-4.96475	-0.34363	-0.01464
C	-0.00000	1.75902	0.09383
C	4.96475	-0.34363	-0.01460
C	-5.44154	0.87340	-0.53238
C	-6.79492	1.16741	-0.51558
C	-7.70813	0.24498	0.01702
C	-7.24349	-0.97424	0.53029
C	-5.88787	-1.26168	0.51374
C	-0.00050	2.49750	-1.08745
C	-0.00050	3.88904	-1.01433
C	-0.00001	4.52453	0.22675
C	0.00049	3.77289	1.40094
C	0.00050	2.38062	1.34048
C	5.88784	-1.26149	0.51413
C	7.24347	-0.97405	0.53061
C	7.70812	0.24498	0.01691
C	6.79493	1.16723	-0.51603
C	5.44155	0.87322	-0.53276

C	-9.10527	0.54673	0.03354
C	9.10527	0.54672	0.03335
N	-10.23329	0.79257	0.04722
N	10.23329	0.79256	0.04697
H	2.61920	1.32492	0.11278
H	-2.61919	1.32491	0.11285
H	-4.75244	1.58168	-0.97766
H	-7.15491	2.10437	-0.92321
H	-7.94672	-1.68545	0.94631
H	-5.54612	-2.19748	0.94304
H	-0.00085	1.99130	-2.04669
H	-0.00088	4.47429	-1.92694
H	-0.00001	5.60749	0.27903
H	0.00088	4.26767	2.36552
H	0.00088	1.78511	2.24694
H	5.54607	-2.19713	0.94375
H	7.94669	-1.68511	0.94692
H	7.15495	2.10404	-0.92399
H	4.75246	1.58134	-0.97830

SCF Done: E(UB3LYP) = -2434.89020227   A.U. after   1 cycles

Zero-point correction= 0.349606 (Hartree/Particle)

Thermal correction to Energy= 0.377689

Thermal correction to Enthalpy= 0.378633

Thermal correction to Gibbs Free Energy= 0.286154

Sum of electronic and zero-point Energies= -2434.540596

Sum of electronic and thermal Energies= -2434.512513

Sum of electronic and thermal Enthalpies= -2434.511569

Sum of electronic and thermal Free Energies= -2434.604048

6.1.5.4 Computed xyz-Coordinates of dication (S_0) of compound 3d-ss (uB3LYP/6-311G*)

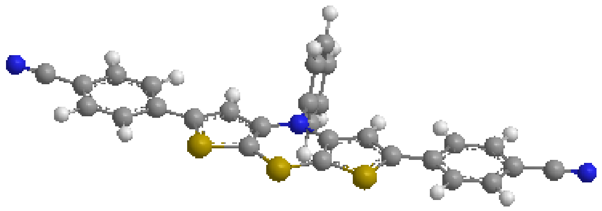

Figure S52. Optimized ground state geometry of dication of **3d-ss** (uB3LYP/6-311G* PCM CH_2Cl_2).

C	-1.31137	-1.74973	-0.00017
C	-1.21089	-0.32763	0.00011
N	0.00000	0.32534	0.00001
C	1.21089	-0.32763	-0.00034
C	1.31137	-1.74973	-0.00054
S	0.00000	-2.82924	-0.00050
C	2.47445	0.28473	-0.00017
C	3.53008	-0.61263	-0.00039
S	2.94917	-2.29002	-0.00110
S	-2.94917	-2.29002	0.00004
C	-3.53007	-0.61263	0.00015
C	-2.47445	0.28473	0.00022
C	-4.94758	-0.34477	0.00024
C	0.00000	1.78961	0.00017
C	4.94758	-0.34477	-0.00029
C	-5.41952	0.99022	0.00462
C	-6.77199	1.26075	0.00463
C	-7.70222	0.20352	0.00024
C	-7.25052	-1.12882	-0.00409
C	-5.89624	-1.39436	-0.00405
C	-0.00001	2.46219	-1.21982
C	-0.00003	3.85614	-1.21003
C	-0.00000	4.54921	0.00043
C	0.00002	3.85591	1.21076

C	0.00001	2.46195	1.22028
C	5.89624	-1.39435	0.00676
C	7.25051	-1.12881	0.00708
C	7.70222	0.20352	0.00022
C	6.77199	1.26074	-0.00701
C	5.41952	0.99021	-0.00723
C	-9.09866	0.48420	0.00022
C	9.09866	0.48420	0.00050
N	-10.23101	0.71424	0.00020
N	10.23100	0.71424	0.00075
H	2.60265	1.35613	0.00087
H	-2.60265	1.35613	-0.00009
H	-4.72626	1.82193	0.00843
H	-7.12534	2.28455	0.00812
H	-7.96801	-1.94001	-0.00751
H	-5.57982	-2.43242	-0.00782
H	-0.00005	1.91428	-2.15600
H	-0.00007	4.39730	-2.14919
H	-0.00001	5.63319	0.00053
H	0.00006	4.39689	2.15002
H	0.00005	1.91386	2.15636
H	5.57981	-2.43240	0.01279
H	7.96801	-1.93998	0.01272
H	7.12534	2.28453	-0.01257
H	4.72627	1.82190	-0.01352

SCF Done: E(UB3LYP) = -2434.52512875   A.U. after   1 cycles

Zero-point correction= 0.349435 (Hartree/Particle)

Thermal correction to Energy= 0.377614

Thermal correction to Enthalpy= 0.378558

Thermal correction to Gibbs Free Energy= 0.285139

Sum of electronic and zero-point Energies= -2434.175693

Sum of electronic and thermal Energies= -2434.147515

Sum of electronic and thermal Enthalpies= -2434.146571

Sum of electronic and thermal Free Energies= -2434.239989

6.1.5.5 Reoptimization of compound 3d-ss (uB3LYP/6-311G*)

Compound **3d-ss** in the gas phase (uB3LYP/6-311G*):

SCF Done: E(UB3LYP) = -2435.11888743 A.U. after 1 cycles
Zero-point correction= 0.348946 (Hartree/Particle)
Thermal correction to Energy= 0.377110
Thermal correction to Enthalpy= 0.378054
Thermal correction to Gibbs Free Energy= 0.285795
Sum of electronic and zero-point Energies= -2434.769941
Sum of electronic and thermal Energies= -2434.741777
Sum of electronic and thermal Enthalpies= -2434.740833
Sum of electronic and thermal Free Energies= -2434.833093

Compound **3d-ss** in CH_2Cl_2 (uB3LYP/6-311G* SMD CH_2Cl_2):

SCF Done: E(UB3LYP) = -2435.16302718 A.U. after 14 cycles

Radical cation of compound **3d-ss** in CH_2Cl_2 (uB3LYP/6-311G* SMD CH_2Cl_2):

SCF Done: E(UB3LYP) = -2434.99014177 A.U. after 20 cycles

Dication of compound **3d-ss** in CH_2Cl_2 (uB3LYP/6-311G* SMD CH_2Cl_2):

SCF Done: E(UB3LYP) = -2434.76722517 A.U. after 15 cycles

6.1.5.6 Computed xyz-Coordinates of transition state of the benzonitrile rotation of compound 3d-ss (PBE1PBE/6-31G PCM CH_2Cl_2)**

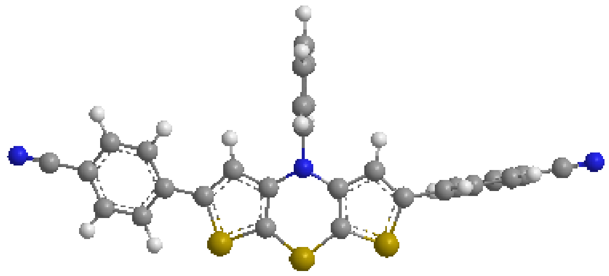

Figure S53. Optimized geometry of the transition state of the benzonitrile rotation of **3d-ss** (PBE1PBE/6-31G** PCM CH_2Cl_2).

C	-1.29614	-1.63782	-0.88352
C	-1.19911	-0.27628	-0.73027
N	-0.00001	0.42150	-0.94570
C	1.19650	-0.27828	-0.73440
C	1.28722	-1.64489	-0.88178
S	-0.00458	-2.66163	-1.51118
C	2.42302	0.29492	-0.30415
C	3.42430	-0.63021	-0.12342
S	2.85028	-2.23745	-0.45785
S	-2.86049	-2.22507	-0.44392
C	-3.41770	-0.61805	-0.10192
C	-2.42462	0.30406	-0.28433
C	0.00344	1.85002	-0.86379
C	-4.81121	-0.38066	0.33047
C	4.80110	-0.39089	0.29548
C	-5.81346	-0.14240	-0.61730
C	-7.12153	0.09188	-0.21854
C	-7.43896	0.08601	1.14566
C	-6.44498	-0.15361	2.10283
C	-5.14060	-0.38638	1.69082
C	0.01292	2.59465	-2.04151
C	0.01740	3.98591	-1.98040

C	0.01240	4.63049	-0.74560
C	0.00318	3.88431	0.43145
C	-0.00103	2.49329	0.37572
C	5.57993	-1.41059	0.86723
C	6.88458	-1.17523	1.26546
C	7.44180	0.10117	1.10957
C	6.67673	1.13037	0.54217
C	5.37763	0.88137	0.13616
C	-8.78578	0.32637	1.56392
C	8.78478	0.35222	1.52464
N	-9.87952	0.52183	1.90397
N	9.87814	0.55684	1.86294
H	2.54818	1.35332	-0.11253
H	-2.56357	1.36379	-0.10932
H	-5.56092	-0.13893	-1.67270
H	-7.89607	0.27886	-0.95446
H	-6.69776	-0.15635	3.15762
H	-4.36573	-0.57280	2.42738
H	0.01638	2.07517	-2.99474
H	0.02445	4.56563	-2.89840
H	0.01557	5.71537	-0.69961
H	-0.00075	4.38500	1.39490
H	-0.00803	1.90104	1.28632
H	5.15415	-2.39793	1.02060
H	7.47279	-1.97231	1.70724
H	7.10950	2.11649	0.41158
H	4.80725	1.67838	-0.32904

SCF Done: E(RPBE1PBE) = -2432.93445701 A.U. after 1 cycles

Zero-point correction= 0.353478 (Hartree/Particle)

Thermal correction to Energy= 0.380647

Thermal correction to Enthalpy= 0.381591

Thermal correction to Gibbs Free Energy= 0.291626

Sum of electronic and zero-point Energies= -2432.580979

Sum of electronic and thermal Energies= -2432.553810

Sum of electronic and thermal Enthalpies= -2432.552866

Sum of electronic and thermal Free Energies= -2432.642831

6.1.6 Compound 3e-aa

6.1.6.1 Computed xyz-Coordinates of compound 3e-aa (PBE1PBE/6-31G** PCM CH_2Cl_2)

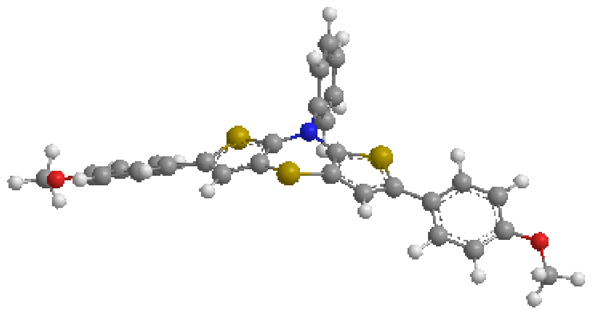

Figure S54. Optimized ground state geometry of **3e-aa** (PBE1PBE/6-31G** PCM CH_2Cl_2).

C	1.36168	-0.96108	1.06860
C	1.18283	0.33131	0.64697
N	-0.01720	1.03656	0.69871
C	-1.18166	0.27411	0.69057
C	-1.28430	-1.02479	1.11736
S	0.07394	-1.85459	1.90286
S	-2.67100	0.82775	-0.01279
C	-3.45053	-0.70753	0.30243
C	-2.56861	-1.58371	0.88269
C	2.67526	-1.44437	0.82762
C	3.50041	-0.51867	0.24120
S	2.62703	0.96299	-0.08091
C	-0.06332	2.43437	0.39742
C	-4.86181	-0.90817	-0.01700
C	4.91457	-0.64274	-0.10605

C	5.76332	0.47534	-0.17852
C	7.10211	0.34115	-0.50101
C	7.64565	-0.92393	-0.75445
C	6.81909	-2.04883	-0.68767
C	5.47181	-1.89868	-0.37621
C	-5.62288	-1.86040	0.68547
C	-6.95394	-2.07909	0.38229
C	-7.58265	-1.33801	-0.62690
C	-6.84890	-0.37849	-1.32796
C	-5.50519	-0.18020	-1.02404
O	8.96539	-0.95402	-1.05611
O	-8.88887	-1.62019	-0.84361
C	-9.56355	-0.88873	-1.84904
C	9.55557	-2.21385	-1.31193
C	-0.15174	3.35517	1.44046
C	-0.20077	4.71536	1.15132
C	-0.15958	5.15052	-0.17214
C	-0.07229	4.22714	-1.21108
C	-0.02641	2.86394	-0.93045
H	-2.82760	-2.60468	1.13937
H	3.01023	-2.42995	1.13065
H	5.37246	1.46582	0.03983
H	7.75348	1.20795	-0.55129
H	7.20689	-3.04041	-0.89079
H	4.83833	-2.78068	-0.36042
H	-5.16554	-2.42376	1.49332
H	-7.53719	-2.81342	0.92901
H	-7.30449	0.20830	-2.11728
H	-4.94581	0.54978	-1.60382
H	-10.58799	-1.26140	-1.85853
H	-9.57192	0.18505	-1.62757

H	-9.11163	-1.05028	-2.83483
H	10.60764	-2.01826	-1.52032
H	9.47719	-2.87838	-0.44336
H	9.10054	-2.70223	-2.18181
H	-0.17952	2.99602	2.46438
H	-0.26897	5.43580	1.96057
H	-0.19624	6.21285	-0.39381
H	-0.04241	4.56577	-2.24205
H	0.03622	2.13257	-1.73078

SCF Done: E(RPBE1PBE) = -2477.46172030 A.U. after 1 cycles

Zero-point correction= 0.421820 (Hartree/Particle)

Thermal correction to Energy= 0.451420

Thermal correction to Enthalpy= 0.452364

Thermal correction to Gibbs Free Energy= 0.356801

Sum of electronic and zero-point Energies= -2477.039900

Sum of electronic and thermal Energies= -2477.010300

Sum of electronic and thermal Enthalpies= -2477.009356

Sum of electronic and thermal Free Energies= -2477.104920

6.1.6.2 Computed Excitations of compound 3e-aa (PBE1PBE/6-31+G PCM CH_2Cl_2)**

Excited State 1: Singlet-A 2.8418 eV 436.28 nm f=0.5106 <S**2>=0.000

130 ->131 0.69361

This state for optimization and/or second-order correction.

Total Energy, E(TD-HF/TD-KS) = -2477.39224026

Copying the excited state density for this state as the 1-particle RhoCI density.

Excited State 2: Singlet-A 3.3008 eV 375.62 nm f=0.0177 <S**2>=0.000

130 ->132 0.55144

130 ->133 -0.43242

Excited State 3: Singlet-A 3.4207 eV 362.46 nm f=0.0216 <S**2>=0.000

130 ->132 0.41868

130 ->133 0.54327

130 ->134 0.12471

Excited State 4: Singlet-A 3.5330 eV 350.93 nm f=0.0050 <S**2>=0.000
130 ->134 0.68877

Excited State 5: Singlet-A 3.7781 eV 328.17 nm f=0.0056 <S**2>=0.000
130 ->135 0.65676
130 ->137 0.11586
130 ->139 0.16077

Excited State 6: Singlet-A 3.8321 eV 323.54 nm f=0.0764 <S**2>=0.000
130 ->136 0.58030
130 ->137 -0.32587
130 ->139 0.10734

Excited State 7: Singlet-A 3.9458 eV 314.22 nm f=0.1393 <S**2>=0.000
130 ->136 0.36415
130 ->137 0.55761

Excited State 8: Singlet-A 4.1287 eV 300.30 nm f=0.0694 <S**2>=0.000
129 ->131 0.68317

Excited State 9: Singlet-A 4.2000 eV 295.20 nm f=0.0038 <S**2>=0.000
129 ->131 0.10723
130 ->135 -0.16942
130 ->138 -0.15416
130 ->139 0.61556

Excited State 10: Singlet-A 4.2552 eV 291.37 nm f=0.0096 <S**2>=0.000
130 ->137 0.11228
130 ->138 0.64203
130 ->139 0.15602
130 ->143 -0.13177

6.1.6.3 Computed xyz-Coordinates of radical cation of compound 3e-aa (uB3LYP/6-311G*)

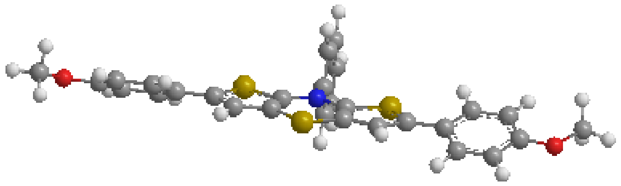

Figure S55. Optimized ground state geometry of radical cation of **3e-aa** (uB3LYP/6-311G* PCM CH_2Cl_2).

C	-1.37982	-1.28942	-0.01034
C	-1.19173	0.08945	-0.00869
N	0.01832	0.74429	-0.01373
C	1.19057	0.02441	-0.02944
C	1.30240	-1.36261	-0.04378
S	-0.06964	-2.46074	-0.02996
S	2.75495	0.79269	-0.03788
C	3.56517	-0.77619	-0.03380
C	2.64363	-1.80182	-0.04386
C	-2.74318	-1.65401	-0.02248
C	-3.60655	-0.57881	-0.02501
S	-2.71095	0.94266	0.00008
C	0.05735	2.19678	-0.00543
C	5.01507	-0.85825	-0.03862
C	-5.05890	-0.57551	-0.03057
C	-5.79841	0.53093	-0.50111
C	-7.17667	0.51781	-0.51002
C	-7.88045	-0.60779	-0.04705
C	-7.16364	-1.71671	0.42740
C	-5.77698	-1.69128	0.43338
C	5.66289	-2.02109	-0.51231
C	7.03689	-2.12115	-0.51253
C	7.82984	-1.05928	-0.04061
C	7.20567	0.10472	0.43016

C	5.82087	0.19342	0.42728
O	-9.22153	-0.52091	-0.09798
O	9.15924	-1.25768	-0.08520
C	10.03840	-0.22733	0.36664
C	-10.01414	-1.62233	0.34684
C	0.10882	2.88174	-1.21800
C	0.14474	4.27367	-1.20275
C	0.12843	4.96052	0.01028
C	0.07662	4.26164	1.21542
C	0.04076	2.86963	1.21488
H	2.92403	-2.84654	-0.01625
H	-3.08064	-2.68124	-0.06607
H	-5.28521	1.40369	-0.89222
H	-7.74452	1.36291	-0.88071
H	-7.67667	-2.59244	0.80288
H	-5.24615	-2.54711	0.83545
H	5.07901	-2.84288	-0.91144
H	7.53541	-3.00817	-0.88525
H	7.78679	0.93581	0.80753
H	5.36401	1.09434	0.82476
H	11.04234	-0.61918	0.22524
H	9.91515	0.68348	-0.22594
H	9.87781	-0.00928	1.42619
H	-11.04646	-1.31515	0.20060
H	-9.81227	-2.51827	-0.24684
H	-9.84170	-1.82896	1.40680
H	0.11904	2.33347	-2.15345
H	0.18449	4.81932	-2.13861
H	0.15569	6.04447	0.01645
H	0.06381	4.79800	2.15739
H	0.00050	2.31210	2.14403

SCF Done: E(UB3LYP) = -2479.48804651 A.U. after 1 cycles

Zero-point correction= 0.417346 (Hartree/Particle)

Thermal correction to Energy= 0.447134

Thermal correction to Enthalpy= 0.448079

Thermal correction to Gibbs Free Energy= 0.352290

Sum of electronic and zero-point Energies= -2479.070701

Sum of electronic and thermal Energies= -2479.040912

Sum of electronic and thermal Enthalpies= -2479.039968

Sum of electronic and thermal Free Energies= -2479.135757

6.1.6.4 Computed xyz-Coordinates of dication (S_0) of compound 3e-aa (uB3LYP/6-311G*)

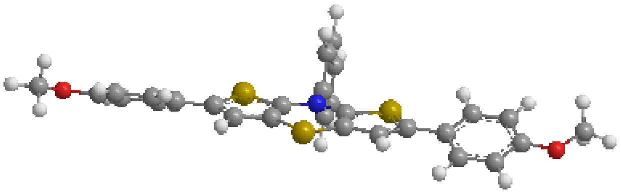

Figure S56. Optimized ground state geometry of dication of **3e-aa** (uB3LYP/6-311G* PCM CH_2Cl_2).

C	-1.37982	-1.28942	-0.01034
C	-1.19173	0.08945	-0.00869
N	0.01832	0.74429	-0.01373
C	1.19057	0.02441	-0.02944
C	1.30240	-1.36261	-0.04378
S	-0.06964	-2.46074	-0.02996
S	2.75495	0.79269	-0.03788
C	3.56517	-0.77619	-0.03380
C	2.64363	-1.80182	-0.04386
C	-2.74318	-1.65401	-0.02248
C	-3.60655	-0.57881	-0.02501
S	-2.71095	0.94266	0.00008
C	0.05735	2.19678	-0.00543

C	5.01507	-0.85825	-0.03862
C	-5.05890	-0.57551	-0.03057
C	-5.79841	0.53093	-0.50111
C	-7.17667	0.51781	-0.51002
C	-7.88045	-0.60779	-0.04705
C	-7.16364	-1.71671	0.42740
C	-5.77698	-1.69128	0.43338
C	5.66289	-2.02109	-0.51231
C	7.03689	-2.12115	-0.51253
C	7.82984	-1.05928	-0.04061
C	7.20567	0.10472	0.43016
C	5.82087	0.19342	0.42728
O	-9.22153	-0.52091	-0.09798
O	9.15924	-1.25768	-0.08520
C	10.03840	-0.22733	0.36664
C	-10.01414	-1.62233	0.34684
C	0.10882	2.88174	-1.21800
C	0.14474	4.27367	-1.20275
C	0.12843	4.96052	0.01028
C	0.07662	4.26164	1.21542
C	0.04076	2.86963	1.21488
H	2.92403	-2.84654	-0.01625
H	-3.08064	-2.68124	-0.06607
H	-5.28521	1.40369	-0.89222
H	-7.74452	1.36291	-0.88071
H	-7.67667	-2.59244	0.80288
H	-5.24615	-2.54711	0.83545
H	5.07901	-2.84288	-0.91144
H	7.53541	-3.00817	-0.88525
H	7.78679	0.93581	0.80753
H	5.36401	1.09434	0.82476

H	11.04234	-0.61918	0.22524
H	9.91515	0.68348	-0.22594
H	9.87781	-0.00928	1.42619
H	-11.04646	-1.31515	0.20060
H	-9.81227	-2.51827	-0.24684
H	-9.84170	-1.82896	1.40680
H	0.11904	2.33347	-2.15345
H	0.18449	4.81932	-2.13861
H	0.15569	6.04447	0.01645
H	0.06381	4.79800	2.15739
H	0.00050	2.31210	2.14403

SCF Done: E(UB3LYP) = -2479.48804651 A.U. after 1 cycles

Zero-point correction= 0.417346 (Hartree/Particle)

Thermal correction to Energy= 0.447134

Thermal correction to Enthalpy= 0.448079

Thermal correction to Gibbs Free Energy= 0.352290

Sum of electronic and zero-point Energies= -2479.070701

Sum of electronic and thermal Energies= -2479.040912

Sum of electronic and thermal Enthalpies= -2479.039968

Sum of electronic and thermal Free Energies= -2479.135757

6.1.6.5 Reoptimization of compound 3e-aa (uB3LYP/6-311G*)

Compound **3e-aa** in the gas phase (uB3LYP/6-311G*):

SCF Done: E(UB3LYP) = -2479.68784172 A.U. after 1 cycles

Zero-point correction= 0.416387 (Hartree/Particle)

Thermal correction to Energy= 0.446206

Thermal correction to Enthalpy= 0.447150

Thermal correction to Gibbs Free Energy= 0.350665

Sum of electronic and zero-point Energies= -2479.271455

Sum of electronic and thermal Energies= -2479.241636

Sum of electronic and thermal Enthalpies= -2479.240691

Sum of electronic and thermal Free Energies= -2479.337177

Compound **3e-aa** in CH_2Cl_2 (uB3LYP/6-311G* SMD CH_2Cl_2):

SCF Done: E(UB3LYP) = -2479.72752041 A.U. after 13 cycles

Radical cation of compound **3e-aa** in CH_2Cl_2 (uB3LYP/6-311G* SMD CH_2Cl_2):

SCF Done: E(UB3LYP) = -2479.56877858 A.U. after 19 cycles

Dication of compound **3e-aa** in CH_2Cl_2 (uB3LYP/6-311G* SMD CH_2Cl_2):

SCF Done: E(UB3LYP) = -2479.37252684 A.U. after 15 cycles

6.1.6.6 Computed xyz-Coordinates of transition state of the anisyl rotation of compound 3e-aa (PBE1PBE/6-31G** PCM CH_2Cl_2)

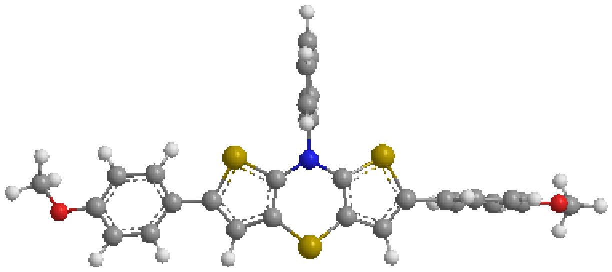

Figure S**57**. Optimized geometry of the transition state of the anisyl rotation of **3e-aa** (PBE1PBE/6-31G** PCM CH_2Cl_2).

C	-1.34083	-0.91499	-1.22429
C	-1.18479	0.34707	-0.71399
N	0.00860	1.06881	-0.71219
C	1.18108	0.31917	-0.72416
C	1.30615	-0.94783	-1.23289
S	-0.02949	-1.72826	-2.10328
S	2.64775	0.83505	0.05286
C	3.44826	-0.67070	-0.34344
C	2.58886	-1.51265	-1.00258
C	-2.64497	-1.44404	-1.00543
C	-3.47874	-0.58101	-0.35233
S	-2.64048	0.89767	0.05721
C	0.03260	2.45038	-0.34266

C	4.85276	-0.88173	-0.00112
C	-4.90448	-0.74750	-0.00660
C	-5.91375	-0.34663	-0.89492
C	-7.25110	-0.51297	-0.57683
C	-7.61756	-1.08566	0.64787
C	-6.62600	-1.48810	1.54620
C	-5.28509	-1.31290	1.21209
C	5.64266	-1.77485	-0.74786
C	6.96716	-2.00331	-0.42353
C	7.55990	-1.33117	0.65348
C	6.79723	-0.43026	1.39992
C	5.46046	-0.22209	1.07294
O	-8.94730	-1.20622	0.86840
O	8.86239	-1.61769	0.88683
C	9.50102	-0.95643	1.96197
C	-9.36543	-1.77746	2.09360
C	0.11278	3.42196	-1.33937
C	0.14064	4.76725	-0.98543
C	0.08704	5.13769	0.35717
C	0.00762	4.16420	1.34995
C	-0.01789	2.81550	1.00405
H	2.86336	-2.51226	-1.32004
H	-2.96193	-2.42295	-1.34724
H	-5.63940	0.09724	-1.84758
H	-8.03403	-0.20680	-1.26350
H	-6.88268	-1.93423	2.50004
H	-4.51833	-1.62747	1.91433
H	5.21331	-2.28299	-1.60614
H	7.57296	-2.69160	-1.00457
H	7.22474	0.10254	2.24145
H	4.87725	0.45953	1.68708

H	10.52792	-1.32220	1.97502
H	9.50683	0.13061	1.81898
H	9.02200	-1.19165	2.91975
H	-10.45527	-1.78213	2.06838
H	-9.00237	-2.80622	2.20179
H	-9.02650	-1.18304	2.95020
H	0.15109	3.11270	-2.37913
H	0.20246	5.52664	-1.75880
H	0.10756	6.18850	0.63002
H	-0.03247	4.45251	2.39581
H	-0.07519	2.04602	1.76820

SCF Done: E(RPBE1PBE) = -2477.45693660 A.U. after 1 cycles

Zero-point correction= 0.421725 (Hartree/Particle)

Thermal correction to Energy= 0.450468

Thermal correction to Enthalpy= 0.451413

Thermal correction to Gibbs Free Energy= 0.358457

Sum of electronic and zero-point Energies= -2477.035211

Sum of electronic and thermal Energies= -2477.006468

Sum of electronic and thermal Enthalpies= -2477.005524

Sum of electronic and thermal Free Energies= -2477.098479

6.1.7 Compound 3f-ss

6.1.7.1 Computed xyz-Coordinates of compound 3f-ss (PBE1PBE/6-31G** PCM CH_2Cl_2)

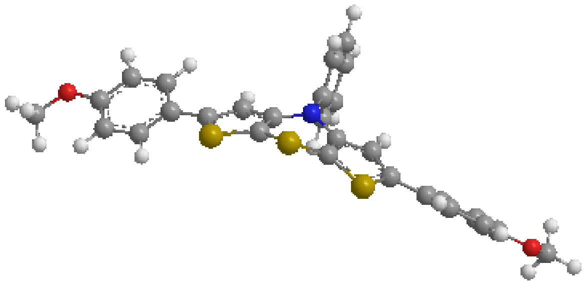

Figure S58. Optimized ground state geometry of **3f-ss** (PBE1PBE/6-31G** PCM CH_2Cl_2).

C	-1.20410	-0.38322	-0.76938
C	-1.25983	-1.75588	-0.81871
S	0.05397	-2.78680	-1.38140
C	1.32210	-1.68712	-0.84649
C	1.19243	-0.31973	-0.79330
N	-0.02668	0.33184	-1.04379
C	-2.44969	0.19283	-0.38895
C	-3.43051	-0.73685	-0.14486
S	-2.81191	-2.35127	-0.34598
S	2.90875	-2.19836	-0.38833
C	3.43045	-0.55440	-0.15456
C	2.40125	0.32133	-0.39844
C	-4.81667	-0.50556	0.25798
C	-0.06173	1.76180	-1.02381
C	4.80450	-0.24703	0.23905
C	-5.83821	-1.42193	-0.04530
C	-7.14721	-1.18856	0.33562
C	-7.48440	-0.01931	1.02892
C	-6.48341	0.90535	1.33929
C	-5.16905	0.65236	0.96140
C	-0.10264	2.45809	0.18624

C	-0.13377	3.84979	0.18246
C	-0.12257	4.54547	-1.02525
C	-0.08070	3.84885	-2.23060
C	-0.05057	2.45633	-2.23165
C	5.38431	0.98604	-0.10867
C	6.67798	1.29900	0.26545
C	7.44720	0.38080	0.99239
C	6.89254	-0.85325	1.34025
C	5.58509	-1.15044	0.96813
O	-8.79024	0.12145	1.35362
C	-9.17762	1.29296	2.04628
O	8.70237	0.77714	1.30543
C	9.51803	-0.12209	2.03235
H	-2.61990	1.26075	-0.33016
H	2.49400	1.39316	-0.27424
H	-5.60614	-2.32346	-0.60638
H	-7.93358	-1.89681	0.09485
H	-6.71081	1.81360	1.88525
H	-4.39925	1.36666	1.23816
H	-0.10870	1.90477	1.12100
H	-0.16536	4.39073	1.12348
H	-0.14631	5.63113	-1.02601
H	-0.07209	4.38872	-3.17264
H	-0.01880	1.89707	-3.16159
H	4.81633	1.69972	-0.69793
H	7.12369	2.25010	-0.00828
H	7.45972	-1.58196	1.90757
H	5.16272	-2.10471	1.27269
H	-10.25331	1.20972	2.20231
H	-8.67689	1.36909	3.01861
H	-8.96662	2.19485	1.45968

H 10.47430 0.38241 2.17155

H 9.08555 -0.35296 3.01292

H 9.67896 -1.05508 1.47965

SCF Done: E(RPBE1PBE) = -2477.46208170 A.U. after 1 cycles

Zero-point correction= 0.422024 (Hartree/Particle)

Thermal correction to Energy= 0.451555

Thermal correction to Enthalpy= 0.452499

Thermal correction to Gibbs Free Energy= 0.357169

Sum of electronic and zero-point Energies= -2477.040058

Sum of electronic and thermal Energies= -2477.010527

Sum of electronic and thermal Enthalpies= -2477.009583

Sum of electronic and thermal Free Energies= -2477.104913

6.1.7.2 Computed Excitations of compound 3f-ss (PBE1PBE/6-31+G PCM CH_2Cl_2)**

Excited State 1: Singlet-A 2.8611 eV 433.34 nm f=0.1836 <S**2>=0.000

130 ->131 0.69387

This state for optimization and/or second-order correction.

Total Energy, E(TD-HF/TD-KS) = -2477.39220185

Copying the excited state density for this state as the 1-particle RhoCI density.

Excited State 2: Singlet-A 3.2453 eV 382.05 nm f=0.0793 <S**2>=0.000

130 ->132 0.69921

Excited State 3: Singlet-A 3.5422 eV 350.02 nm f=0.0036 <S**2>=0.000

130 ->133 0.69737

Excited State 4: Singlet-A 3.7847 eV 327.59 nm f=0.0095 <S**2>=0.000

130 ->134 0.37801

130 ->135 -0.34325

130 ->136 0.42339

130 ->137 0.18403

Excited State 5: Singlet-A 3.8607 eV 321.14 nm f=0.0093 <S**2>=0.000

130 ->134 0.55039

130 ->135 0.25241

130 ->136 -0.24563

130 ->137 -0.18212

130 ->139 0.11002

Excited State 6: Singlet-A 3.8972 eV 318.14 nm f=0.0772 <S**2>=0.000

130 ->135 -0.18166

130 ->136 -0.43305

130 ->137 0.46011

130 ->138 -0.16726

Excited State 7: Singlet-A 3.9486 eV 314.00 nm f=0.0579 <S**2>=0.000

130 ->135 0.51009

130 ->136 0.22482

130 ->137 0.38476

Excited State 8: Singlet-A 4.0929 eV 302.92 nm f=0.0962 <S**2>=0.000

128 ->132 0.10276

129 ->131 0.68392

Excited State 9: Singlet-A 4.1765 eV 296.86 nm f=1.2881 <S**2>=0.000

128 ->131 0.31609

129 ->132 0.60457

130 ->139 -0.10562

Excited State 10: Singlet-A 4.2531 eV 291.51 nm f=0.0337 <S**2>=0.000

128 ->131 -0.12212

130 ->134 0.14769

130 ->137	0.16117
130 ->138	0.47059
130 ->139	-0.41738

6.1.7.3 Computed xyz-Coordinates of T_1 of compound 3f-ss (uPBE1PBE/6-31G** PCM toluene)

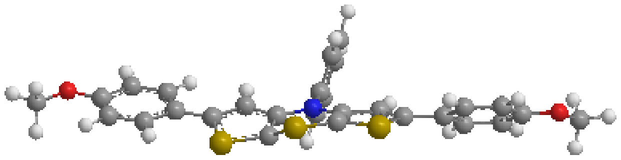

Figure S59. Optimized T1 geometry of **3f-ss** (uB3LYP/6-311G* PCM CH_2Cl_2).

C	1.22689	-1.75279	0.17343
C	1.19922	-0.37591	0.00807
N	0.02895	0.36561	-0.07377
C	-1.20929	-0.23669	-0.18356
C	-1.30103	-1.67363	-0.45796
S	-0.16187	-2.78028	0.27063
C	-2.43669	0.37650	-0.04925
C	-3.53997	-0.48342	-0.20640
S	-2.98454	-2.14207	-0.60729
S	2.85170	-2.35594	0.22012
C	3.50765	-0.74577	0.06628
C	2.50952	0.18332	-0.05293
C	4.95243	-0.52982	0.05496
C	0.09454	1.78986	0.03997
C	-4.93058	-0.19477	-0.08907
C	5.50329	0.56574	-0.61945
C	6.87327	0.80241	-0.62797
C	7.73290	-0.07611	0.03731
C	7.19903	-1.18383	0.70678
C	5.83383	-1.40168	0.71653

C	0.46301	2.37302	1.25103
C	0.52652	3.75990	1.35183
C	0.21288	4.55655	0.25328
C	-0.16165	3.96619	-0.95223
C	-0.21963	2.58061	-1.06418
C	-5.91412	-1.20336	-0.19765
C	-7.27097	-0.92494	-0.09694
C	-7.70014	0.38690	0.11674
C	-6.74327	1.40946	0.22833
C	-5.39891	1.12850	0.12987
O	9.07812	0.05471	0.08904
O	-8.99630	0.77308	0.22924
C	9.66024	1.16237	-0.56658
C	-9.98796	-0.22475	0.12465
H	-2.54784	1.43315	0.15850
H	2.69822	1.24524	-0.14715
H	4.85183	1.23915	-1.16854
H	7.25853	1.66085	-1.16608
H	7.87733	-1.85536	1.22304
H	5.43969	-2.25297	1.26544
H	0.69575	1.73896	2.10100
H	0.81387	4.21709	2.29374
H	0.25964	5.63818	0.33623
H	-0.40309	4.58490	-1.81115
H	-0.50514	2.10424	-1.99690
H	-5.61037	-2.23488	-0.35544
H	-7.98324	-1.73756	-0.18567
H	-7.08985	2.42490	0.39437
H	-4.68810	1.94392	0.21901
H	10.73420	1.08472	-0.39556
H	9.29678	2.11116	-0.15343

H	9.46482	1.14021	-1.64559
H	-10.94550	0.28424	0.24091
H	-9.88488	-0.98159	0.91245
H	-9.96064	-0.72082	-0.85373

SCF Done: E(UPBE1PBE) = -2477.39194507 A.U. after 1 cycles

Zero-point correction= 0.419881 (Hartree/Particle)

Thermal correction to Energy= 0.449732

Thermal correction to Enthalpy= 0.450676

Thermal correction to Gibbs Free Energy= 0.354422

Sum of electronic and zero-point Energies= -2476.972064

Sum of electronic and thermal Energies= -2476.942213

Sum of electronic and thermal Enthalpies= -2476.941269

Sum of electronic and thermal Free Energies= -2477.037523

6.1.7.4 Computed Excitations of T_1 (Emission of T_1) of compound 3f-ss (uPBE1PBE/6-31G PCM toluene)**

```
Excited State   1:      Triplet-A      0.7782 eV 1593.26 nm  f=0.0000  <S**2>=2.000
    129 ->131         0.20202
    130 ->131         0.70266
    129 <-131         0.11126
    130 <-131         0.24156
This state for optimization and/or second-order correction.
Total Energy, E(TD-HF/TD-KS) =  -2477.40491166
Copying the excited state density for this state as the 1-particle RhoCI density.

Excited State   2:      Triplet-A      2.0005 eV  619.78 nm  f=0.0000  <S**2>=2.000
    129 ->131         0.28191
    129 ->132        -0.17267
    130 ->132         0.57179
```

6.1.7.5 Computed xyz-Coordinates of radical cation of compound 3f-ss (uB3LYP/6-311G*)

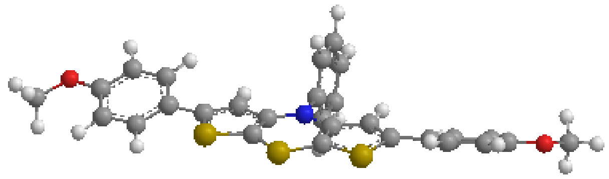

Figure S60. Optimized ground state geometry of radical cation of **3f-ss** (uB3LYP/6-311G* PCM CH_2Cl_2).

C	1.21492	-0.41250	0.01692
C	1.27599	-1.80792	0.01022
S	-0.06417	-2.90948	0.01366
C	-1.34338	-1.73798	0.03064
C	-1.20748	-0.34795	0.04302
N	0.02173	0.29616	0.03508
C	2.50512	0.17819	0.02655
C	3.53412	-0.73464	0.02103
S	2.91278	-2.38377	-0.00960
S	-3.00910	-2.22517	0.04338
C	-3.54083	-0.54474	0.03322
C	-2.46417	0.31120	0.04150
C	4.96770	-0.49300	0.01829
C	0.05994	1.74544	0.04552
C	-4.95819	-0.22190	0.03605
C	5.88393	-1.45709	0.48941
C	7.24065	-1.21305	0.48885
C	7.74267	0.01156	0.01500
C	6.84739	0.98148	-0.46059
C	5.48435	0.72394	-0.45652
C	0.07799	2.43756	-1.16348
C	0.11297	3.83048	-1.14800
C	0.12976	4.51642	0.06557
C	0.11211	3.81300	1.26908

C	0.07667	2.42001	1.26443
C	-5.40437	1.02961	0.51409
C	-6.74285	1.35695	0.51074
C	-7.69929	0.44397	0.03049
C	-7.27560	-0.80587	-0.44319
C	-5.92481	-1.12349	-0.43664
O	9.07957	0.15127	0.05713
C	9.67402	1.36706	-0.39772
O	-8.97707	0.86099	0.06984
C	-10.01304	-0.00731	-0.39114
H	2.66379	1.24530	0.07435
H	-2.56533	1.38589	0.00998
H	5.52625	-2.40119	0.88789
H	7.94422	-1.94913	0.85919
H	7.20410	1.92781	-0.84544
H	4.81394	1.47544	-0.85883
H	0.06269	1.89224	-2.10083
H	0.12543	4.37756	-2.08405
H	0.15618	5.60043	0.07346
H	0.12591	4.34640	2.21295
H	0.06285	1.86122	2.19384
H	-4.69246	1.74016	0.91887
H	-7.08872	2.31265	0.88663
H	-7.98506	-1.52804	-0.82511
H	-5.62238	-2.08644	-0.83614
H	10.74396	1.23794	-0.25637
H	9.46415	1.53610	-1.45763
H	9.32809	2.21988	0.19312
H	-10.93798	0.54789	-0.25834
H	-9.88149	-0.24970	-1.44935
H	-10.05003	-0.92557	0.20153

SCF Done: E(UB3LYP) = -2479.48581251 A.U. after 1 cycles

Zero-point correction= 0.417793 (Hartree/Particle)

Thermal correction to Energy= 0.447492

Thermal correction to Enthalpy= 0.448436

Thermal correction to Gibbs Free Energy= 0.353052

Sum of electronic and zero-point Energies= -2479.068019

Sum of electronic and thermal Energies= -2479.038320

Sum of electronic and thermal Enthalpies= -2479.037376

Sum of electronic and thermal Free Energies= -2479.132760

6.1.7.6 Computed xyz-Coordinates of dication (S_0) of compound 3f-ss (uB3LYP/6-311G*)

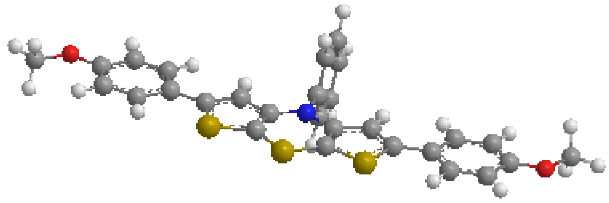

Figure S61. Optimized ground state geometry of dication of **3f-ss** (uB3LYP/6-311G* PCM CH_2Cl_2).

C	1.21910	-0.37505	0.00003
C	1.27559	-1.79408	0.00004
S	-0.06736	-2.83924	-0.00005
C	-1.34850	-1.71917	-0.00012
C	-1.21021	-0.30580	0.00003
N	0.02299	0.31744	0.00008
C	2.49665	0.19834	0.00004
C	3.53464	-0.72963	0.00006
S	2.89891	-2.38620	-0.00000
S	-3.00364	-2.21658	-0.00027
C	-3.54253	-0.52589	-0.00006
C	-2.45283	0.34012	0.00007
C	4.94705	-0.50828	0.00009

C	0.06371	1.77516	0.00015
C	-4.93779	-0.21465	-0.00011
C	5.87548	-1.58649	0.00051
C	7.22615	-1.36450	0.00047
C	7.73212	-0.04102	-0.00000
C	6.82870	1.04730	-0.00038
C	5.47326	0.80991	-0.00034
C	0.08156	2.45207	-1.21743
C	0.11810	3.84537	-1.20954
C	0.13591	4.53886	0.00028
C	0.11816	3.84526	1.21003
C	0.08161	2.45195	1.21779
C	-5.37863	1.13953	-0.00045
C	-6.71152	1.45043	-0.00045
C	-7.68876	0.42319	-0.00010
C	-7.27434	-0.92821	0.00022
C	-5.93088	-1.22694	0.00020
O	9.04863	0.06684	-0.00005
C	9.69176	1.35723	-0.00043
O	-8.94527	0.83038	-0.00010
C	-10.03454	-0.11520	0.00025
H	2.65566	1.26566	0.00011
H	-2.55126	1.41463	0.00025
H	5.52703	-2.61433	0.00094
H	7.93510	-2.18336	0.00081
H	7.19145	2.06645	-0.00074
H	4.80518	1.66262	-0.00069
H	0.06630	1.90289	-2.15271
H	0.13159	4.38619	-2.14889
H	0.16313	5.62261	0.00032
H	0.13170	4.38599	2.14943

H	0.06643	1.90270	2.15303
H	-4.65962	1.94946	-0.00077
H	-7.05307	2.47840	-0.00073
H	-7.99953	-1.73079	0.00048
H	-5.64651	-2.27453	0.00047
H	10.75561	1.14158	-0.00029
H	9.42541	1.91547	-0.89969
H	9.42530	1.91605	0.89845
H	-10.93441	0.49182	0.00021
H	-10.00209	-0.73328	-0.89861
H	-10.00188	-0.73286	0.89939

SCF Done: E(UB3LYP) = -2479.15864278 A.U. after 3 cycles

Zero-point correction= 0.418284 (Hartree/Particle)

Thermal correction to Energy= 0.448010

Thermal correction to Enthalpy= 0.448954

Thermal correction to Gibbs Free Energy= 0.354457

Sum of electronic and zero-point Energies= -2478.740359

Sum of electronic and thermal Energies= -2478.710633

Sum of electronic and thermal Enthalpies= -2478.709689

Sum of electronic and thermal Free Energies= -2478.804186

6.1.7.7 Reoptimization of compound 3f-ss (uB3LYP/6-311G*)

Compound **3f-ss** in the gas phase (uB3LYP/6-311G*):

SCF Done: E(UB3LYP) = -2479.68816136 A.U. after 1 cycles
Zero-point correction= 0.416387 (Hartree/Particle)
Zero-point correction= 0.416890 (Hartree/Particle)
Thermal correction to Energy= 0.446626
Thermal correction to Enthalpy= 0.447570
Thermal correction to Gibbs Free Energy= 0.352366
Sum of electronic and zero-point Energies= -2479.271272
Sum of electronic and thermal Energies= -2479.241535
Sum of electronic and thermal Enthalpies= -2479.240591
Sum of electronic and thermal Free Energies= -2479.335795

Compound **3f-ss** in CH_2Cl_2 (uB3LYP/6-311G* SMD CH_2Cl_2):

SCF Done: E(UB3LYP) = -2479.72783635 A.U. after 14 cycles

Radical cation of compound **3f-ss** in CH_2Cl_2 (uB3LYP/6-311G* SMD CH_2Cl_2):

SCF Done: E(UB3LYP) = -2479.56579036 A.U. after 20 cycles

Dication of compound **3f-ss** in CH_2Cl_2 (uB3LYP/6-311G* SMD CH_2Cl_2):

SCF Done: E(UB3LYP) = -2479.36192183 A.U. after 16 cycles

6.1.7.8 Computed xyz-Coordinates of transition state of the anisyl rotation of compound 3f-ss (PBE1PBE/6-31G** PCM CH_2Cl_2)

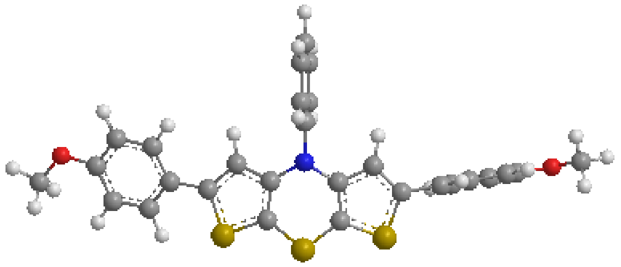

Figure S**62**. Optimized geometry of the transition state of the anisyl rotation of **3f-ss** (PBE1PBE/6-31G** PCM CH_2Cl_2).

C	-1.20275	-0.23500	-0.76645
C	-1.28035	-1.59067	-0.97585
S	0.00925	-2.55614	-1.69448
C	1.30335	-1.55048	-1.04921
C	1.19384	-0.19766	-0.83271
N	-0.02021	0.49128	-0.98814
C	-2.42691	0.30323	-0.26534
C	-3.40373	-0.63905	-0.09434
S	-2.82410	-2.22107	-0.52139
S	2.88467	-2.13428	-0.66419
C	3.43131	-0.53618	-0.24173
C	2.41343	0.37622	-0.37278

C	-4.78609	-0.45042	0.39004
C	-0.02934	1.91380	-0.84140
C	4.81038	-0.29814	0.18129
C	-5.08446	-0.52129	1.75865
C	-6.37810	-0.33442	2.21573
C	-7.41428	-0.07383	1.31012
C	-7.13489	-0.00220	-0.05719
C	-5.82873	-0.19250	-0.50187
C	-0.09026	2.50310	0.42346
C	-0.09358	3.89027	0.54058
C	-0.03006	4.68799	-0.60049
C	0.03580	4.09808	-1.86063
C	0.03485	2.71088	-1.98273
C	5.40659	0.96132	-0.00872
C	6.70361	1.20783	0.40182
C	7.45997	0.19543	1.00714
C	6.88954	-1.06590	1.19503
C	5.57891	-1.29655	0.78926
O	-8.64267	0.09273	1.85248
C	-9.72358	0.35347	0.97734
O	8.71899	0.53317	1.36925
C	9.52220	-0.46174	1.97544
H	-2.57951	1.35197	-0.04201
H	2.52120	1.42429	-0.12187
H	-4.28754	-0.72333	2.46845
H	-6.61242	-0.38610	3.27430
H	-7.91801	0.19962	-0.77881
H	-5.61722	-0.13580	-1.56580
H	-0.13421	1.87120	1.30591
H	-0.14289	4.34776	1.52412
H	-0.03137	5.76985	-0.50667

H	0.08558	4.71753	-2.75105
H	0.08253	2.23373	-2.95671
H	4.84821	1.75105	-0.50253
H	7.16180	2.18012	0.25050
H	7.44695	-1.86806	1.66445
H	5.14402	-2.27599	0.97163
H	-10.60705	0.44869	1.60887
H	-9.57482	1.28689	0.42196
H	-9.87369	-0.46892	0.26812
H	10.48301	0.01008	2.18182
H	9.08341	-0.81387	2.91643
H	9.67585	-1.31653	1.30638

SCF Done: E(RPBE1PBE) = -2477.45719525   A.U. after   1 cycles

Zero-point correction= 0.421950 (Hartree/Particle)

Thermal correction to Energy= 0.450633

Thermal correction to Enthalpy= 0.451577

Thermal correction to Gibbs Free Energy= 0.358402

Sum of electronic and zero-point Energies= -2477.035246

Sum of electronic and thermal Energies= -2477.006562

Sum of electronic and thermal Enthalpies= -2477.005618

Sum of electronic and thermal Free Energies= -2477.098793

6.2 DFT-Calculation of the redox potentials of compounds 3 and 6

In addition to the experimental determination via cyclic voltammetry the redox potentials were also calculated adapting a literature procedure (equation 3) based on DFT methods (Tables 4 and 5).[19]

$$E_{redox} = \frac{\Delta G_{redox}(solv)}{-F} = \frac{\Delta G_{redox}(gas)+\Delta G_{solv}(ox)-\Delta G_{solv}(red)}{-F} \quad \text{(eq. 3)}$$

E_{redox}	redox potential vs. vacuum
ΔG_{redox}(solv/gas)	free enthalpy of oxidation in solution/gas phase
ΔG_{solv}(ox/red)	free enthalpy of solvation of the oxidized/reduced compound
F	Faraday constant (23.06 kcal $mol^{-1}\cdot V^{-1}$)

ΔG_{solv}(gas), ΔG_{solv}(solv) and ΔG_{redox}(gas) were calculated from the values of the free enthalpies obtained from the geometry optimizations given in chapter 6.1 (uB3LYP/6-311G* SMD CH_2Cl_2). The SMD solvation model[18] with dichloromethane as a solvent was applied to determine the solvation enthalpies, since all experimental determined oxidation potentials were measured in dichloromethane solutions.

Table S4. Calculated ΔG_{solv}, ΔG_{redox} and $E_{redox}^{0/+1}$ of compounds **3** and **6** (uB3LYP/6-311G* SMD CH_2Cl_2).

Compound	$\Delta G_{redox}(gas)$ [kcal/mol]	$\Delta G_{solv}(red)$ [kcal/mol]	$\Delta G_{solv}(ox)$ [kcal/mol]	$\Delta G_{redox}(solv)$ [kcal/mol]	$E_{redox}^{0/+1}$ vs. vacuum [V]
**3a-aa**	160.1807	-26.1862	-64.3391	122.1807	5.3018
**3b-ss**	160.5927	-25.2716	-64.8558	121.0085	5.2509
**6**	165.8539	-27.3339	-68.3648	124.8231	5.4165
**3c-aa**	143.2015	-28.4902	-63.0470	108.6446	4.7144
**3d-ss**	143.6312	-27.6796	-62.6710	108.6398	4.7142
**3e-aa**	126.3086	-24.8821	-50.6263	100.5644	4.3638
**3f-ss**	127.0476	-24.8798	-50.1533	102.0476	4.4282

Table S5. Calculated ΔG_{solv}, ΔG_{redox} and $E_{redox}^{+1/+2}$ of compounds **3** and **6** (uB3LYP/6-311G* SMD CH_2Cl_2).

Compound	$\Delta G_{redox}(gas)$ [kcal/mol]	$\Delta G_{solv}(red)$ [kcal/mol]	$\Delta G_{solv}(ox)$ [kcal/mol]	$\Delta G_{redox}(solv)$ [kcal/mol]	$E_{redox}^{+1/+2}$ vs. vacuum [V]
**3a-aa**	245.6212	-64.3391	-159.041	150.9188	6.5488
**3b-ss**	247.3976	-64.8558	-161.2460	151.3976	6.5696
**6**	249.6894	-68.3648	-165.0930	152.9261	6.6375
**3c-aa**	223.9806	-63.0470	-151.1170	135.9105	5.8976
**3d-ss**	228.1522	-62.6710	-151.2974	139.1522	6.0382
**3e-aa**	200.8855	-50.6263	-127.5750	123.9366	5.3780
**3f-ss**	206.0458	-50.1533	-127.4740	128.7249	5.5858

The calculated redox potentials reproduce the experimental data correctly as indicated by linear correlation (Figure 63).

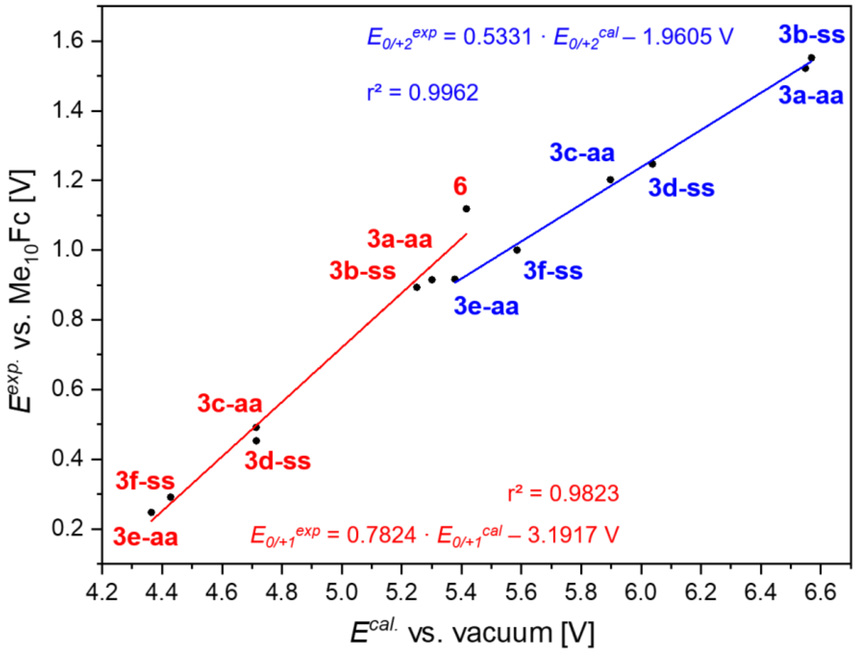

Figure S63. Correlation of the experimental oxidation potentials (0.1 M $[Bu_4N][PF_6]$, v = 100 mV/s, Pt-working, Ag/AgCl-reference and Pt-counter electrode, $[Me_{10}Fc]/[Me_{10}Fc]^+$ as an internal standard; $Me_{10}Fc$ = decamethylferrocene, $E_{0/+1}$ = -95 mV vs. ferrocene with $E_{0/+1}(Fc/Fc^+)$ = 450 mV) of the monooxidation $E_{0/+1}$ (red) and the dioxidation $E_{+1/+2}$ (blue) with the calculated redox potentials $E^{cal.}$ respectively (uB3LYP/6-311G* SMD CH_2Cl_2) of the compounds **3** and **6**.

7 References

[1] L. May, T. J. J. Müller, *Chem. Eur. J.* **2020**, *26*, *accepted for publication*. doi.org/10.1002/chem.202000137

[2] C. Dostert, C. Wansrath, W. Frank, T. J. J. Müller, *Chem. Commun.* **2012**, *48*, 7271–7273. DOI: 10.1039/c2cc32731g

[3] (a) M. V. Jovanovic, E. R. Biehl, *J. Org. Chem.* **1984**, *49*, 1905–1908; (b) C.-T. Li, F.-L. Wu, C.-J. Liang, K.-C. Ho, J. T. Lin, *J. Mater. Chem. A* **2017**,*5,*7586–7594. DOI: 10.1039/c6ta11091f

[4] R. Sens, K. H. Drexhage, *J. Luminesc.* **1981**, *24*, 709–712. DOI: 10.1016/0022-2313(81)90075-2

[5] J. M. Drake, M. L. Lesiecki, D. M. Camaioni, *Chem. Phys. Lett.* **1985**, *113*, 530–534.

[6] G. Jones, W. R. Jackson, C. Y. Choi, W. R. Bergmark, *J. Phys. Chem.* **1985**, 89, 294–300. DOI: 10.1021/j100248a024

[7] P. Zanello, in: *Ferrocenes* A. Togni (Ed.), T. Hayashi (Ed.), Wiley VHC, Weinheim **1995**, 317–430.

[8] For a detailed discussion of the *LiForK* sequence, see L. May, S. Daniel, T. J. J. Müller, *Org. Chem. Front.* **2020**, *7,* 329-339.

[9] For further dilithiation–lithium–zinc exchange–Negishi couplings of dithienothiazines, see: C. Dostert, T. J. J. Müller*, Org. Chem. Front*. **2015**, *2*, 481–491. DOI: 10.1039/c5qo00046g

[10] E. H. El-Mossalamy, A. Y. Obaid, S. A. El-Daly, I. S. El-Hallag, A. M. Asiri, L. M. *J. New Mater. Electrochem. Syst.* **2013**, *16*, 53–57. DOI: 10.14447/jnmes.v16i1.53

[11] E. Lippert, *Z. Elektrochem., Ber. Bunsenges. Phys. Chem.***1957**, *61*, 962−975.

[12] Gaussian 09, Revision A.02, M. J. Frisch, G. W. Trucks, H. B. Schlegel, G. E. Scuseria, M. A. Robb, J. R. Cheeseman, G. Scalmani, V. Barone, B. Mennucci, G. A. Petersson, H. Nakatsuji, M. Caricato, X. Li, H. P. Hratchian, A. F. Izmaylov, J. Bloino, G. Zheng, J. L. Sonnenberg, M. Hada, M. Ehara, K. Toyota, R. Fukuda, J. Hasegawa, M. Ishida, T. Nakajima, Y. Honda, O. Kitao, H. Nakai, T. Vreven, J. A. Montgomery, Jr., J. E. Peralta, F. Ogliaro, M. Bearpark, J. J. Heyd, E. Brothers, K. N. Kudin, V. N. Staroverov, R. Kobayashi, J. Normand, K. Raghavachari, A. Rendell, J. C. Burant, S. S. Iyengar, J. Tomasi, M. Cossi, N. Rega, J. M. Millam, M. Klene, J. E. Knox, J. B. Cross, V. Bakken,

C. Adamo, J. Jaramillo, R. Gomperts, R. E. Stratmann, O. Yazyev, A. J. Austin, R. Cammi, C. Pomelli, J. W. Ochterski, R. L. Martin, K. Morokuma, V. G. Zakrzewski, G. A. Voth, P. Salvador, J. J. Dannenberg, S. Dapprich, A. D. Daniels, O. Farkas, J. B. Foresman, J. V. Ortiz, J. Cioslowski, and D. J. Fox, Gaussian, Inc., Wallingford CT, 2009.

[13] a) M. Ernzerhof, G. E. Scuseria, *J. Chem. Phys.* **1999**, *110*, 5029–5036. DOI: doi.org/10.1063/1.478401; b) C. Adamo, G. E. Scuseria, V. Barone, *J. Chem. Phys.* **1999**, *111*, 2889–2899. DOI: doi.org/10.1063/1.479571

[14] (a) R. Krishnan, J. S. Binkley, R. Seeger, J. A. Pople, *J. Chem. Phys.* **1980**, *72*, 650–654. DOI: 10.1063/1.438955; (b) A. D. McLean, G. S. Chandler, *J. Chem. Phys.* **1980**, *72*, 5639–5648. DOI: 10.1063/1.438980.

[15] (a) Bauernschmitt R, Ahlrichs R., *Chem. Phys. Lett.* **1996**, *256*, 454–464. DOI: 10.1016/0009-2614(96)00440-X; (b) M. E. Casida, C. Jamorski, K. C. Casida, D. R. Salahub, *J. Chem. Phys.* **1998**, *108*, 4439–4449. DOI: 10.1063/1.475855; (c) R. E. Stratmann, G. E. Scuseria, M. J. Frisch, *J. Chem. Phys.* **1998**, *109*, 8218–8224. DOI: 10.1063/1.477483

[16] G. Scalmani, M. J. Frisch, *J. Chem. Phys.* **2010**, *132*, 114110–114115. DOI 10.1063/1.3359469

[17] (a) A. D. Becke, *J. Chem. Phys.* **1993**, *98*, 5648–5652. DOI: 10.1063/1.464913; (b) A. D. Becke, *J. Chem. Phys.* **1993**, *98*, 1372–1377, DOI: 10.1063/1.464304.

[18] A. V. Marenich, C. J. Cramer, D. G. Truhlar, *J. Phys. Chem. B* **2009**, *113*, 6378–6396. DOI: 10.1021/jp810292n

[19] J. Li, C. L. Fischer, J. L. Chen, D. *Bashford, Inorg. Chem.* **1996***, 35,* 4694–4702. DOI: 10.1021/ic951428f
